# Characterization and Identification of *Neocosmospora solani* and *Fusarium oxysporum* Causing Root Necrosis and Wilting of Orange Trees in Chile

**DOI:** 10.3390/plants14030376

**Published:** 2025-01-26

**Authors:** María A. Garzón-Nivia, Johanna Mártiz Mártiz, Ernesto A. Moya-Elizondo, Braulio Ruiz, Julio C. Cornejo, Héctor A. Valdés-Gómez

**Affiliations:** 1 Departamento de Fruticultura y Enología, Facultad de Agronomía y Sistemas Naturales, Pontificia Universidad Católica de Chile, Santiago 7820436, Chile; magarzon@uc.cl (M.A.G.-N.); jmartiz@uc.cl (J.M.M.); jscornejo@uc.cl (J.C.C.); 2 Departamento de Producción Vegetal, Facultad de Agronomía, Universidad de Concepción, Chillán 3780000, Chile; emoya@udec.cl (E.A.M.-E.); braruiz@udec.cl (B.R.)

**Keywords:** citrus, *Fusarium* spp., dry root rot, vascular wilt, phylogenetic analysis, morphology

## Abstract

Orange trees (*Citrus* × *sinensis* (L.) Osbeck) are the third-most cultivated citrus fruit species in Chile. In recent years, several trees in three orange orchards of ‘Lane late’ and ‘Fukumoto’ cultivars grafted on ‘Robidoux’ trifoliate orange (*Poncirus trifoliata* (L.) Raf.) have shown chlorosis, canopy reduction, wilting, root necrosis, defoliation, and plant death symptoms. This study aims to characterize the morphological symptoms observed in diseased orange trees in central Chile and identify the fungal pathogens that are involved. Isolation and morphological characterization of the pathogens were conducted by using different culture media. A total of 53 isolates were obtained, morphologically characterized and 12 isolates were selected for molecular identification. The isolates were identified using *ITS*, *TEF*-1α, and *RPB2* regions. Two *Fusarium* species complexes were identified, *Neocosmospora* (*Fusarium*) *solani* (FSSC) and *F. oxysporum* (FOSC), based on >99% identity. Pathogenicity tests were conducted on young orange seedlings under greenhouse conditions. Results indicated that two months post inoculation, trifoliate orange seedlings displayed root rot symptoms such as necrosis, vascular discoloration, and wilting. FSSC and FOSC were re-isolated from necrotic seedling roots and identified through a combination of morphological traits and molecular techniques. This is the first detailed report of this disease, attributed to FSSC and FOSC, in orange orchards in Chile. These diagnostic results represent the first step in developing adequate phytosanitary programs for managing this disease.

## 1. Introduction

The orange tree is one of the main fruit species cultivated in Chile, occupying a total area of 6362 ha and producing 131,262 tons annually, between the regions of Parinacota and Biobío [1]. Plants are highly susceptible to stressful conditions such as low temperatures, water deficit, and insufficient light, all of which are favorable for disease and pest development [2]. In recent years, symptoms such as chlorosis, canopy reduction, wilting, defoliation, and death have been recurrently observed in orange orchards in Central Chile. These symptoms resembled those caused by *Fusarium* spp. in citrus, which have been studied in different regions worldwide such as United States, Egypt, Italy, China, Turkey, and others [3,4,5,6,7], because dry root rot (DRR) has become an important threat to citrus production. However, there are no reports of *Fusarium* spp. in Chile, whereas in South America, this disease has only been reported in Brazil [2]. The spread of the disease could be associated with the reduction in precipitation by 25–45% in Chile during the last 10 years, which has increased water stress, especially in the central zone of Chile [8,9]. In addition, Mediterranean regions are susceptible to extreme changes in weather conditions; therefore, they are considered climate change hotspots that are favorable for disease development [10]. Moreover, reports on fungal diseases monitoring by ProMED-mail have increased by approximately 25% since 2015 [11], and metabarcoding studies have revealed that the increased incidence of pathogens such as *Alternaria*, *Fusarium*, and *Venturia* is due to increased temperature and CO_2_ [12].

*Fusarium* species can cause vascular wilt, root rot, crown canker, leaf lesions, rotting, and postharvest fruit decay in citrus plants. Species of this fungal genus can be free-living opportunistic pathogens, which are spread by water, wind, nematodes, and insects. The *Fusaria* members are characterized by the production of mycotoxins, antibiotics, and phytotoxins [13]. Various types of stress, which can promote the pathogenicity of these pathogens, have been reported in citrus. Abiotic stress factors include root asphyxia caused by excessive irrigation, excessive fertilization, drought, root wounds, and poor aeration, while interaction with other pathogens (*Phytophthora* spp., *Tylenchulus semipenetrans*, and Citrus Tristeza Virus (CTV)), insects, and rodents are biotic stress factors that favor *Fusarium* pathogens [14,15]. In addition, increased temperature (25–28 °C), high vapor water deficit, and soil water deficit are optimal conditions for the occurrence of symptoms associated with DRR [16].

In the absence of a host, *Fusarium* species survive in the soil using resistance structures, such as chlamydospores and free mycelium. Once environmental conditions become favorable, resistant forms germinate and colonize plant roots. Fungal hyphae penetrate the host through openings and natural lesions and colonize the vascular tissue, degrading the primary cell wall structures. This situation causes an interruption in the transport of nutrients and water and clogging of the vessels of both the roots and the aerial parts of the tree. This condition causes a significant decrease in plant yield, accelerating wilting and subsequent plant death [2].

At least 300 phylogenetically distinct species, 23 species complexes, and 9 monotypic lineages of *Fusarium* have been reported [17,18]. Most plant pathogens of the genus *Fusarium* are grouped into four complex species. The first is the *Fusarium solani* species complex (FSSC), comprising 60 phylogenetic species of *F. solani* responsible for causing diseases such as foot and root rot in various hosts [19]. Additionally, several *Neocosmospora* species have been isolated from the roots of diseased citrus plants with DRR. However, recent mycological studies have used the binomial scientific name *Neocosmospora solani* (SC) instead of *F. solani* [14,20,21]. The second is the *F. oxysporum* species complex (FOSC), which causes vascular wilt and root rot. The third is the *F. graminearum* species complex (FGSC) which causes significant damage to cereals and maize because of the ability to produce mycotoxins (trichothecene B and zearalenone) [22], and fourth is the *F. fukijuroi* species complex (FFSC) which causes diseases in several crops, such as maize, mango, rice, sugarcane, and pine [23].

In 1920, Barrett’s [4] pioneering work in California marked one of the earliest reports of *Fusarium* DRR affecting citrus plants. He described the symptomatology of dry rot and demonstrated that several types of dry rot are related to different species of the genus *Fusarium*. Subsequently, various studies have linked additional *Fusarium* species, such as *F. oxysporum*, *F. proliferatum*, and *F. sambucinum*, to the development of dry rot in citrus. The *F. incarnatum-equiseti* species complex (FIESC) and *F. citricola* species complex (FCCSC) have been described as citrus pathogens [14]. Although *F. equiseti* and *F. semitectum* have been identified in diseased citrus plants, their specific pathogenicity and impact on citrus health remain uncertain. However, studies have indicated that these two species primarily act as saprophytic colonizers, typically residing within diseased tissues as endophytes [2,24,25]. Sandoval-Denis et al. [14] determined three new species of *Fusarium*, the first two *F. citricola* and *F. salinense* belonging to the *F. citricola* species complex, and the third *F. siculi* belonging to the FFSC. These new species are responsible for crown cankers in several citrus species.

Considering the recurrent presence of symptoms associated with the damage caused by members of the *Fusarium* complexes in citrus orchards in Chile, this study aimed to identify the *Fusarium* species associated with the decline of orange orchards, due to symptoms of chlorosis, canopy reduction, wilting, defoliation, and plant death. This objective was achieved by examining the diseased plant material, conducting morphological characterization and genetic analysis with specific primers, and evaluating the ability of the isolates to colonize and induce root rot symptoms in healthy orange plants.

## 2. Results

### 2.1. Characterization of Disease Symptoms in Orchards

**Figure 1:**
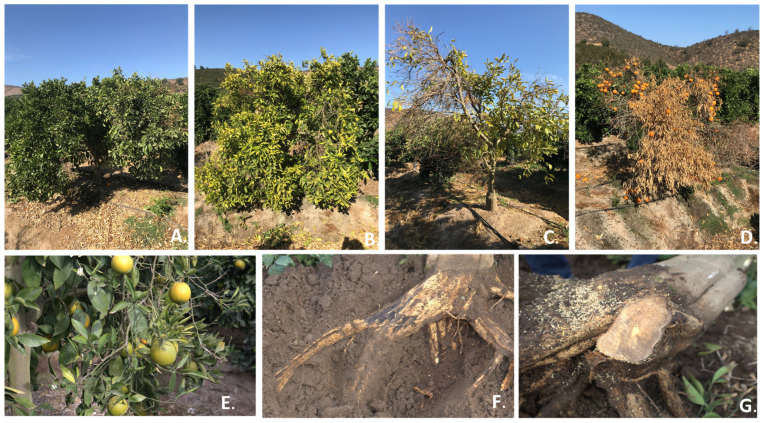
Symptomatology of orange trees in orchards in Melipilla, Metropolitan Region, Chile. (**A**,**B**) Sparse canopy and yellowing; (**C**) partial defoliation; (**D**) fruit persistence in the canopy with wilting leaves and advanced decline stage; (**E**) chlorosis and epinasty of citrus leaves; (**F**,**G**) root rot and vascular wilt.

The diseased trees in the three orange orchards evaluated in this study showed symptoms of root rot and vascular wilt, similar to those caused by *Fusarium* species in citrus plants from other countries. The main symptoms observed were wilting and yellowing of the leaves (Figure 1A,B), partial defoliation with the persistence of mature fruits in the canopy (Figure 1C,D), grayish-brown discoloration of the main root, and vascular wilt (Figure 1F,G). Finally, 7% of the orchard trees experienced total decline and death.

### 2.2. Morphological Characterization of Fusarium Species

Fifty-three isolates with characteristics of *Fusarium* spp. were isolated from infected orange tree roots and morphologically characterized as two possible *Fusarium* species: *N. solani* and *F. oxysporum*.

**Figure 2:**
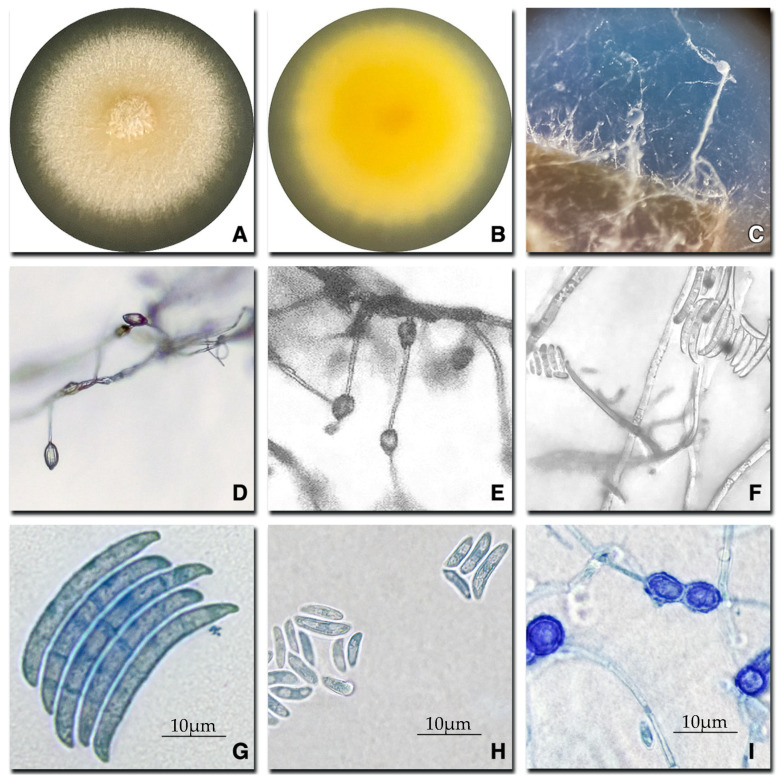
Morphological structures of *Neocosmospora* (*Fusarium*) *solani* isolated from orange trees. (**A**,**B**) Colonies on PDA after 7 days at 20 °C (view from upper site (**A**) and down site (**B**) of the petri dish); (**C**) aerial conidiophores formed on the surface of carnation leaves; (**D**–**F**) areal microconidia organized on mucilaginous false heads; (**G**,**H**) macro and microconidia; (**I**) Chlamydospores.

Colonies cultured on PDA of *Neocosmospora* (*Fusarium*) *solani* were yellow to beige in color and had a flat surface with a radial appearance and velvety texture, sparse or absent aerial mycelium, and irregular or filiform colony margins (Figure 2A). The reverse side of the plate had a yellow center and a beige edge (Figure 2B). No diffusible pigments were present in the medium. On CLA medium, the population of aerial conidiophores was abundant, simple, and unbranched, forming monophialides at the apices (Figure 2C,D), whereas phialides were thin, subcylindrical, monophialidic, and smooth-walled (Figure 2E). Macroconidia were vertically arranged in long basipetal chains, elongate, normal-shaped, papillate apical cell and foot-shaped, smooth-walled, thin-walled, septate basal cells 2–5 (182–204 × 15–39 µm) (Figure 2G,H). Microconidia, oval, 0–1 septate (65–111 × 33–40 µm) organized in false mucilaginous heads (Figure 2H). On SNA, hyaline hyphae and chlamydospores were present either singly or in pairs (Figure 2I).

**Figure 3:**
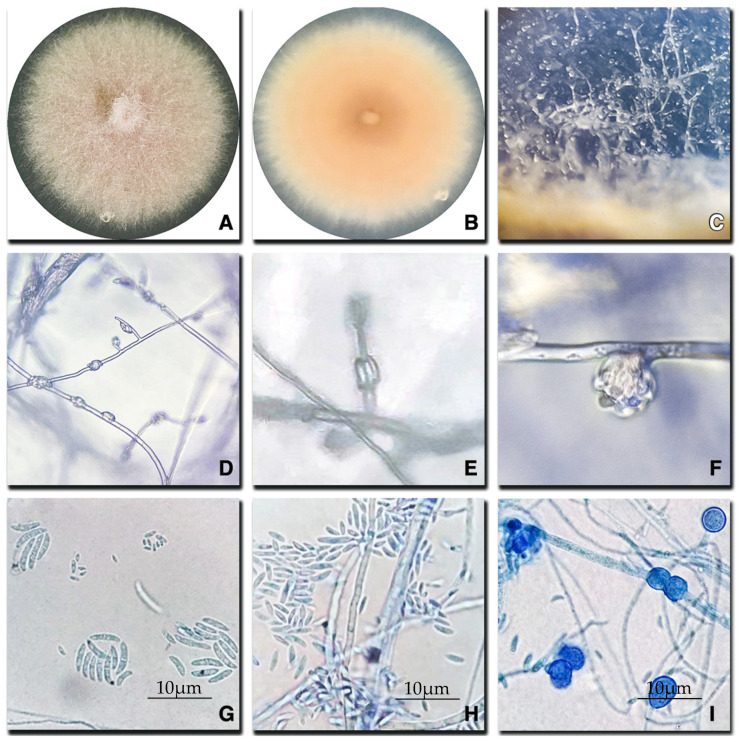
Morphological structures observed in *F*. *oxysporum* isolated from orange trees. (**A**,**B**) Colonies on PDA after 7 days at 20 °C (view from upper site (**A**) and down site (**B**) of the petri dish); (**C**) aerial conidiophores formed on the surface of carnation leaves; (**D**–**F**) areal microconidia organized on mucilaginous false heads; (**G**,**H**) macro and microconidia; (**I**) Chlamydospores.

*Fusarium oxysporum* colonies on PDA presented a purple/pink to white coloration, elevated cottony texture, abundant aerial mycelium, and lobulated margins (Figure 3A). Citric odors and no diffusible pigments were observed in the medium. The reverse-side purple center and pink-to-beige margin were observed in the PDA (Figure 3B). In CLA medium, sporulation of aerial conidiophores was abundant, simple, and unbranched, forming monophialides at the apices (Figure 3C,D); thin, subcylindrical, monophialidic, smooth-walled, while thin phialides were vertically arranged in short basipetal chains that rapidly collapsed into false mucilaginous heads (Figure 3E,F). Short macroconidia of coarse curved apical cells and barely notched basal cells with smooth, thin, septate (0–2) walls (101–193 × 36–45 µm) were observed (Figure 3G), whereas microconidia were oval and 0–1 septate (38–45 × 10–17 µm) (Figure 3H). In SNA medium, chlamydospores were present either singly or in pairs (Figure 3I).

### 2.3. Phylogenetic Identification

**Figure 4:**
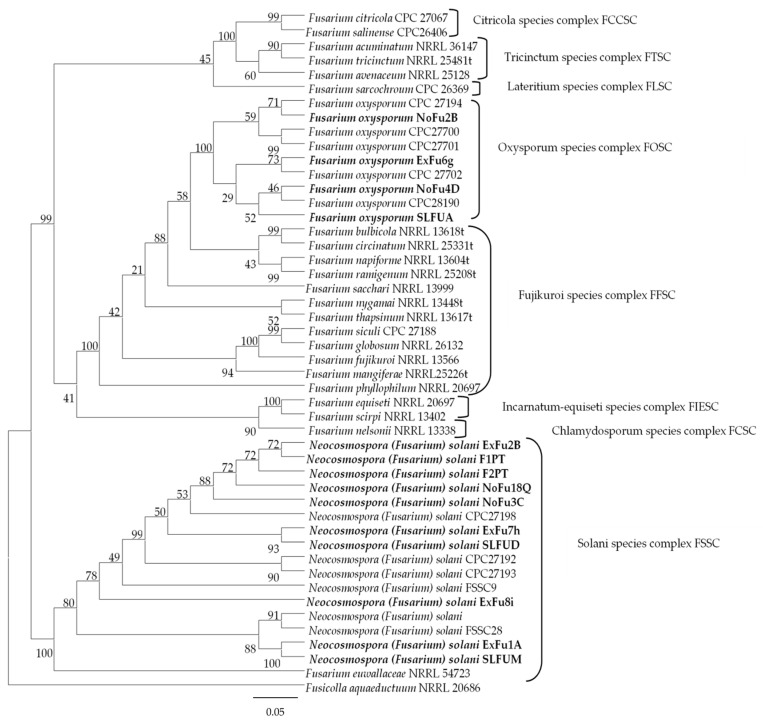
Phylogenetic tree construction using the Maximum Parsimony method based on a concatenated dataset of *TEF-1α*, *ITS*, and *RPB2* sequences of 47 strains belonging to the *Fusarium* species complex using MEGA v11. Support for the branches was evaluated using bootstrap analysis of 500 replications. Isolates obtained from orange trees are indicated in bold. The branch lengths were proportional to the distance. *Fusicolla aquaeductuum* (NRRL 20686) was used as an outgroup.

Phylogenetic analysis of the 12 isolates using specific primers confirmed that all the isolates belonged to the genus *Fusarium*. BLAST analysis revealed that the tree loci displayed a remarkably high level of identity with strains of *Neocosmospora solani* (syn. *F. solani*) species complex for eight isolates and with *Fusarium oxysporum* species complex for four isolates: *ITS*, *TEF1-α*, and *RPB2* showed 99% similarity. In addition, two isolates from the pathogenicity test, F1PT and F2PT, were added to the phylogenetic analysis to confirm the species (Figure 4). Also, the phylogenetic tree using the ex-epitype CBS 144134 for *F. oxysporum* proposed by Lombard et al. [19] constructed only with *TEF-1α* sequence showed similar results to previous analysis.

Based on morphological and phylogenetic analyses, we confirmed that *Fusarium* isolates associated with root rot and vascular wilt symptoms obtained from citrus roots in Chile correspond to the two aforementioned species of the fungal genus. *Neocosmospora* (*Fusarium*) *solani* was the most frequently identified isolate, followed by *F. oxysporum*. Information on the GenBank accession numbers of the *Fusarium* isolates is shown in Table S1.

### 2.4. Pathogenicity

**Figure 5:**
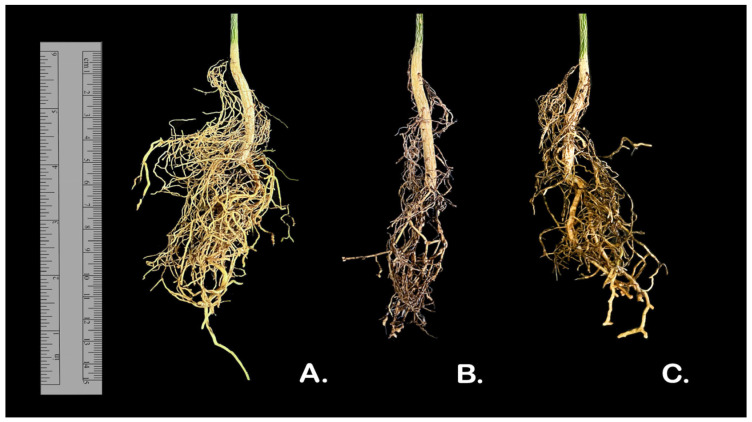
Necrosis generated by *Fusarium* spp. infection in three-month-old Rubidoux rootstock orange seedlings recovered 60 days post-inoculation. Uninoculated control (**A**), *Neocosmospora* (*Fusarium*) *solani* (**B**) and *F. oxysporum* (**C**).

The pathogenicity test was conducted using *Neocosmospora* (*Fusarium*) *solani* strain ExFu1A and *F. oxysporum* strain ExFu6G. This test revealed that the two tested strains were pathogenic to the ‘Rubidoux’ trifoliate orange rootstock. The inoculated seedlings exhibited the root rot symptoms of necrosis, vascular discoloration, and wilting (Figure 5). The fungi that emerged from the tissue sections were identified according to their morphology as *Fusarium*, with 93% re-isolation from necrotic root tissue and 68% re-isolation from stem pieces. These results confirmed the high virulence of these isolates. No symptoms were observed in the uninoculated control plants. Table 1 shows the effect these isolates have on the length of the root system, length of root necrosis, and the relationship between both variables in the plants 60 days after inoculation. Significant differences were observed between inoculated and uninoculated plants for necrotic root length (*p* = 0.0002) and the relationship between the principal root and necrotic root length (*p* = 0.0001).

## 3. Discussion

### 3.1. Fusarioid Species Affecting Orange Trees

This study identified pathogenic Fusarioid species associated with Citrus DRR in citrus orchards in Chile. Isolation, morphological characterization, and molecular identification of *Fusarium* and *Fusarium*-like species associated with root rot and vascular wilt were conducted in three Chilean orange orchards, and pathogenicity tests confirmed their involvement in the etiology DRR symptoms. Although symptoms of gummosis were occasionally observed in that season, typically associated with strains of *Phytophthora* spp. The rootstock *P. trifoliata* used is resistant to *Phytophthora*, but susceptible to *F. solani* [26,27]. Interestingly, some *Fusarium* species, such as *F. citricola* and *F. equiseti* were reported to generate gummosis symptoms in citrus and legumes [14,28].

Molecular characterization of obtained isolates revealed the presence of two complex species: *N. solani* (syn. *F. solani*) and *F. oxysporum*, which are associated with symptoms of root rot and vascular wilt.

Worldwide, various *Fusarium* species complexes are the most common pathogens found in citrus orchards and have been associated with several diseases, including wilt, canker damping-off, and root rot. Notably, *F. solani*, *F. oxysporum*, *F. proliferatum*, *F. sambucinum*, and *F. equiseti* are the most common [3,14,15,29]. In addition, several species of *Neocosmospora* are commonly found in orchard soils and citrus plants in Europe and South Africa, and are associated with DRR [14,30]. Recently, Crous et al. [20] argued for recognition of the *F. solani* species complex as a *Neocosmospora* genus; therefore, the *N. solani* binomial was used in this study. In addition, *N. solani* had been isolated from healthy roots of asymptomatic citrus trees and wilted roots from declined trees [30].

Pathogenicity testing of the two Fusarioid species was conducted to fulfill Koch’s postulates, and these trials showed that isolates can produce root rot and vascular wilt symptoms, including browning and discoloration. Similarly, the pathogenicity test revealed that the inoculated plants exhibited a higher percentage of root necrosis than the uninoculated control. This finding aligns with previous studies, which reported that seedlings inoculated with *Fusarium* species experienced reductions in root length, plant height, and both root and plant dry weights compared to healthy plants [3,5]. However, the results of these studies suggest the use of younger plants and increased stress conditions to reduce vigor, stem height, and foliar symptoms. Our findings are particularly interesting considering that numerous studies have indicated the challenges associated with artificially inducing *Fusarium* diseases symptoms. This process hinges on complex interactions between biotic and abiotic factors, and in some cases, it is not possible to reproduce the disease [31,32]. *Fusarium* species are commonly found in the soil of citrus orchards and nurseries as saprophytes, endophytes, or parasites, and play a key role in disease development [3,33]. However, some studies have demonstrated that certain less-virulent *Fusarium* species can symptomatically colonize citrus roots [34]. Other studies have confirmed that the inoculation of soil with *N.* (*Fusarium*) *solani* reduces the weight, root length, and stem height of citrus seedlings, decreasing seedling growth by 33% and reducing dry weight [31,35]. Our results showed that approximately 50% of the roots were affected by the two *Fusarium* species obtained in the survey, without significant differences between the two assessed Fusarioid species.

### 3.2. Potential Factors Contributing to Root Rot and Vascular Wilt

Citrus orchards in Chile are continuously under abiotic stress conditions. On the one hand, high temperatures occur in summer and excessive irrigation practices are used to counteract heat, and in other cases, drought conditions lead to periods of water deficit. Both scenarios can contribute to disease expression. Average temperatures have increased from 1.2 °C to 1.8 °C over the last decade, and droughts have occurred during the past 17 years, with precipitation deficits from 11% to 80% in Chile’s Metropolitan region [36,37,38]. Some reports have shown that the optimum temperatures for the development of *N. solani* and *F. oxysporum* are between 25 °C and 30 °C, and that growth is also significantly affected by water potential. In addition, the concentration of *N. solani* in the soil increases during months with high temperatures, intensifying the symptoms of DRR [39].

Chilean citrus field advisors have reported that the continued use of Rubidoux rootstock, which is susceptible to disease, could account for yield losses and a 20% increase in replanting efforts. Additionally, they estimated that approximately 10% of the national area dedicated to orange production was affected by symptoms associated to root rot and vascular wilt. Additionally, several authors have reported tree losses of 22% per year and fruit yield losses of 39.6% in other countries [6,40]. Rubidoux is one of the most widely planted rootstocks in Chile because it reduces plant size, it is resistant to Citrus Tristeza Virus and *Phytophthora* spp., waterlogging, and salinity, and improves fruit quality and yield [41]. However, this rootstock is susceptible to colonization by nematodes and is susceptibility to *Fusarium solani* [42]. Therefore, the study of alternative citrus rootstocks could offer valuable insights into managing *Fusarium* infections and enhancing plant tolerance to these pathogens.

A third contributing factor to the advancement of pathogen colonization is closely tied to the presence of other citrus soil pathogens, including *Phytophthora* spp. (mainly in orchards grafted in no-tolerant rootstock), *Tylenchulus semipenetrans*, CTV viruses, rodents, and insects [14,31,43]. Hence, effective management of these factors is essential for disease control.

Root rot and vascular wilt management of citrus crops is difficult for farmers. Therefore, increasing knowledge of the pathogen’s dissemination, spread, and infection and as well as the environmental factors that enhance its disease expression, will allow the development of adequate prevention, management, and control strategies. The implementation of integrated disease management programs, which consider proper: 1. cultural practices such as crop rotation and solarization, previous replant; 2. nutrient and water management, for instance, pressurized irrigation system and adequate drainage; 3. biological control using beneficial microorganisms as an example species of *Trichoderma* spp., *Bacillus* spp., and *Pseudomonas* spp.; 4. fungicide application (most used chemical group of benzimidazoles); 5. tolerant rootstock varieties have been suggested as an alternative to control this disease in citrus [2,15,44].

## 4. Materials and Methods

### 4.1. Field Sampling and Pathogen Isolation

The orchards selected for this study were located within the Melipilla province, in the Metropolitan Region of Chile, due to the significance of this area for the Chilean citrus industry. The province of Melipilla has a Mediterranean climate in which the growing degree days, absence of frost, and differential temperatures between day and night are favorable for tree phenological development and fruit production. In 2021, three commercial orange orchards (*Citrus* × *sinensis* (L.) Osbeck) of 5.9, 11.58, and 2.66 ha, located in the mentioned area (33°36′ S−71°08′ W, 33°43′ S−71°12′ W, and 33°42′ S−71°15′ W) and exhibiting symptoms such as chlorosis, canopy reduction, wilting, defoliation, and plant death, were selected for pathogen isolation (Figure 1). Each orchard had an average disease incidence of 13, 18, and 9%, respectively. In these orchards, three fields of the cv. Lane Late and Fukumoto, grafted onto ‘Robidoux’ trifoliate orange (*Poncirus trifoliata* (L.) Raf.) were selected for study. Ten orange trees from each field were randomly selected, and roots showing the typical symptoms of root rot and vascular wilt (purple coloration and vascular discoloration) were collected. Root samples were transported to the laboratory and stored at 4 °C until further use. This procedure was performed according to the protocol proposed by Leslie and Summerell [18]. First, the roots were rinsed with sterile distilled water to remove the soil particles. They were then cut into small pieces (3 × 3 mm), sterilized using 95% alcohol solution, and flamed in a Bunsen burner for 10 s. Tissue pieces were placed in potato dextrose agar (PDA) media supplemented with 1% lactic acid, also in selective *Fusarium* media (Glucose 20 g, KH_2_PO_4_ 0.5 g, NaNO_3_, MgSO_4_·7H_2_O 0.5 g, yeast extract 1 g, 1% FeSO_4_·7H_2_O 1 mL, agar 20 g) (SFA media) supplemented with pentachloronitrobenzene (PCNB 750 mg). Plant tissues were incubated in the two media at room temperature (~23 °C) for 7 days under dark conditions. Subsequently, fungal colonies growing from the tissue pieces were transferred to a new plate containing PDA medium.

### 4.2. Morphological Characterization and Identification

Morphological descriptions and identification were conducted following the guidelines established by Leslie and Summerell [24], and the findings were compared. Macroscopic characterization was performed by observing the growth of the isolated colonies on PDA and SFA. Morphological traits, including the texture of the colony, colony color, and agar pigmentation on PDA, were considered. Similarly, to observe and characterize the growth structures of the isolates, they were cultured on carnation leaf agar (leaf pieces on Petri dishes with 2% water agar [20 g L^−1^ agar]) (CLA media). Chlamydospores production was induced by cultivating the isolates on synthetic nutrient-poor Agar (SNA) (KH_2_PO_4_ 1 g, KNO_3_ 1 g, MgSO_4_*7H_2_O 0.5 g, KCl 0.5 g, Glucose 0.2 g, Sucrose 0.2 g, Agar 20 g). Incubation was performed at 20 °C for 10 days. For microscopic characterization of each isolate, microconidia/macroconidia production, number of septa, shape, and chlamydospore production were morphologically examined using a light microscope Olympus CX31RBSFA (Olympus Corporation, Tokyo, Japan). The length of the micro/macroconidia was measured using a microscope camera INFINITY5-5C (Teledyne Lumenera, Ontario, CA, USA), utilizing INFINITY ANALYZE v.7.0.2.920 image analysis software (Teledyne Lumenera). The fungal strains were stored in microtubes containing 25% glycerol at −20 °C stored in the Fruit Pathology Laboratory at the Pontificia Universidad Católica de Chile for further analysis.

### 4.3. DNA Extraction and Molecular Identification

Eight *Neocosmospora* (*Fusarium*) *solani* and four *F. oxysporum* isolates were grown in PDA culture medium for seven days at 20 °C using a 12 h/12 h photoperiod for DNA isolation, PCR, and sequencing. DNA was extracted from fresh mycelium extracted from the colony surface (100 mg) of each isolate using the modified CTAB method [45] using mechanical grinding with sterile plastic pestles, and 7.5 M ammonium acetate and isopropanol to precipitate DNA. The concentrations and purities of the samples were measured using a spectrophotometer. The samples were diluted to 10 ng µL^−1^ and stored at −80 °C until use. Fragments of the nuclear ribosomal internal transcribed spacer (*ITS*), elongation factor 1-alpha (*TEF 1α*), and the second subunit of polymerase (*RPB2*) genes were amplified using specific primers: ITS4 (5′GAAGTAAAAGTCGTAACAAGG3′)/ITS5 (5′TCCTCCGCTTATTGATATGC3′) [46] which amplified a 580 bp region, EF-1 (5′ATGGGTAAGGARGACAAGAC3′)/EF-2 (5′GGARGTACCAGTSATCATGTT3′) [47] which amplified a 750 bp region, and 7cF (5′ATGGGTAAGGARGACAAGAC3′)/11aR (5′GGARGTACCAGTSATCATGTT3′) [48] which amplified used 1000 bp region.

PCR amplifications were carried out using 1× buffer, 2.0 mM MgCl_2_, 0.2 mM dNTP, 0.2 µM of each partitioner, 0.5 U of Taq Platinum DNA Polymerase (Fermentas, Thermo Scientific™, Waltham, MA, USA), 20 ng fungal DNA, and nuclease-free water to obtain a final volume of 25 µL. Amplifications were performed using a MaxyGene™ II thermal cycler (Axygen^®^, Glendale, AZ, USA), and the amplification profiles of White et al. [46,48] PCR products were spotted on 1% agarose gels using 0.5 × TBE Buffer and SYBR^®^ Safe DNA gel stain visualization agent (Invitrogen, Thermo Scientific™, Waltham, MA, USA). 

Five microliters (5 µL) of PCR product was loaded with 1.5 µL of 6× DNA Loading Dye buffer and 5 µL of GeneRuler™ 1 Kb Plus DNA Ladder molecular weight marker (Thermo Scientific™, Waltham, MA, USA) was used. The PCR products of each isolate were sequenced using capillary electrophoresis (Macrogen Inc. Seoul, Republic of Korea). The sense and antisense sequences obtained for each fungus were aligned using the Needleman-Wunsch global alignment algorithm to obtain consensus sequences using Geneious 2024v.0.2 [49]. These sequences were compared with those in the FUSARIUM ID database using the BLAST local alignment algorithm and then deposited in NCBIs GenBank database (www.ncbi.nlm.nih.gov/ (accessed on 4 December 2023); accession numbers shown in Table S1). A phylogenetic tree based on alignment of the nucleotide sequences of *ITS*, *TEF-1 α*, and *RPB2* of different *Fusarium* species complexes and genera was constructed using MEGA v.11 (Figure S1) [50]. First, different phylogenetic analyses were conducted individually for each locus, followed by a multi-locus sequence analysis of the three loci, performed using Maximum Parsimony method [51]. *Fusiculla aquaeductuum* (NRRL20686) sequence from GenBank was selected as the outgroup. Support for the branches was evaluated using bootstrap analysis of 500 replications. On the other hand, phylogenetic analysis was performed including the ex-epitype of *F.oxysporum* (CBS 144134); a phylogenetic tree was constructed with the data of the *TEF-1 α* sequences (Figure S2).

### 4.4. Pathogenicity Tests

The pathogenicity test was carried out under controlled greenhouse conditions (25 °C and 16 h light/8 h dark) on seedlings (3 months old) of ungrafted Rubidoux rootstock oranges, and three replicates of four plants per treatment were implemented for a total of 36 plants. The plants were arranged in a completely randomized design. *N. solani* (ExFu1A) and *F. oxysporum* (ExFu6G) inoculums were prepared by solid fermentation of wheat by adding conidial suspension and incubated for seven days at 20 °C [52]. First, the solid substrate was prepared by autoclaving 60 g of wheat plus 50 mL of water (121 °C and 15 psi for 15 min). The method of preparation was to add a suspension of conidia of 19 mL on the solid substrate (wheat), 50 mL of 0.1% tween was added, left at room temperature for 7 days, 200 mL of 0.1% Tween was added, filtered, and the concentration of conidia was adjusted to 1 × 10^6^ conidia mL^−1^ for each evaluated isolate. Each plant was inoculated by dipping its roots into a 100 mL inoculum suspension for 30 min and then transplanted into 500 mL pots with sterile soil. Plants soaked in sterile distilled water were used as controls [5]. The plants were immediately irrigated to improve infection conditions and arranged in a completely randomized design. After two months, destructive sampling was performed to evaluate the length of the root system from the neck to the distal end and necrosis length. Roots were meticulously cut into small, uniform pieces (1 cm long), disinfected in a 2% sodium hypochlorite solution, and cultured in SFA medium for seven days at 20 °C. *Fusarium* colonies obtained from the culture medium were confirmed by macro and microscopic characterization. Two strains of *Fusarium* spp. (F1PT and F2PT) were molecularly identified using *ITS*, *TEF-1α*, and *RPB2* regions, as described above.

### 4.5. Statistical Analysis

Data from the pathogenicity test were summarized as mean ± standard deviation (SD). A normality test and a one-way analysis of variance (ANOVA) with a significance level of *p* ≥ 0.05 were carried out. When the effect was found to be significant, multiple comparisons (Tukey’s test) were performed using the InfoStat student version 2020 to access differences between the means of treatments.

## 5. Conclusions

In this study, it was confirmed that citrus root necrosis and wilting are complex diseases involving several Fusarioid species. The Fusarioid species identified in orange orchards of Chile were *N. solani* and *F. oxysporum*. Pathogenicity test demonstrated the virulence of these pathogens, causing root rot and wilting in inoculated potted plants. The ’Roubidoux’ trifoliate orange rootstock proved highly susceptible to these fungal pathogens. To our knowledge, this is the first detailed report of root rot and vascular wilt caused by these two Fusarioid species in orange trees in Chile.

## Figures and Tables

**Table 1:** Root length (RL), necrosis root length (NL), and NL/TL in Rubidoux rootstock orange seedlings 60 days post-inoculation with *Neocosmospora* (*Fusarium*) *solani* (ExFu1A) and *F. oxysporum* (ExFu6G) strains obtained from infected orange tree roots with symptoms of DDR (*n* = 3).

Isolates	Root Lenght (cm)	Necrotic Root Length (cm)	Percent Necrotic Length/Root Lenght NL/RL (%)
Uninoculated Control	15.6 ± 0.94 a	2.3 ± 0.04 a	15.1 ± 1.04 a
*N. solani* (syn. *F. solani*)	13.3 ± 0.24 a	6.9 ± 0.04 b	51.5 ± 3.00 b
*F. oxysporum*	13.3 ± 0.41 a	5.7 ± 0.61 b	42.9 ± 3.65 b

Results correspond to the average of three replicates, different letters (a, b) indicate statistically significant differences (*p* < 0.05; Tukey’s test; one-factor ANOVA) and ± correspond standard error value.

## Data Availability

Data will be made available upon reasonable request.

## Supplementary Materials

The following supporting information can be downloaded at: https://www.mdpi.com/article/10.3390/plants14030376/s1, Table S1. Origin, culture and GenBank accession numbers of fusaroid isolates and strains used for Phylogenetic analysis. Figure S1. Alignment of the nucleotide sequences of *ITS*, *RPB2*, and *TEF-1 α* of different *Fusarium* species complexes. Figure S2. Phylogenetic tree construction using the maximum parsimony method based on a concatenated dataset of *TEF-1α* sequence of 52 strains belonging to the *Fusarium* species complex using MEGA v11.
