# Supplementary material for: Characterization and Identification of Neocosmospora solani and Fusarium oxysporum Causing Root Necrosis and Wilting of Orange Trees in Chile
Source: Plants (Basel). 2025 Jan 26;14(3):376. doi: 10.3390/plants14030376 (PMC11821085; doi:10.3390/plants14030376)

Table S1. Origin, culture and GenBank accession numbers of fusaroid isolates and strains used for Phylogenetic analysis.

Species	Country/ location	Isolate	Host/ habitat	GenBank accession number		
				ITS	*tef.*-1 α	RPB2
F.citricola	Italy, Cosenza	CPC 27067	*Citrus lemon*	LT746242	LT746194	LT746307
F.salinense	Italy, Catania	CPC 26403	*Citrus sinensis*	LT746239	LT746191	LT746304
F.acuminatum	Unknown	NRRL 36147	Human bronchial secretion	GQ505452	GQ505420	GQ505484
F.tricinctum	Germany	NRRL 25481ᵀ	Wheat	HM068317	HM068307	HM068327
F. avenaceum	Poland	NRRL 25128	*Hymenoptera ichneumonidae*	JF740894	JF740751	JF741079
F.sarcochroum	Italy, Catania	CPC 26369	*Citrus lemon*	LT746255	LT746207	LT746320
F. oxysporum	Italy, Siracusa	CPC 27194	*Citrus sinensis*	LT746249	LT746201	LT746314
	Italy, Siracusa	CPC 27700	*Citrus sinensis*	LT746251	LT746203	LT746316
	Italy, Siracusa	CPC 27701	*Citrus sinensis*	LT746252	LT746204	LT746317
	Italy, Siracusa	CPC 27702	*Citrus sinensis*	LT746253	LT746205	LT746318
	Italy, Catania	CPC 28190	*Citrus sinensis*	LT746254	LT746206	LT746319
	Chile, Melipilla	ExFu6G	*Citrus sinensis*	OR879063	OR891727	OR891739
	Chile, Melipilla	NoFu2B	*Citrus sinensis*	OR879066	OR891730	OR891742
	Chile, Melipilla	NoFu4D	*Citrus sinensis*	OR879068	OR891732	OR891744
	Chile, Melipilla	SlFuA	*Citrus sinensis*	OR879070	OR891734	OR891746
F.bulbicola	Germany	NRRL 13618ᵀ	*Nerine bowdenii*	U61676	AF160294	KF466404
F. circinatum	USA, Monterrey	NRRL 25331ᵀ	Pine tree	NR120263	AF160295	JX171623
F. napiforme	Namibia	NRRL 13604ᵀ	*Pennisetum typhoides*	U34570	AF160266	EF470117
F. ramigenum	USA	NRRL 25208ᵀ	*Ficus carica*	NR111888	AF160267	KF466412
F. sacchari	India	NRRL 13999	*Saccharum officinarum*	U34556	AF160278	JX171580
F. nygami	Australia	NRRL 13448ᵀ	necrotic sorghum root	NR130698	AF160273	EF470114
F. thapsinum	Shout Africa	NRRL 22045	*Sorghum bicolor*	U34560	AF160270	JX171600
F. siculi	Italy, Catania	CPC 27188	*Citrus sinensis*	LT746262	LT746214	LT746327
F. globosum	Shout Africa	NRRL 26132	Zea mays seed	LT746278	LT746230	LT746343
F.fujikuroi	China	NRRL 13566	*Oryza sativa*	U34557	AF160279	JX171570
F. mangiferae	India	NRRL 25226ᵀ	*Mangifera indica*	U61691	AF160281	JX171622
F. phyllophilum	Italy	NRRL 13617ᵀ	*Dracaena deremensis*	U34574	AF160274	KF466410
F. equiseti	Chile	NRRL 20697	*Beta vulgaris*	GQ505683	GQ505594	JX171595
F. scirpi	Australia	NRRL 13402	pine nursery soil	GQ505681	GQ505592	JX171566
F.nelsonii	Australia	NRRL 13338	Soil	GQ505434	GQ505402	JX171561
N. solani	Italy, Catania	CPC 27198	*Citrus sinensis*	LT746271	LT746223	LT746336
	Italy, Siracusa	CPC 27192	*Citrus sinensis*	LT746269	LT746221	LT746334
	Italy, Siracusa	CPC 27193	*Citrus sinensis*	LT746270	LT746222	LT746335
	Italy, Siracusa	FSSC9- CPC27195	*Citrus sinensis*	LT746275	LT746227	LT746340
	Italy, Siracusa	FSSC28-CPC 28194	*Citrus sinensis*	LT746276	LT746228	LT746341
	Chile, Melipilla	ExFu1A	*Citrus sinensis*	OR879061	OR891725	OR891737
	Chile, Melipilla	ExFu2B	*Citrus sinensis*	OR879062	OR891726	OR891738
	Chile, Melipilla	ExFu7H	*Citrus sinensis*	OR879064	OR891728	OR891740
	Chile, Melipilla	ExFu8I	*Citrus sinensis*	OR879065	OR891729	OR891741
	Chile, Melipilla	NoFu3C	*Citrus sinensis*	OR879067	OR891731	OR891743
	Chile, Melipilla	NoFu18Q	*Citrus sinensis*	OR879069	OR891733	OR891745
	Chile, Melipilla	SlFuD	*Citrus sinensis*	OR879071	OR891735	OR891747
	Chile, Melipilla	SlFuM	*Citrus sinensis*	OR879072	OR891736	OR891748
F. euwallaceae	Isarrael	NRRL 54723	Avocado Tree	JQ038015	JQ038008	JQ038029
Fusicolla aquaeductuum	Germany	NRRL 20686	Water	-	-	JX171590

[1] ᵀEx-type strains; *F: Fusarium. N: Neocosmospora*

[2] EF-1α: Translation elongation factor 1-alpha; ITS: Internal transcribed spacer regions of the rDNA; RPB2: RNA polymerase second largest subunit.

Figure S1. Alignment of the nucleotide sequences of ITS, RPB2, and tef-1 α of different *Fusarium* species complexes.

>1 LT746194.1-*Fusarium citricola*
CG-CT----CCCATCGAAT---CCTACGACTCGCTCCA------TCACTCGAATCGCATCCA--TTACCCCGCTCGAGTCC-GAAAATTTTGCGGTG-
CGACCGTGAATTCTTTTT-GGTGGGGTATCTT-ACCCCGCCACTCGAGTGACGGATGCGCTTGCCCTGTTCCC-----ACAAAAT-CTCACTACCCT----
GCCGCGCACCAA-CATGT---CTTG--CAGTCACTAACCGTTG--
GACAATAGGAAGCCGCCGAGCTCGGAAAGGGTTCCTTCAAGTACGCCTGGGTTCTTGACAAGCTCAAAGCCGAGCGTGAGCGTGGTATCATGA
ACATACCTTTAATGTTGCCTCGGCGG--ATCAGCCC--GCGCCCCGTAA-AACGGGACGG---CCCGCCAGAGGAC--CCAAAC-TCTAATGTTTCT--
TATTGTAACTTCTGAGTAAAACAAACAAATAAATCAAAACTTTCAACAACGGATCTCTTGGTTCTGGCATCGATGAAGAACGCAGCAAAATGC
GATAAGTAATGTGAATTGCAGAATTCAGTGAATCATCGAATCTTTGAACGCACATTGCGCCCGCTGGTATTCCGGCGGGCATGCCTGTTCGAGC
GTCATTTC-AACCCTCAAGCCCCCGGGTT-TGGTGTTGGGGATCGGCT--CTGCCCTTCTGGGC---
GGTGCCGCCCCCGAAATACATTGGCGGTCTCGCTGCAGCCTCCATTGCGTAGTAGCTA-
ACACCTCGCAACTGGAACGCGGCGCGGCCATGCCGTAAAACC-
CCTGGTCAAAACGCTATCGTTGCAATTGCCTGTTACTCAGGATACAACCAAGAAGATTCCGTTATTATGAACCAGAGCAGTATTGATAGAGGTC
TATTCCGCAGTCTGTTCTTCCGATCGTACTCAGATCAGGAGAAGAAGGTCGGTCTCAACTATACAGAAATATTTGAGAAGCCTTTCCAGCAGAC
AACACTTCGAATGAAGCACGGAACATACGACAAGCTTGACGAAGATGGTATTGTGGCTCCTGGTGTACGAGTTTCTGGTGAAGATATCATCATT
GGTAAGACGGCGCCCATCGATCAGGAGAACCAGGATCTGGGTACCAGAACCCAATCGCACCAGCGCCGTGATATCTCGACACCGCTGCGAAG
TACAGAAAACGGTATCGTTGATCAAGTCATTCTGACAGTCAACGCCGACAACGTGAAGTACGTGAAGGTTCGAGTACGAACCACCAAGATTCC
CCAGATTGGTGACAAGTTTGCTTCTCGTCACGGTCAAAAGGGTACCATTGGTGTCACTTATCGCCAGGAAGATATGCCTTTCAGCAGAGAAGGC
CTGACTCCCGATATTATCATCAACCCTCACGCTATTCCCTCTCGAATGACAATTGCCCATTTGATTGAGTGTCTTCTTAGTAAGGTCTCAACACTT
GAAGGTATGGAGGGTGATGCAACACCATTCACCGATGTGACGGTCGACTCCGTCTCGGACCTTCT

>3 LT746201.1-*Fusarium oxysporum*
TC-CTT-TGCCCATCGATTT----------CC--CCTA------CGACTCGAAATGTGCCCG--CTACCCCGCTCGAGACC-AAAAATTTTGCAATA-
TGACCGTAATTTTTTTT--GGTGGGGCAC-TT-ACCCCGCCACTTGAGCGACGGGAGCGTTTGCCCTCTTAAC---------CATTCTCACAACCTCA--
ATGAGTGCGTCGT-CACGTG-TCAAG--CAGTCACTAACCATTC--
AACAATAGGAAGCCGCTGAGCTCGGTAAGGGTTCCTTCAAGTACGCCTGGGTTCTTGACAAGCTCAAGGCCGAGCGTGAGCGTGGTATCATGA
ACATACCAC-T-TGTTGCCTCGGCGG--ATCAGCCC--GCTCCCGGTAA-AACGGGACGG---CCCGCCAGAGGACCCCTAAAC-TC---TGTTTCT--
ATATGTAACTTCTGAGTAAAACCA-
TAAATAAATCAAAACTTTCAACAACGGATCTCTTGGTTCTGGCATCGATGAAGAACGCAGCAAAATGCGATAAGTAATGTGAATTGCAGAATT
CAGTGAATCATCGAATCTTTGAACGCACATTGCGCCCGCCAGTATTCTGGCGGGCATGCCTGTTCGAGCGTCATTTC-AACCCTCAAGCAC---
AGCT-TGGTGTTGGGACTCG------CG--TTAATTCG---------CGTTCCTCAAATTGATTGGCGGTCACGTCG-AGCTTCCATAGCGTAGTAGTAAAAC-
CCTCGTTACTGGTAATCGTCGCGGCCACGCCGTTAAACC-
CCCGGTCAAAACGCCATTGTTGCAATTGCTTGCTACTCAGGTTACAACCAGGAAGATTCCGTCATTATGAACCAGAGTAGTATTGATCGAGGTC
TGTTCCGAAGTCTGTTCTTCCGATCGTACTCAGATCAGGAGAAGAAGGTTGGTCTCAACTACACTGAGATCTTTGAGAAACCTTTCCAGCAGAC
AACGCTTCGAATGAAGCATGGAACATACGACAAGCTTGATGAAGATGGTATCGTGGCTCCTGGTGTCCGTGTGTCAGGTGAAGATATCATTATC
GGCAAGACTGCACCCATCGACCAAGAAAACCAGGACCTTGGCACAAGAACTCAATCGCACCAACGTCGTGATATCTCGACACCACTGCGAAG
TACTGAGAACGGTATCGTTGATCAAGTCATTCTGACAGTCAACGCCGATAACGTCAAGTACGTCAAGGTCCGAGTACGAACAACCAAGATTCC
TCAAATTGGTGACAAGTTTGCTTCTCGTCACGGTCAAAAGGGTACAATCGGTGTTACATATCGACAGGAGGATATGCCTTTCAGCCGGGAAGGT
CTTACTCCCGATATCATTATCAACCCTCACGCCATTCCATCGCGAATGACAATTGCCCATTTGATTGAGTGTCTTCTTAGCAAGGTTTCAACGCT
GGAAGGTATGGAGGGTGACGCCACACCGTTCACTGATGTCACAGTCGATTCAGTCTCAGAACTTCT

>4 LT746191.1-*Fusarium salinense*
CG-CT----CCCATCGAAT---CCTACGACTTGCTCCA------TCCCTCGAATCGCATCCA--TTTCCCCGCTCGAGTCC-CAAAATTTTGCGGTG-
CGACCGTGAATTCTTTTT-GGTGGGGTATCTT-ACCCCGCCACTCGAGTGACGGATGCGCTTGCCCTGTTCCC-----ACAAAAT-CTCACTACCCT----
GCCGCGCACTAT-CATGT---CTTG--CAGTCACTAACCATCG--
GACAACAGGAAGCCGCCGAGCTCGGAAAGGGTTCCTTCAAGTACGCCTGGGTTCTTGACAAGCTCAAAGCCGAGCGTGAGCGTGGTATCATG
AACATACCTTTAATGTTGCCTCGGCGG--ATCAGCCC--GCGCCCCGTAA-AACGGGACGG---CCCGCCAGAGGAC--CCAAAC-TCTAATGTTTCT--
TATTGTAACTTCTGAGTAAAACAAACAAATAAATCAAAACTTTCAACAACGGATCTCTTGGTTCTGGCATCGATGAAGAACGCAGCAAAATGC
GATAAGTAATGTGAATTGCAGAATTCAGTGAATCATCGAATCTTTGAACGCACATTGCGCCCGCTGGTATTCCGGCGGGCATGCCTGTTCGAGC
GTCATTTC-AACCCTCAAGCCCCCGGGTT-TGGTGTTGGGGATCGGCT--CTGCCCTCCTGGGC---
GGTGCCGCCCCCGAAATACATTGGCGGTCTCGCTGCAGCCTCCATTGCGTAGTAGCTA-
ACACCTCGCAACTGGAACGCGGCGCGGCCATGCCGTAAAACC-
CCTGGTCAAAACGCTATCGTTGCAATTGCCTGTTACTCAGGATACAACCAGGAAGATTCCGTTATTATGAACCAGAGCAGTATTGATAGAGGTC
TATTCCGCAGTCTGTTCTTCCGATCGTACTCAGATCAGGAGAAGAAGGTCGGTCTCAACTACACAGAAATATTTGAGAAGCCTTTCCAGCAGAC
AACACTTCGAATGAAGCACGGAACATACGACAAGCTTGACGAAGATGGTATTGTGGCTCCTGGTGTACGAGTTTCTGGTGAAGATATCATCATT
GGCAAGACGGCGCCCATCGATCAGGAGAACCAGGATCTGGGTACCAGAACCCAATCGCACCAGCGTCGTGATATCTCGACACCGCTGCGAAG
TACAGAAAACGGTATCGTTGATCAAGTCATTCTGACAGTCAACGCCGACAACGTGAAGTACGTGAAGGTTCGAGTACGAACCACCAAGATTCC
CCAGATTGGTGACAAGTTTGCTTCTCGTCACGGTCAAAAGGGTACCATTGGTGTCACTTATCGCCAGGAAGATATGCCTTTCAGCAGAGAAGGC
CTGACTCCCGATATTATCATCAACCCCCACGCTATTCCCTCTCGAATGACAATTGCCCATTTGATTGAGTGTCTTCTTAGTAAGGTCTCAACACTT
GAAGGTATGGAGGGTGATGCAACACCATTCACCGATGTGACGGTCGACTCCGTCTCGGACCTTCT

>5 LT746207.1-*Fusarium sarcochroum*
CT-TTG---CCCATCGATTTGTCCTACGAATCGCTTCC------TCA--CGACTCGCAACCTGCCTACCCCGCTCGAGTTC-AAAAATTTTGCGGTT-
CGACTGTAATTTTTT----GGTGGGGCGTTTT-ACCCCGCCACTCGAGCGATGG--
GCGCTTGCCCTGTTCCCTGCACACACAACACTCAATACCCATTTGGGCGCGCATCAT-CATGTGATCTGA--
CAGTTGCTAACCACCACCGACAATAGGAAGCCGCTGAGCTCGGAAAGGGTTCCTTCAAGTACGCCTGGGTTCTTGACAAGCTCAAAGCCGAGC
GCGAGCGTGGTATCATGAACATACCTT-ATTGTTGCCTCGGCGG--ATCAGCCC--GCTCCCCGTAA-AACGGGACGG---
CCCGCCAGAGGAAAACCAAAC-TCTATTGTATCA--T-
CTGTATCTTCTGAGTAACAAAAACAAATAAATCAAAACTTTCAACAACGGATCTCTTGGTTCTGGCATCGATGAAGAACGCAGCAAAATGCGA
TAAGTAATGTGAATTGCAGAATTCAGTGAATCATCGAATCTTTGAACGCACATTGCGCCCGCCAGTATTCTGGCGGGCATGCCTGTTCGAGCGT
CATTTC-AACCCTCAAGCCCCCGGGTT-TGGTGTTGGGGATCGGA---TTGCTAGT-----C---AGT-
CCGTCTCCGAAATCTAGTGGCGGTCTCGCTGCAGCCTCCATTGCGTAGTAGA-A-

ATATCTCGCAATCGGAACGCGGCGCGGCCATGCCGTTAAACAACCCGGGCAAAACGCCATTGTTGCCATTGCTTGTTATTCAGGTTACAACCAG
GAAGATTCCGTCATTATGAACCAGAGCAGTATCGATCGAGGTCTATTCCGCAGTCTGTTCTTCCGATCATACTCAGATCAAGAGAAGAAGGTTG
GTCTCAACTACACCGAAGTATTTGAGAAACCTTTCCAGCAGCAAACGCTTCGAATGAAGCACGGAACTTACGACAAGCTTGATGAGGATGGTA
TCGTGGCTCCTGGTGTGCGAGTGTCGGGTGAAGATATCATTATCGGCAAGACTGCACCGATCGACCAGGAGAACCAAGACCTAGGTGCCAGGA
CTCAAAGCCACCAGCGTCGCGATATCTCAACACCACTGCGAAGTACAGAGAACGGTATCATTGATCAAGTCATCATGACGGTCAACGCCGATA
ACGTCAAGTACGTCAAGGTCCGTGTGAGAACCACCAAGATTCCTCAGATTGGTGACAAGTTTGCCTCTCGTCACGGACAAAAGGGCACCATCG
GTGTCACCTACCGACAAGAAGATATGCCTTTTAGCAGAGAAGGTCTGACTCCAGATATCATCATCAACCCCCACGCCATTCCCTCGCGAATGA
CAATTGCCCATTTGATTGAGTGTCTACTCAGTAAGGTCTCAACACTCGAAGGTATGGAGGGTGACGCAACGCCTTTCACTGATGTTACGGTCGA
CTCCGTCTCGGACCTTCT

>6 LT746214.1-*Fusarium siculi*

TG-CTC-TGCCCCCCGATTT----------CC--CTTA------CGATTCGAAACGTGCCTG--GTACCCCGCTCGAGACC-AAAAATTTTGCGATA-
TGACCGTAATTTTTTTT--GGTGGGGCAT-TT-ACCCCGCCACTCGAGCGATGGGCGCGTTTTTGCCCTTT-----------CCTGTCCACAACCTCA--
ATGAGCGCATTGT-CACGTG-TCATG--CAGCCACTAACCATTC--
GACAATAGGAAGCCGCTGAGCTCGGTAAGGGTTCCTTCAAGTACGCCTGGGTTCTTGACAAGCTCAAGGCCGAGCGTGAGCGTGGTATCATGA
ACATACCAA-T-TGTTGCCTCGGCGG--ATCAGCCC--GCTCCCGGTAA-AACGGGACGG---CCCGCCAGAGGACCCCTAAAC-TC---TGTTTCT--
ATATGTAACTTCTGAGTAAAACCA-
TAAATAAATCAAAACTTTCAACAACGGATCTCTTGGTTCTGGCATCGATGAAGAACGCAGCAAAATGCGATAAGTAATGTGAATTGCAGAATT
CAGTGAATCATCGAATCTTTGAACGCACATTGCGCCCGCCAGTATTCTGGCGGGCATGCCTGTTCGAGCGTCATTTC-
AACCCTCAAGCCCTCGGGTT-TGGTGTTGGGGATCGG-----CGAGCCCTCGCGG---
CAAGCCGGCCCCGAAATCTAGTGGCGGTCTCGCTGCAGCTTCCATTGCGTAGTAGTAAAAC-
CCTCGCAACTGGTACGCGGCGCGGCCAAGCCGTTAAACC-
CCTGGCCAAAATGCCATTGTCGCAATTGCTTGCTACTCAGGTTATAACCAGGAAGATTCCGTCATTATGAACCAGAGTAGTATCGATCGAGGTC
TGTTCCGAAGTCTGTTCTTCCGATCGTACTCAGATCAAGAGAAGAAGGTCGGTCTCAACTACACTGAGATCTTCGAGAAGCCTTTCCAGCAGAC
AACACTCCGAATGAAGCATGGAACATACGACAAGCTTGATGAGGATGGTATCGTGGCGCCTGGTGTCCGTGTGTCAGGTGAGGATATCATTAT
CGGCAAGACTGCACCCATCGACCAAGAAAACCGAGACCTTGGCACAAGAACTCAATCGCATCAGCGTCGTGATATCTCGACACCACTGCGAA
GTACTGAGAACGGTATCGTTGATCAGGTCATTCTGACAGTCAACGCCGACAACGTCAAGTACGTCAAGGTTCGAGTACGAACCACCAAGATTC
CTCAAATTGGTGACAAGTTTGCTTCTCGTCACGGTCAAAAGGGTACAATCGGTGTTACATATCGACAGGAGGATATGCCTTTCAGCCGAGAAGG
TCTCACTCCCGATATCATTATCAACCCTCACGCCATTCCATCGCGAATGACAATTGCCCATTTGATTGAGTGTCTTCTTAGCAAGGTTTCAACGC
TGGAAGGTATGGAGGGTGATGCTACACCGTTCACTGATGTCACAGTCGATTCAGTCTCTGAACTTCT

>7 LT746220.1-*Neocosmospora solani*

TTGCTATTCCACATCGAATTCCCCGTCGAATTCCCTC-------CTCCGCGACACGCTCCGCGCCCGCTTCTCCCGAGTCCCAAAAATTTTGCGGTT-
CGACCGTAATTTTTTT---GGTGGGGCATCT--ACCCCGCCACTCGGGCGACGTTGGACAAAGCCCTGATCCCTGCACACAAAAA-
CACCAAATCCTCT-TGGCGCGCATCA----CGTGGTTCACAACAGACACTGACTGGTTC-
AACAATAGGAAGCCGCTGAGCTCGGCAAGGGTTCCTTCAAGTACGCCTGGGTTCTTGACAAGCTCAAGGCCGAGCGTGAGCGTGGTATCATGA
ACTTACCTATA-CGTTGCTTCGGCGGGAA-TAGACG----GCCCCGTAA-AACGGGCCGCC--CCCGCCAGAGGACCCT-TAAC-TC---
TGTTTCTTTTAGTGTATCTTCTGAGTAAAACAAGCAAATAAATTAAAACTTTCAACAACGGATCTCTTGGCTCTGGCATCGATGAAGAACGCAG
CGAAATGCGATAAGTAATGTGAATTGCAGAATTCAGTGAATCATCGAATCTTTGAACGCACATTGCGCCCGCCAGTATTCTGGCGGGCATGCCT
GTTCGAGCGTCATTAC-AACCCTCAGGCCCCCGGGCC-TGGCGTTGGGGATCGGCG--GAGC-
CCCCCGTGGGCACACGCCGTCCCCCAAATACAGTGGCGGTCCCGCCGCAGCTTCCATCGCGTAGTAGCTA-
ACACCTCGCGACTGGAGAGCGGCGCGGCCACGCCGTAAAACACCCCGGTCAGAACGCCATCGTCGCTATCGCTTGTTACTCTGGTTACAACCA
GGAAGATTCCGTCATTATGAACCAGAGTAGTATCGATCGAGGCCTGTTCCGCAGTCTGTTCTTCAGATCCTACTCTGACCAGGAGAAGAAGGTC
GGTCTCAACTACACGGAAGTGTTTGAGAAGCCCTTCCAGCAGTCGACGCTTCGTATGAAGCACGGTACCTACGACAAGCTGGATGAGGATGGT
ATCGTGGCTCCCGGTGTGCGAGTGTCCGGTGAAGATATCATCATCGGCAAGACTGCGCCGATAGATCAAGAGAACCAGGATCTGGGTACCAGG
ACAACGGTGCACCAGCGTCGTGATATCTCCACGCCGCTGCGAAGTACCGAGAACGGTATCGTCGATTCGGTCATTGTGACTGTCAATGCCGAC
AACGTCAAGTATGTCAAGGTCCGTGTGAGGACGACCAAGATTCCCCAGATTGGTGACAAGTTCGCCTCTCGTCACGGACAGAAGGGTACCATT
GGTGTTACCTACCGACAGGAGGATATGCCCTTCAGCAGGGAGGGTGTGACACCAGACATTATCATTAACCCCCACGCCATTCCCTCGCGAATG
ACAATTGCCCATTTGATTGAATGCCTCCTCAGTAAGGTGTCAACGCTCGAAGGCATGGAGGGTGATGCCACCCCTTTCACCGATGTCACTGTCG
ACTCCGTTTCGGAACTGCT

>8 GQ505420.1-*Fusarium acuminatum*

CG-CT----CCCATCGATT---CCCACGATTCGCTCCC------TCACTCGAAACACATCCA--TTACCCCGCTCGAGTCC-GAAAATTTTGCGGTG-
CGACCGTGATTTTTT-CT-GGTGGGGTATCTT-ACCCCGCCACTCGAGTCACGGATGCGCTTGCCCTGTTCCC-----ACAAAAC-CTTACCACCCT----
GTCGCGCACTA--CATGT---CTTG--CAGTCACTAACCACTG--
GACAATAGGAAGCCGCCGAGCTCGGAAAGGGTTCCTTCAAGTACGCCTGGGTTCTTGACAAGCTCAAAGCCGAGCGTGAGCGTGGTATCATGA
ACATACCTT-AATGTTGCCTCGGCGG--ATCAGCCC--GCGCCCCGTAA-AACGGGACGG---CCCGCCAGAGGAC--CCAAAC-TCTAATGTTTCT--
TATTGTAACTTCTGAGTAAAACAAACAAATAAATCAAAACTTTCAACAACGGATCTCTTGGTTCTGGCATCGATGAAGAACGCAGCAAAATGC
GATAAGTAATGTGAATTGCAGAATTCAGTGAATCATCGAATCTTTGAACGCACATTGCGCCCGCTGGTATTCCGGCGGGCATGCCTGTTCGAGC
GTCATTTC-AACCCTCAAGCCCCCGGGTT-TGGTGTTGGGGATCGGCT--CTGCCCTCCTGGGC---
GGTGCCGCCCCCGAAATACATTGGCGGTCTCGCTGCAGCCTCCATTGCGTAGTAGCTA-
ACACCTCGCAACTGGAACGCGGCGCGGCCATGCCGTAAAACC-
CCTGGTCAAAACGCCATCGTCGCAATTGCCTGTTACTCAGGATACAACCAGGAAGATTCCGTTATTATGAACCAGAGCAGTATCGATAGAGGT
CTATTCCGCAGTCTGTTCTTCCGATCATACTCAGATCAGGAGAAGAAGGTCGGTCTCAACTACACAGAAATCTTTGAGAAGCCCTTCCAGCAGA
CAACACTTCGAATGAAGCACGGAACATACGACAAGCTTGACGAAGATGGTATCGTGGCCCCTGGTGTACGAGTTTCTGGTGAAGATATCATTA
TTGGCAAGACGGCGCCCATCGATCAGGAGAACCAGGATCTGGGTACCAGAACCCAATCGCACCAGCGTCGCGATATCTCGACACCGCTGCGA
AGTACAGAAAACGGTATCGTTGATCAAGTCATTCTGACAGTCAACGCCGATAACGTGAAGTACGTTAAGGTTCGTGTACGAACCACCAAGATT
CCCCAGATTGGTGACAAGTTTGCTTCTCGTCACGGTCAAAAGGGTACCATTGGTGTCACTTACCGACAGGAGGATATGCCTTTCAGCAGAGAAG
GCCTGACTCCCGATATTATTATCAACCCTCACGCCATTCCCTCTCGAATGACAATTGCCCATTTGATTGAGTGTCTTCTTAGTAAGGTCTCAACA
CTTGAAGGTATGGAGGGTGATGCAACGCCATTCACTGATGTGACAGTCGACTCTGTCTCAGAGCTTTT

>9 JF740751.1-*Fusarium avenaceum*

CG-CT----CCCATCGATT---CCCACGACTCGCTCCC------TCATTCGAAACGCATTCA--TTACCCCGCTCAAGTCC-GAAAATTTTGCGGTG-
CGACCGTGATTTTTT-TT-GGTGGGGTATCTT-ACCCCGCCACTCGAGTGACGGATGTGCTTGCCCTGTTCCC-----ACAAAAC-CTTACCACACT----
GTCGCGCACT----ATGT---CTTG--CAGTCACTAACCACTG--
GACAATAGGAAGCCGCCGAGCTCGGAAAGGGTTCCTTCAAGTACGCCTGGGTTCTTGACAAGCTCAAAGCCGAGCGTGAGCGTGGTATCATGA
ACATACCTT-AATGTTGCCTCGGCGG--ATCAGCCC--GCGCCCCGTAA-AACGGGACGG---CCCGCCAGAGGAC--CCAAAC-TCTAATGTTTCT--
TATTGTAACTTCTGAGTAAAACAAACAAATAAATCAAAACTTTCAACAACGGATCTCTTGGTTCTGGCATCGATGAAGAACGCAGCAAAATGC
GATAAGTAATGTGAATTGCAGAATTCAGTGAATCATCGAATCTTTGAACGCACATTGCGCCCGCTGGTATTCCGGCGGGCATGCCTGTTCGAGC
GTCATTTC-AACCCTCAAGCCCCCGGGTT-TGGTGTTGGGGATCGGCT--CTGCCTTCT--GGC---
GGTGCCGCCCCCGAAATACATTGGCGGTCTCGCTGCAGCCTCCATTGCGTAGTAGCTA-
ACACCTCGCAACTGGAACGCGGCGCGGCCATGCCGTAAAACC-
CCTGGTCAAAACGCCATCGTCGCAATTGCCTGTTACTCAGGATACAACCAGGAAGATTCCGTTATTATGAACCAGAGCAGTATCGATAGAGGT
CTATTCCGCAGTCTGTTCTTCCGATCGTACTCAGATCAGGAGAAGAAGGTCGGTCTCAACTACACAGAAATCTTTGAGAAGCCTTTCCAGCAGA
CAACACTTCGAATGAAGCACGGAACATACGACAAGCTTGACGAGGATGGTATTGTGGCCCCTGGTGTACGAGTTTCTGGTGAAGATATCATTA
TCGGCAAGACGGCGCCCATCGACCAGGAGAACCAGGATCTGGGTACCAGAACCCAATCGCACCAGCGTCGCGATATCTCGACACCACTGCGA
AGTACAGAAAACGGTATCGTTGATCAAGTCATTCTGACAGTCAACGCCGACAACGTGAAGTACGTCAAGGTTCGTGTACGAACCACCAAGATT
CCCCAGATTGGTGACAAGTTTGCTTCTCGTCACGGTCAAAAGGGTACCATTGGTGTTACCTATCGACAGGAGGATATGCCTTTCAGCAGAGAAG
GCCTGACTCCCGATATTATTATCAACCCTCACGCCATTCCCTCTCGAATGACAATTGCCCATTTGATTGAGTGTCTTCTTAGTAAGGTCTCAACA
CTTGAAGGTATGGAGGGTGACGCAACGCCATTCACTGATGTGACGGTCGACTCCGTCTCAGATCTCCT
>10 AF160294.1-*Fusarium bulbicola*
TC-CTT-TGCCCATCGGATT----------CT-CCATA------CGACTCGAAACGTGCCCG--CTACCCCGCTCGAGACC-AAAAATTTTGCGATA-
TGACCGTAATTTTTTT---GGTGGGGCAT-TT-ACCCCGCCACTCGAGCGATGCG--CGTTTCTGCCCTCT-----------CATTTCCACAACCTT---
CTGAGCGCATCGT-CACGTG-TTAAG--CAGTCACTAACCATTC--
GACAATAGGAAGCCGCTGAGCTCGGTAAGGGTTCCTTCAAGTACGCCTGGGTTCTTGACAAGCTCAAGGCCGAGCGTGAGCGTGGTATCATGA
ACATACCAA-T-TGTTGCCTCGGCGG--ATCAGCCC--GCTCCCGGTAA-AACGGGACGG---CCCGCCAGAGGACCCCTAAAC-TC---TGTTTCT--
ATATGTAACTTCTGAGTAAAACCA-
TAAATAAATCAAAACTTTCAACAACGGATCTCTTGGTTCTGGCATCGATGAAGAACGCAGCAAAATGCGATAAGTAATGTGAATTGCAGAATT
CAGTGAATCATCGAATCTTTGAACGCACATTGCGCCCGCCAGTATTCTGGCGGGCATGCCTGTTCGAGCGTCATTTC-AACCCTCAAGCCC---
AGCT-TGGTGTTGGGACTCG------CGAGTCAAATCG---------CGTTCCCCAAATTGATTGGCGGTCACGTCG-
AGCTTCCATAGCGTAGTAGTAAAAC-CCTCGTTACTGGTAATCGTCGCGGCCACGCCGTTAAACC-
CCTGGTCAAAATGCCATTGTCGCAATTGCCTGCTACTCAGGTTACAACCAGGAAGATTCCGTCATTATGAACCAGAGTAGTATTGATCGAGGTC
TGTTCCGAAGCCTGTTCTTCCGATCGTACTCAGATCAAGAGAAGAAGGTTGGTCTCAACTACACTGAGATCTTTGAGAAGCCTTTCCAGCAGAC
AACGCTTCGCATGAAGCATGGAACATACGATAAGCTTGATGAGGATGGTATCGTGGCCCCTGGTGTCCGTGTGTCAGGTGAAGATATCATTATC
GGCAAGACTGCACCCATCGACCAAGAAAACCAGGACCTTGGCACAAGAACTCAATCGCATCAACGTCGTGATATCTCGACACCACTGCGAAG
TACTGAGAACGGTATCGTTGATCAAGTCATTCTGACAGTCAACGCCGATAACGTGAAGTACGTCAAGGTCCGAGTACGAACCACCAAGATTCC
TCAAATTGGTGACAAGTTTGCTTCTCGTCACGGTCAGAAGGGTACAATCGGTGTTACATATCGACAGGAGGATATGCCTTTCAGCCGAGAAGGT
CTTACTCCCGATATCATCATCAACCCTCACGCCATTCCGTCGCGAATGACAATTGCCCATTTGATTGAGTGTCTTCTTAGCAAGGTTTCAACGCT
GGAAGGTATGGAGGGTGACGCTACACCGTTCACTGATGTCACAGTCGATTCAGTCTCAGAACTTCT
>11 AF160295.1-*Fusarium circinatum*
TC-CTT-TGCCCATCG-ATT----------CT-CCATA------CGACTCGAAACGTGCCCG--CTACCCCGCTCGAGACC-AAAAATTTTGCGATA-
TGACCGTAATTTTTTTT--GGTGGGGCAT-TT-ACCCCGCCACTCGAGCGATGCG--CGTTTCTGCCCTCC-----------CATTGCCACAACCTT---
CTGAGCGCATCGT-CACGTG-TTAAG--CAGTCACTAATTATTC--
GACAATAGGAAGCCGCTGAGCTCGGTAAGGGTTCCTTCAAGTACGCCTGGGTTCTTGACAAGCTCAAGGCCGAGCGTGAGCGTGGTATCATGA
ACATACCAA-T-TGTTGCCTCGGCGG--ATCAGCCC--GCTCCCGGTAA-AACGGGACGG---CCCGCCAGAGGACCCCTAAAC-TC---TGTTTCT--
ATATGTAACTTCTGAGTAAAACCA-
TAAATAAATCAAAACTTTCAACAACGGATCTCTTGGTTCTGGCATCGATGAAGAACGCAGCAAAATGCGATAAGTAATGTGAATTGCAGAATT
CAGTGAATCATCGAATCTTTGAACGCACATTGCGCCCGCCAGTATTCTGGCGGGCATGCCTGTTCGAGCGTCATTTC-AACCCTCAAGCCC---
AGCT-TGGTGTTGGGACTCG------CGAGTTAAATCG---------CGTTCCCCAAATTGATTGGCGGTCACGTCG-AGCTTCCATAGCGTAGTAGTAAAAC-
CCTCGTTACTGGTAATCGTCGCGGCCACGCCGTTAAACC-
CCTGGCCAAAATGCCATTGTCGCAATTGCCTGCTACTCAGGTTATAACCAGGAAGATTCTGTTATTATGAACCAGAGTAGTATTGATCGAGGTC
TGTTCCGAAGCCTGTTCTTCCGATCGTACTCAGATCAAGAGAAGAAGGTTGGTCTCAACTACACTGAGATCTTTGAGAAGCCTTTCCAGCAGAC
AACGCTTCGCATGAAGCATGGAACATACGATAAGCTTGATGAGGATGGTATCGTGGCCCCTGGTGTCCGTGTGTCAGGTGAAGATATCATTATC
GGCAAGACTGCACCCATCGACCAAGAAAACCAGGACCTTGGCACAAGAACTCAATCGCATCAACGTCGTGATATCTCGACACCACTGCGAAG
TACTGAGAACGGTATCGTTGATCAAGTCATTCTGACAGTCAACGCCGATAACGTGAAGTACGTCAAGGTCCGAGTACGAACCACCAAGATTCC
TCAAATTGGTGACAAGTTTGCTTCTCGTCACGGTCAAAAGGGTACAATCGGTGTTACATATCGACAGGAGGATATGCCTTTCAGCCGAGAAGGT
CTTACTCCCGATATCATCATCAACCCTCACGCCATTCCGTCGCGAATGACAATTGCCCATTTGATTGAGTGTCTTCTTAGCAAGGTTTCAACGCT
GGAAGGTATGGAGGGTGACGCTACACCGTTCACTGATGTCACAGTCGATTCAGTCTCAGAACTTCT
>12 GQ505594.1-*Fusarium equiseti*
CC-CTC-TGCACACCGATCCATCACTCGAATCAGCCT----CGATGACTGAATATGCGCCTG--TCACCCCGCTCGAATAC-AAAA-TTTTGCGGTT-
CAACCGCAATTTTTT----GGTGGGGCTC-AT-ACCCCGCTGCTCGAGTGACAGGCGCTTGCCCTCTTCCCAC---------AAAA----TCACCTCTT---
GCGCG-----T-CACGTG-TCAAT--CAGTCACTAACCACCC--
GACAATAGGAAGCCGCCGAGCTCGGTAAGGGTTCCTTCAAGTACGCTTGGGTTCTTGACAAGCTCAAGGCCGAGCGTGAGCGTGGTATCATGA
ACATACCTA-TACGTTGCCTCGGCGG--ATCAGCCC--GCGCCCCGTAA-AACGGGACGG---CCCGCCCGAGGACCC-TAAAC-TC---TGTTTTT--A-
GTGGAACTTCTGAGTAAAACAAACAAATAAATCAAAACTTTCAACAACGGATCTCTTGGTTCTGGCATCGATGAAGAACGCAGCAAAATGCG
ATAAGTAATGTGAATTGCAGAATTCAGTGAATCATCGAATCTTTGAACGCACATTGCGCCCGCCAGTATTCTGGCGGGCATGCCTGTTCGAGCG
TCATTTC-AACCCTCAAGCTC---AGCT-TGGTGTTGGGACTCG------CG--GTAACCCG---------CGTTCCCCAAATCGATTGGCGGTCACGTCG-
AGCTTCCATAGCGTAGTAATAATACACCTCGTTACTGGTAATCGTCGCGGCCACGCCGTAAAACC-
CCCGGTCAAAACGCCATTGTTGCTATTGCTTGTTATTCAGGATACAACCAGGAAGACTCCGTCATTATGAATCAGAGCAGTATTGATCGAGGCC
TGTTCCGCAGTCTCTTCTTCCGATCATACTCGGATCAGGAGAAGAAGGTCGGTCTAAACTACACAGAAATCTTCGAGAAGCCCTTCCAACAAAC
AACGCTTCGAATGAAGCATGGAACATACGACAAGCTCGACGAGGATGGTATCGTGGCTCCTGGTGTGCGAGTGTCAGGTGAAGATATCATCAT

TGGCAAGACTGCACCTATCGACCAGGAGAATCAAGATCTCGGTACCAGAACTCAGTCACACCAGCGCCGTGATATCTCTACACCTCTGCGAAG
TACGGAGAACGGTATTGTTGATCAAGTCATCTTGACCGTCAACGCCGACAATGTCAAATACGTCAAGGTCCGAGTACGAACAACCAAGATTCC
CCAGATTGGTGACAAGTTTGCTTCTCGTCACGGTCAAAAGGGTACAATCGGTGTAACTTACCGACAGGAGGATATGCCCTTCAGCAGAGAGGG
TCTGACTCCCGATATTATTATCAACCCTCACGCCATTCCATCTCGAATGACAATTGCCCATTTGATTGAGTGTCTGCTAAGTAAAGTCTCAACAC
TTGAGGGTATGGAGGGTGATGCGACGCCCTTCACCGATGTCACCGTCGATTCCGTGTCGGAACTTCT
>13 JQ038008.1-*Fusarium euwallaceae*
TTGCTATCCCACATCGAATTCCCCGTCGAATTCCCTC-------CCTCACGATCCGCGCTGCGCCCGCTTCTCCCGAGTCCCAAAAATTTTGCGGTG-
CGACCGTGAATTTTTTT--GGTGGGGCATTTTTACCCCGCCACTCGGGCGACGTTGGACAAAGCCCTGATCCCTGCACACAAAAA-
CACCAAACCCTCT-TGGCGCGCATCAT-CACGTGATTGACCACAAACGCTAACCGACTC-
TACAACAGGAAGCCGCTGAGCTCGGTAAGGGTTCCTTCAAGTACGCCTGGGTCCTTGACAAGCTCAAGGCCGAGCGTGAGCGTGGTATCATGA
ACATACCTAAA-CGTTGCTTCGGCGGGAA-CAGACG----GCCCCGTAA-CACGGGCCGCC--CCCGCCAGAGGACCCCCTAAC-TC---TGTTTCT-
ATCATGTTTCTTCTGAGTAAA-
CAAGCAAATAAATTAAAACTTTCAACAACGGATCTCTTGGCTCTGGCATCGATGAAGAACGCAGCGAAATGCGATAAGTAATGTGAATTGCAG
AATTCAGTGAATCATCGAATCTTTGAACGCACATTGCGCCCGCCAGTATTCTGGCGGGCATGCCTGTTCGAGCGTCATTAC-
AACCCTCAGGCCCCCGGGCC-TGGCGTTGGGGATCGGCG--GAGC-
CCCCTGCGGGCACACGCCGTCCCCCAAATACAGTGGCGGTCCCGCCGCAGCTTCCATTGCGTAGTAGCTA-
ACACCTCGCAACTGGAGAGCGGCGCGGCCACGCCGTAAAACACCCCGGTCAGAACGCCATCGTCGCTATCGCTTGCTACTCTGGTTACAACCA
GGAAGATTCCGTCATTATGAACCAGAGTAGTATCGATCGAGGCTTGTTCCGCAGTCTGTTCTTCAGATCTTACTCCGATCAGGAGAAGAAGGTC
GGTCTGAACTACACGGAAGTGTTTGAGAAGCCCTTCCAGCAGTCGACACTTCGCATGAAGCACGGTACCTACGACAAGTTGGACGAGGATGGT
ATCGTGGCTCCAGGTGTGCGAGTGTCAGGTGAAGATATCATCATCGGCAAAACTGCGCCGATTGATCAGGAGAACCAGGATCTCGGTACCAGG
ACAACAGTGCACCAGCGTCGTGATATCTCCACGCCGCTGCGAAGCACTGAGAACGGTATCGTTGACTCGGTCATTGTGACTGTCAATGCCGAC
AACGTCAAGTATGTCAAGGTCCGTGTGAGAACGACCAAGATTCCCCAGATTGGTGACAAGTTCGCCTCTCGTCACGGACAGAAGGGTACCATT
GGTGTCACCTACAGACAGGAGGATATGCCATTCTCCAGGGAGGGTGTGACACCAGACATTATTATTAACCCCCACGCCATTCCGTCGCGAATG
ACAATTGCCCATTTGATTGAATGCCTCCTCAGTAAGGTGTCAACGCTGGAAGGCATGGAGGGTGATGCAACACCTTTCACCGATGTCACTGTCG
ACTCCGTTTCGGAGCTGCT
>14 AF160279.1-*Fusarium fujikuroi*
TC-CTT-TGCCCACCGATTT----------CC--CTTA------CGGTTCGAAACGTGCCTG--CTACCCCGCTCGAGACC-TAAAATTTTGCGATA-
TGACCGTAATTTTTTTT--GGTGGGGCAT-TT-ACCCCGCCACTCGAGTGATGGGCGCGTTTT-GCCCTTT-----------CCTGTCCACAACCTCA--
ATGAGCGCAATGT-CACGTG-TCAA--------ACTAAACATTC--
GACAATAGGAAGCCGCTGAGCTCGGTAAGGGTTCCTTCAAGTACGCCTGGGTTCTTGACAAGCTCAAGGCCGAGCGTGAGCGTGGTATCATGA
ACATACCAA-T-TGTTGCCTCGGCGG--ATCAGCCC--GCTCCCGGTAA-AACGGGACGG---CCCGCCAGAGGACCCCTAAAC-TC---TGTTTCT--
ATATGTAACTTCTGAGTAAAACCA-
TAAATAAATCAAAACTTTCAACAACGGATCTCTTGGTTCTGGCATCGATGAAGAACGCAGCAAAATGCGATAAGTAATGTGAATTGCAGAATT
CAGTGAATCATCGAATCTTTGAACGCACATTGCGCCCGCCAGTATTCTGGCGGGCATGCCTGTTCGAGCGTCATTTC-
AACCCTCAAGCCCCCGGGTT-TGGTGTTGGGGATCGG-----CGAGCCCTTGCGG---
CAAGCCGGCCCCGAAATCTAGTGGCGGTCTCGCTGCAGCTTCCATTGCGTAGTAGTAAAAC-
CCTCGCAACTGGTACGCGGCGCGGCCAAGCCGTTAAACC-
CCTGGTCAAAATGCCATTGTCGCAATTGCTTGCTACTCAGGTTATAACCAGGAAGATTCCGTCATTATGAACCAGAGTAGTATTGATCGAGGTC
TGTTCCGAAGTCTGTTCTTCCGATCGTACTCAGATCAAGAGAAGAAGGTCGGTCTCAACTACACTGAGATCTTCGAGAAGCCTTTCCAGCAGAC
AACACTCCGAATGAAGCATGGAACATACGACAAGCTTGATGAGGATGGTATCGTGGCGCCTGGTGTCCGTGTGTCAGGTGAGGATATCATTAT
CGGCAAGACTGCACCCATCGACCAAGAAAACCAAGACCTTGGCACAAGAACTCAATCGCACCAGCGTCGTGATATCTCGACACCACTGCGAA
GTACTGAGAACGGTATCGTTGATCAGGTCATTCTGACAGTCAACGCCGACAACGTCAAGTACGTCAAGGTTCGAGTACGAACCACCAAGATTC
CTCAAATTGGTGACAAGTTTGCTTCTCGTCACGGTCAAAAGGGTACAATTGGTGTTACATATCGACAGGAGGATATGCCTTTCAGCCGAGAAGG
TCTCACTCCCGATATCATTATCAATCCTCACGCCATTCCATCGCGAATGACAATTGCTCATTTGATTGAGTGTCTTCTTAGCAAGGTTTCAACGCT
GGAAGGTATGGAGGGTGATGCTACACCGTTCACTGATGTCACAGTCGATTCAGTCTCTGAACTTCT
>15 LT746230.1-*Fusarium globosum*
TC-CTC-TGCCCACCGATTT----------CC--CTTA------CGATTCGAAACGTGCCTG--CTACCCCGCTCGAGACC-AAAAATTTTGCGATG-
TGACCGTAATTTTTTT---GGTGGGGCAT-TT-ACCCCGCCACTCGAGCGATGGGCGCGTTTTTGCCCTTT-----------CCTGTCCACAACCTCA--
ATGAGCGCATTGT-CACGTG-TCAAG--CAGCCACTAACCATTC--
GACAATAGGAAGCCGCTGAGCTCGGTAAGGGTTCCTTCAAGTACGCCTGGGTTCTTGACAAGCTCAAGGCCGAGCGTGAGCGTGGTATCATGA
ACATACCAA-T-TGTTGCCTCGGCGG--ATCAGCCC--GCTCCCGGTAA-AACGGGACGG---CCCGCCAGAGGACCCCTAAAC-TC---TGTTTCT--
ATATGTAACTTCTGAGTAAAACCA-
TAAATAAATCAAAACTTTCAACAACGGATCTCTTGGTTCTGGCATCGATGAAGAACGCAGCAAAATGCGATAAGTAATGTGAATTGCAGAATT
CAGTGAATCATCGAATCTTTGAACGCACATTGCGCCCGCCAGTATTCTGGCGGGCATGCCTGTTCGAGCGTCATTTC-
AACCCTCAAGCCCCCGGGTT-TGGTGTTGGGGATCGG-----CGAGCCCTTGCGG---
CAAGCCGGCCCCGAAATCTAGTGGCGGTCTCGCTGCAGCTTCCATTGCGTAGTAGTAAAAC-
CCTCGCAACTGGTACGCGGCGCGGCCAAGCCGTTAAACC-
CCTGGCCAAAATGCCATTGTCGCAATTGCATGCTACTCAGGTTATAACCAGGAAGATTCCGTCATTATGAACCAGAGTAGTATCGATCGAGGTC
TGTTCCGAAGTCTGTTCTTCCGATCGTACTCAGATCAAGAGAAGAAGGTCGGTCTCAACTACACTGAGATCTTCGAGAAGCCTTTCCAGCAGAC
AACACTCCGAATGAAGCATGGAACATACGACAAGCTTGATGAGGATGGTATCGTGGCGCCTGGTGTCCGTGTGTCAGGTGAGGATATCATTAT
CGGCAAGACTGCACCCATCGACCAAGAAAACCAAGACCTTGGCACAAGAACTCAATCGCATCAGCGTCGTGATATCTCGACACCACTGCGAA
GTACTGAGAACGGTATCGTTGATCAGGTCATTCTGACAGTCAACGCCGACAACGTCAAGTACGTCAAGGTTCGAGTACGAACCACCAAGATTC
CTCAAATTGGTGACAAGTTTGCTTCTCGTCACGGTCAAAAGGGTACAATCGGTGTTACATATCGACAGGAGGATATGCCTTTCAGCCGAGAAGG
TCTCACTCCCGATATCATTATCAACCCTCACGCCATTCCATCGCGAATGACAATTGCCCATTTGATTGAGTGTCTTCTTAGCAAGGTTTCAACGC
TGGAAGGTATGGAGGGTGATGCTACACCGTTCACTGATGTCACAGTCGATTCAGTCTCTGAACTTCT
>17 AF160281.1-*Fusarium mangiferae*
TC-CTT-TGCCCGTCGATTT----------TC--CCTA------CGATTCGAAACGTGCCCG--CTACCCCGCTCGAGACC-AAAAATTTTGCGATA-
TGACCGTAATTTTTTTTT-GGTGGGGCAT-TT-ACCCCGCCACTCGAGCGATGGGCGCGT--TTGCCCTCT-----------CC-----ACAACCTCA--
ATGAGCGCATCGT-CACGTG-TCAAG--CAGTCACTAACCATCC--

GACAATAGGAAGCCGCTGAGCTCGGTAAGGGTTCCTTCAAGTACGCCTGGGTTCTTGACAAGCTCAAGGCCGAGCGTGAGCGTGGTATCATGA
ACATACCAA-T-TGTTGCCTCGGCGG--ATCAGCCC--GCTCCCGGTAA-AACGGGACGG---CCCGCCAGAGGACCC-TAAAC-TC---TGTTTCT--
ATATGTAACTTCTGAGTAAAACCA-
TAAATAAATCAAAACTTTCAACAACGGATCTCTTGGTTCTGGCATCGATGAAGAACGCAGCAAAATGCGATAAGTAATGTGAATTGCAGAATT
CAGTGAATCATCGAATCTTTGAACGCACATTGCGCCCGCCAGTATTCTGGCGGGCATGCCTGTTCGAGCGTCATTTC-
AACCCTCAAGCCCCCGGGTT-TGGTGTTGGGGATCGG-----CGAGCCCTTGCGG---
CAAGCCGGCCCCGAAATCTAGTGGCGGTCTCGCTGCAGCTTCCATTGCGTAGTAGTAAAAC-
CCTCGCAACTGGTACGCGGCGCGGCCAAGCCGTTAAACC-
CCTGGTCAAAATGCCATTGTCGCAATTGCTTGCTACTCAGGTTATAACCAGGAAGATTCCGTCATTATGAACCAGAGTAGTATCGATCGAGGTC
TGTTCCGAAGTCTGTTCTTCCGATCGTACTCAGATCAAGAGAAGAAGGTTGGTCTCAATTACACTGAGATCTTTGAGAAGCCTTTCCAGCAGAC
AACACTCCGAATGAAGCATGGAACGTACGACAAGCTTGATGAGGATGGTATCGTGGCGCCTGGTGTCCGTGTGTCAGGTGAAGATATCATTAT
TGGCAAGACTGCACCCATCGACCAAGAAAACCAAGACCTTGGCACAAGAACTCAATCGCACCAGCGTCGTGATATCTCGACACCACTGCGAA
GTACTGAGAACGGTATCGTTGATCAGGTCATTCTGACAGTCAACGCCGATAACGTCAAGTACGTCAAGGTTCGAGTACGAACCACCAAGATTC
CTCAAATTGGTGACAAGTTTGCTTCTCGTCACGGTCAAAAGGGTACAATCGGTGTTACATATCGACAGGAGGATATGCCTTTCAGCCGAGAAGG
TCTCACTCCCGATATCATTATCAACCCTCACGCCATTCCATCGCGAATGACAATTGCCCATTTGATTGAGTGTCTTCTTAGCAAGGTTTCAACAC
TGGAAGGTATGGAGGGTGATGCTACACCGTTCACTGATGTCACAGTCGATTCAGTCTCTGAACTTCT
>18 AF160266.1-*Fusarium napiforme*
TC-CTT-TGTACACCGATTC----------CT-CCCTA------CGACTCGAAACGTGCCCA--CTACCCCGCTTGAGTTC-
AAAAATTTTGCGATAATGACCGTAATTTTTTT---GGTGGGGCAT-TT-ACCCCGCCACTCGAGCGGCG----CGTTTCTGCCCTCT-C---------
TCATTCCACAACCTCA--CTGAGCGCATCGT-CACGTG-TCAAG--CAGTCACTAACCATCC--
GACAATAGGAAGCCGCTGAGCTCGGTAAGGGTTCCTTCAAGTACGCCTGGGTTCTTGACAAGCTCAAGGCCGAGCGTGAGCGTGGTATCATGA
ACATACCAA-T-TGTTGCCTCGGCGG--ATCAGCCC--GCTCCCGGTAA-AACGGGACGG---CCCGCCAGAGGACCCCTAAAC-TC---TGTTTCT--
ATATGTAACTTCTGAGTAAAACCA-
TAAATAAATCAAAACTTTCAACAACGGATCTCTTGGTTCTGGCATCGATGAAGAACGCAGCAAAATGCGATAAGTAATGTGAATTGCAGAATT
CAGTGAATCATCGAATCTTTGAACGCACATTGCGCCCGCCAGTATTCTGGCGGGCATGCCTGTTCGAGCGTCATTTC-AACCCTCAAGCCC---
AGCT-TGGTGTTGGGACTCG------CGAGTCAAATCG---------CGTTCCCCAAATTGATTGGCGGTCACGTCG-
AGCTTCCATAGCGTAGTAGTAAAAC-CCTCGTTACTGGTAATCGTCGCGGCCACGCCGTTAAACC-
CCTGGTCAAAATGCCATTGTCGCAATTGCTTGCTACTCAGGTTACAACCAGGAAGATTCCGTCATTATGAACCAGAGTAGTATTGATCGAGGTC
TGTTCCGAAGCCTGTTCTTCCGATCATACTCAGATCAAGAGAAGAAGGTCGGCCTTAACTACACTGAGATCTTTGAGAAGCCTTTCCAGCAGAC
AACACTTCGCATGAAGCATGGAACATACGACAAGCTTGATGAGGACGGTATCGTCGCGCCCGGTGTCCGTGTGTCAGGTGAAGATATCATTAT
CGGCAAGACTGCACCCATCGACCAAGAAAACCAGGACCTTGGCACAAGAACTCAGTCGCATCAGCGTCGTGATATCTCGACACCACTGCGAA
GTACTGAGAACGGTATCGTTGATCAAGTCATTCTGACAGTCAACGCCGATAACGTCAAGTACGTTAAGGTCCGAGTACGAACCACCAAGATTC
CTCAAATTGGTGACAAGTTTGCTTCTCGTCACGGCCAGAAGGGTACAATTGGTGTTACATATCGACAGGAGGATATGCCTTTCAGCCGAGAAGG
TCTCACTCCTGATATCATTATCAACCCTCACGCCATTCCGTCGCGAATGACAATTGCCCATTTGATTGAGTGTCTTCTTAGCAAGGTTTCGACGCT
GGAAGGTATGGAGGGTGACGCTACACCATTCACTGATGTCACAGTCGATTCAGTCTCAGAACTTCT
>19 GQ505402.1-*Fusarium nelsonii*
CC-CCCATACCCATCGATACATCATTCGAATCGCTCTTTCACGACGACTCGACAAGCGTCCG--TTACCCCGCTCGAGCTC-
AAAAATTTTGCGGTT-CTGTCGTAATTTTTTTCT-GGTGGGGCCT-CT-ACCCCGCCACTCGAGTGACGGGCGCTTACCCTCTTCCCAC---------
AAAAACCATCATTCATTTGGGCGCGCATCAT-CACGTG-TGTAT--CAGTCACTAACCATCT--
GACAATAGGAAGCCGCCGAGCTCGGTAAGGGTTCCTTCAAGTACGCCTGGGTTCTTGACAAGCTCAAGGCCGAGCGTGAGCGTGGTATCATGA
ACATACCTT-AACGTTGCCTCGGCGG--ATCAGCCC--GCGCCCCGTAA-AACGGGACGG---CCCGCCGCAGGAC--CCATAA-ACCC-TGAATTT--
TATTGTAACTTCTGAGTTTAAAAAACAAATAAATCAAAACTTTCAACAACGGATCTCTTGGTTCTGGCATCGATGAAGAACGCAGCAAAATGC
GATAAGTAATGTGAATTGCAGAATTCAGTGAATCATCGAATCTTTGAACGCACATTGCGCCCGCCAGTATTCTGGCGGGCATGCCTGTTCGAGC
GTCATTTC-AACCCTCAAGCCCCCGGGTT-TGGTGTTGGGGATCGGG---CTGCGACTTTCCGC---AGC-
CCGGCCCCGAAATCTAGTGGCGGTCTCGCTGCAGCCTCCATTGCGTAGTAGCTA-
ACACCTCGCAACTGGAACGCGGCGCGGCCAAGCCGTTAAACC-
CCCGGTCAAAACGCCATCGTCGCTATTGCTTGTTACTCAGGATACAACCAGGAAGATTCCGTCATTATGAACCAGAGCAGTATCGATCGAGGTC
TATTCCGAAGTTTGTTCTTCCGATCATACTCGGACCAGGAGAAGAAGGTCGGTCTCAACTACACAGAAATCTTTGAGAAGCCCTTCCAACAAAC
AACGCTTCGAATGAAGCATGGAACTTACGACAAGCTTGACGAGGACGGCATTGTCGCCCCTGGTGTACGTGTGTCAGGTGAGGATATCATTATT
GGCAAGACTGCACCTATCGACCAAGAGAACCAAGATCTTGGTACCAGAACCCAGACGCATCAGCGCCGTGATATTTCGACGCCTCTGCGAAGT
ACTGAGAACGGTATTGTTGATCAAGTCATCTTGACCGTCAACGCCGACAACGTCAAATACGTCAAGGTTCGAGTACGAACAACCAAGATCCCC
CAGATTGGTGACAAGTTTGCTTCTCGTCACGGTCAAAAGGGTACAATTGGTGTCACTTATCGACAGGAGGATATGCCCTTCAGCAGAGAGGGG
TTGACCCCCGACATTATCATCAACCCTCACGCTATTCCATCTCGTATGACAATTGCCCATTTGATTGAGTGTCTTCTAAGTAAGGTCTCAACACTT
GAGGGTATGGAGGGTGACGCCACACCATTCACTGATGTTACCGTCGATTCCGTCTCGGAACTCTT
>20 AF160273.1-*Fusarium nygamai*
TC-CTT-TGTCCATCGATTT----------CC-CCCTC------CGACTCGAAACGTGCCCG--CTACCCCGCTCGAATTC-AAAAATTTTGCGATA-
TGACCGTAATTTTTTT---GGTGGGGCAT-TT-ACCCCGCCACTCGAGCGGCG----CGTTTTTGCCCTCTTC---------CCATTCCACAACCTCA--
CTGAGCGCATCGT-CACGTG-TCAAG--CAGTCACTAACCATTC--
GACAATAGGAAGCCGCTGAGCTCGGTAAGGGTTCCTTCAAGTACGCCTGGGTTCTTGACAAGCTCAAGGCCGAGCGTGAGCGTGGTATCATGA
ACATACCAA-T-TGTTGCCTCGGCGG--ATCAGCCC--GCTCCCGGTAA-AACGGAACGG---CCCGCCAGAGGACCCCTAAAC-TC---TGTTTCT--
ATATGTAACTTCTGAGTAAAACCA-
TAAATAAATCAAAACTTTCAACAACGGATCTCTTGGTTCTGGCATCGATGAAGAACGCAGCAAAATGCGATAAGTAATGTGAATTGCAGAATT
CAGTGAATCATCGAATCTTTGAACGCACATTGCGCCCGCCAGTATTCTGGCGGGCATGCCTGTTCGAGCGTCATTTC-
AACCCTCAAGCCCCCGGGTT-TGGTGTTGGGGATCGG-----CGAGCC-TCACGG---
CAAGCCGGCCCCGAAATACAGTGGCGGTCTCGCTGCAGCTTCCATTGCGTAGTAGTAAAAC-
CCTCGCAACTGGTACGCGGCGCGGCCAAGCCGTTAAACC-
CCCGGTCAAAATGCCATTGTCGCCATTGCTTGCTACTCAGGTTATAACCAGGAAGATTCCGTCATTATGAACCAGAGTAGTATTGATCGAGGTC
TGTTCCGAAGTCTGTTCTTCCGATCGTACTCAGATCAAGAGAAGAAGGTCGGTCTCAACTACACTGAAATCTTTGAGAAGCCTTTCCAGCAGAC
AACACTTCGCATGAAGCATGGAACATACGACAAGCTTGATGAAGATGGTATCGTGGCTCCTGGTGTCCGTGTATCAGGTGAAGATATCATTATC

GGCAAGACTGCACCCATCGATCAAGAGAACCAGGACCTTGGCACAAGGACTCAATCGCACCAACGTCGTGATATCTCGACACCACTGCGAAG
TACCGAGAACGGTATCGTTGATCAAGTCATTCTGACAGTCAACGCCGATAACGTCAAGTACGTCAAGGTTCGAGTGCGAACCACCAAGATTCC
TCAAATTGGTGACAAGTTTGCTTCTCGTCACGGTCAAAAGGGTACAATCGGTGTTACATATCGACAGGAGGATATGCCTTTCAGCCGAGAAGGT
CTCACTCCCGACATCATTATCAACCCTCACGCCATTCCATCGCGAATGACAATTGCCCATTTGATTGAGTGTCTTCTTAGCAAGGTTTCAACACT
GGAAGGTATGGAGGGTGACGCTACGCCGTTCACTGATGTCACAGTCGATTCAGTCTCAGAACTTCT
>22 AF160274.1-*Fusarium phyllophilum*
TC--TT-TGTCCATCGATTT----------CC--CCTA------CGACTCGAAACGCGCCCA--CTACCCCGCTCGAGACC-AAAAATTTTGCGATA-
TGACCGTAATTTTTTTT--GGTGGGGCAT-CT-ACCCCGCCACTCGAGCGATGGGCTCGTTTCTGCCCTCTCC---------CATCCCCACAACCTCA--
CTGAGCGCATCGT-CACGTG-TTGAA--CAGTCACTAACCATCT--
GACAAAAGGAAGCCGCTGAGCTCGGTAAGGGTTCCTTCAAGTACGCCTGGGTTCTTGACAAGCTCAAGGCCGAGCGTGAGCGTGGTATCATGA
ACATACCAA-T-TGTTGCCTCGGCGG--ATCAGCCC--GCTCCCGGTAA-AACGGGACGG---CCCGCCAGAGGACCCCTAAAC-TC---TGTTTCT--
ATATGTAACTTCTGAGTAAAACCA-
TAAATAAATCAAAACTTTCAACAACGGATCTCTTGGTTCTGGCATCGATGAAGAACGCAGCAAAATGCGATAAGTAATGTGAATTGCAGAATT
CAGTGAATCATCGAATCTTTGAACGCACATTGCGCCCGCCAGTATTCTGGCGGGCATGCCTGTTCGAGCGTCATTTC-
AACCCTCAAGCCCTCGGGTT-TGGTGTTGGGGATCGG-----CGAGCCCTTGCGG---
CAAGCCGGCCCCGAAATATAGTGGCGGTCTCGCTGCAGCTTCCATTGCGTAGTAGTAAAAC-
CCTCGCAACTGGTACGCGGCGCGGCCAAGCCGTTAAACC-
CCTGGTCAAAATGCCATTGTCGCAATTGCTTGCTACTCAGGTTATAACCAGGAAGATTCCGTCATTATGAACCAGAGTAGTATTGATCGAGGTC
TTTTCCGAAGTTTGTTCTTCCGATCGTACTCAGATCAAGAGAAGAAGGTCGGTCTCAACTACACTGAGATATTTGAGAAGCCTTTCCAGCAGAC
AACACTCCGAATGAAGCATGGAACATACGACAAGCTTGATGAGGATGGTATCGTGGCACCTGGTGTCCGTGTGTCAGGTGAAGATATCATTAT
CGGCAAGACTGCACCCATCGACCAAGAGAACCAGGACCTTGGCACAAGGACTCAATCGCATCAGCGCCGTGATATCTCGACACCACTGCGAA
GTACTGAGAATGGTATCGTTGATCAAGTCATTCTGACAGTCAACGCCGATAACGTCAAGTACGTCAAGGTCCGAGTACGAACCACCAAGATTC
CTCAAATTGGTGACAAGTTTGCTTCTCGTCACGGTCAAAAGGGTACAATCGGTGTTACATATCGACAGGAGGATATGCCTTTCAGCCGAGAAGG
TCTCACTCCCGATATCATCATCAACCCTCACGCCATTCCATCGCGAATGACAATTGCCCATTTGATTGAGTGTCTTCTTAGCAAGGTTTCAACGC
TGGAAGGTATGGAGGGTGATGCTACACCGTTCACTGATGTCACTGTCGATTCAGTCTCAGAACTTCT
>23 AF160267.1-*Fusarium ramigenum*
TC-CTT-TGTCCACCGATTT----------CTTCCCTA------CGACTCGAAACGTGCCCA--CTACCCCGCTCGAGTTC-AAAAATTTTGCGATA-
TGACCGTAATTTTTTT---GGTGGGGCAT-TT-ACCCCGCCACTCGAGTGGCG----CGTTTCTGCCCTCT-C---------TCAT--CACAACCTCA--
CTGAGCGCATCGT-CACGTG-TCAAG--CAGTCACTAACCATCT--
GACAATAGGAAGCCGCTGAGCTCGGTAAGGGTTCCTTCAAGTACGCCTGGGTTCTTGACAAGCTCAAGGCCGAGCGTGAGCGTGGTATCATGA
ACATACCAA-T-TGTTGCCTCGGCGG--ATCAGCCC--GCTCCCGGTAA-AACGGGACGG---CCCGCCAGAGGACCCACAAAC-TC---TGTTTCT--
ATATGTAACTTCTGAGTAAAACCA-
TAAATAAATCAAAACTTTCAACAACGGATCTCTTGGTTCTGGCATCGATGAAGAACGCAGCAAAATGCGATAAGTAATGTGAATTGCAGAATT
CAGTGAATCATCGAATCTTTGAACGCACATTGCGCCCGCCAGTATTCTGGCGGGCATGCCTGTTCGAGCGTCATTTC-AACCCTCAAGCCC---
AGCT-TGGTGTTGGGACTCG------CGAGTCAAATCG---------CGTTCCCCAAATTGATTGGCGGTCACGTCG-
AGCTTCCATAGCGTAGTAGTAAAAC-CCTCGTTACTGGTAATCGTCGCGGCCACGCCGTTAAACC-
CCTGGTCAAAATGCCATTGTCGCAATTGCTTGCTACTCAGGTTATAACCAGGAAGATTCCGTCATTATGAACCAGAGTAGTATTGATCGAGGTC
TGTTCCGAAGTCTATTCTTCCGATCGTACTCAGATCAAGAGAAGAAGGTCGGTCTTAACTACACTGAAATCTTTGAGAAGCCTTTCCAGCAGAC
AACACTTCGCATGAAGCATGGAACATTCGACAAGCTTGATGAGGATGGTATCGTGGCTCCTGGTGTCCGTGTATCAGGTGAAGATATCATTATC
GGCAAGACTGCACCCATCGACCAAGAAAACCAGGACCTTGGCACAAGAACTCAGTCGCATCAGCGTCGTGATATCTCGACACCACTGCGAAG
TACTGAGAACGGTATCGTTGATCAAGTCATTCTGACAGTCAACGCCGACAACGTCAAGTACGTCAAGGTTCGAGTGCGAACCACCAAGATTCC
TCAGATTGGTGACAAGTTTGCTTCTCGTCACGGTCAAAAGGGTACAATTGGTGTTACATATCGACAGGAGGATATGCCTTTCAGCCGAGAAGGT
CTCACTCCTGATATCATTATCAACCCTCACGCCATTCCGTCGCGAATGACAATTGCCCATTTGATTGAGTGTCTTCTTAGCAAGGTTTCGACGCT
GGAAGGTATGGAGGGTGACGCTACACCATTCACTGATGTCACAGTCGATTCAGTCTCAGAACTTCT
>24 AF160278.1-*Fusarium sacchari*
TC-CTT-TGTACATCGATTT----------CC--CCTA------CGACTCGAAACGTGCCCG--CTACCCCGCTCGAGACC-AAAAATTTTGCGATA-
TGACCGTAATTTTTTT---GGTGGGGCAT-TT-ACCCCGCCACTCGAGCGATGGGCGCGTCTTTGCCCTTT-----------CCTATCCACAACTTCA--
ATGAGCGCATCGT-CACGTG-TCAAG--CAGTCACTAATCATCT--
GACAATAGGAAGCCGCTGAGCTCGGTAAGGGTTCCTTCAAGTACGCCTGGGTTCTTGACAAGCTCAAGGCCGAGCGTGAGCGTGGTATCATGA
ACATACCGA-T-TGTTGCCTCGGCGG--ATCAGCCC--GCTCCCGGTAA-AACGGGACGG---CCCGCCAGAGGACCCCCAAAC-TC---TGTTTCT--
ATATGTAACTTCTGAGTAAAACCA-
TAAATAAATCAAAACTTTCAACAACGGATCTCTTGGTTCTGGCATCGATGAAGAACGCAGCAAAATGCGATAAGTAATGTGAATTGCAGAATT
CAGTGAATCATCGAATCTTTGAACGCACATTGCGCCCGCCAGTATTCTGGCGGGCATGCCTGTTCGAGCGTCATTTC-AACCCTCAAGCCC---
AGCT-TGGTGTTGGGACTCG------CGAGTCAAATC----------CGTTCCCCAAATTGATTGGCGGTCACGTCG-AGCTTCCATAGCGTAGTAGTAAAAC-
CCTCGCAACTGGTAATCGTCGCGGCCACGCCGTTAAACC-
CCCGGTCAAAACGCCATTGTTGCAATTGCTTGCTACTCAGGTTACAACCAGGAAGATTCCGTCATCATGAACCAGAGCAGTATCGATCGAGGTC
TGTTCCGAAGTCTGTTCTTCCGATCGTACTCAGATCAAGAAAAGAAGGTCGGTCTCAACTACACTGAGATCTTCGAGAAGCCTTTCCAGCAGAC
AACACTTCGCATGAAGCATGGAACGTACGACAAGCTTGATGAGGATGGTATCGTGGCTCCTGGTGTTCGTGTATCAGGTGAAGATATCATTATC
GGCAAGACTGCACCCATCGACCAAGAAAACCAAGACCTTGGCACAAGAACTCAATCGCACCAGCGTCGTGATATCTCGACACCATTGCGAAG
TACTGAGAACGGTATCGTTGATCAAGTCATTCTGACAGTCAACGCCGACAACGTCAAGTACGTCAAGGTTCGAGTACGAACCACCAAGATTCC
TCAAATTGGTGACAAGTTTGCTTCTCGTCACGGTCAAAAGGGTACCATCGGTGTTACGTATCGACAGGAGGATATGCCTTTCAGCCGAGAAGGT
CTCACTCCCGATATCATTATCAACCCTCACGCCATTCCATCGCGAATGACAATTGCCCATTTGATTGAGTGTCTTCTTAGCAAGGTTTCAACACT
GGAGGGTATGGAGGGTGACGCTACACCGTTTACTGATGTCACAGTCGATTCAGTTTCTGAACTTTT
>25 GQ505592.1-*Fusarium scirpi*
CC-CTC-TGCCCATCGATCCATCACTCGAATCAGTCT----CG---ACTGAATATGCGCCTG--TTACCCCGCTCGAGTAC-AAAA-TTTTGCGGTT-
CAATCGTAATTTTTT----GGTGGGGCCT-AT-ACCCCGCTACTCGAGTGACAGGCGCTTGCCCTCTTCCCAC---------AAAA----TCACCTATT---
GCGCA-----T-CACGTG-TCAAT--CAGTCACTGACCACCC--
GATAATAGGAAGCCGCCGAGCTCGGTAAGGGTTCCTTCAAGTACGCTTGGGTTCTTGACAAGCTCAAGGCCGAGCGTGAGCGTGGTATCATGA
ACATACCTA-TACGTTGCCTCGGCGG--ATCAGCCC--GCGCCCTGTAA-AAAGGGACGG---CCCGCCCGAGGACCC-TAAAC-TC---TGTTTTT--A-

GTGGAACTTCTGAGTAAAACAAACAAATAAATCAAAACTTTCAACAACGGATCTCTTGGTTCTGGCATCGATGAAGAACGCAGCAAAATGCG
ATAAGTAATGTGAATTGCAGAATTCAGTGAATCATCGAATCTTTGAACGCACATTGCGCCCGCCAGTATTCTGGCGGGCATGCCTGTTCGAGCG
TCATTTC-AACCCTCAAGCTC---AGCT-TGGTGTTGGGACTCG------CG--GTAACCCG---------CGTTCCCCAAATCGATTGGCGGTCACGTCG-
AGCTTCCATAGCGTAGTAATCATACACCTCGTTACTGGTAATCGTCGCGGCCACGCCGTAAAACC-
CCCGGTCAAAACGCCATTGTTGCTATTGCTTGTTATTCAGGATACAACCAGGAAGATTCCGTCATTATGAACCAGAGCAGTATTGATCGAGGCT
TGTTCCGCAGTCTCTTCTTCCGATCATACTCGGATCAGGAGAAGAAGGTCGGTCTAAACTACACAGAAATCTTCGAGAAGCCCTTCCAACAAAC
AACGCTTCGAATGAAGCATGGAACATACGACAAGCTCGACGAGGATGGCATCGTGGCTCCTGGTGTGCGAGTGTCAGGTGAAGATATCATCAT
TGGCAAGACTGCACCTATCGACCAGGAGAATCAAGATCTCGGTACCAGAACTCAGTCGCACCAGCGCCGTGATATCTCTACACCTCTGCGAAG
TACGGAGAACGGTATTGTTGATCAAGTCATCTTGACCGTCAACGCCGACAATGTCAAATACGTCAAGGTCCGAGTACGAACAACCAAGATTCC
CCAGATTGGTGACAAGTTTGCTTCTCGTCACGGTCAAAAGGGTACAATCGGTGTAACTTACCGGCAGGAGGACATGCCCTTCAGCAGAGAGGG
TCTGACTCCCGATATTATTATCAACCCTCACGCCATTCCATCTCGAATGACAATTGCCCATTTGATTGAGTGTCTGCTAAGTAAAGTCTCAACAC
TTGAGGGTATGGAGGGTGACGCGACGCCTTTCACCGATGTCACCGTCGATTCCGTGTCGGAACTTCT
>27 AF160270.1-*Fusarium thapsinum*
TC-CTT-TGTCCATCGATTA----------CC--CCTA------CGACTCGAAACTTGCCCG--CTACCCCGCTCGAGTT--AAAAATTTTGCGATA-
TGACCATAATTTTTTC---GGTGGGGCAT-TT-ACCCCGCCACTCGAGCGATG----GGC----GCCTTTTA---------CCCTCACACAACCTCAA-
CTGAGCGCATTGT-CACGTG-TCAAG--CAGTCACTAACCATCC--
GACAATAGGAAGCCGCTGAGCTCGGTAAGGGTTCCTTCAAGTACGCCTGGGTTCTTGACAAGCTCAAGGCCGAGCGTGAGCGTGGTATCATGA
ACATACCAA-T-TGTTGCCTCGGCGG--ATCAGCCC--GCTCCCGGTAA-AACGGGACGG---CCCGCCAGAGGACCCCTAAAC-TC---TGTTTCT--
ATATGTAACTTCTGAGTAAAACCA-
TAAATAAATCAAAACTTTCAACAACGGATCTCTTGGTTCTGGCATCGATGAAGAACGCAGCAAAATGCGATAAGTAATGTGAATTGCAGAATT
CAGTGAATCATCGAATCTTTGAACGCACATTGCGCCCGCCAGTATTCTGGCGGGCATGCCTGTTCGAGCGTCATTTC-
AACCCTCAAGCCCCCGGGTT-TGGTGTTGGGGATCGG-----CGAGCC-TCACGG---CAAGCCGGCCCCGAAATACAGTGGCGGTCTCGCTG-
AGCTTCCATTGCGTAGTAGTAAAAC-CCTCGCAACTGGTACGCGGCGCGGCCAAGCCGTTAAACC-
CCTGGTCAAAATGCCATTGTCGCAATTGCTTGCTACTCAGGTTATAACCAGGAAGATTCCGTTATTATGAACCAGAGTAGTATCGATCGAGGTC
TGTTCCGAAGTCTGTTCTTCCGATCGTACTCAGATCAAGAGAAGAAGGTTGGCCTCAACTACACTGAGATCTTTGAGAAGCCTTTCCAGCAGAC
AACACTTCGAATGAAGCATGGAACATATGACAAACTTGACGAGGATGGTATTGTGGCGCCTGGTGTCCGTGTGTCAGGTGAGGATATCATTATC
GGCAAGACTGCACCCATCGACCAAGAAAACCAGGACCTTGGCACAAGAACTCAATCGCACCAGCGTCGTGATATCTCGACACCGCTGCGAAG
TACTGAGAACGGTATCGTTGATCAAGTCATTCTGACAGTCAACGCGGATAACGTCAAGTACGTTAAGGTTCGAGTGCGAACCACCAAGATTCC
TCAAATTGGTGACAAGTTCGCTTCTCGTCACGGTCAAAAGGGTACTATTGGTGTTACATATCGACAGGAGGATATGCCTTTCAGCCGAGAAGGT
CTCACTCCCGATATCATTATTAATCCTCATGCCATTCCATCGCGAATGACAATTGCCCATTTGATTGAGTGTCTTCTTAGCAAGGTTTCGACGCTG
GAAGGTATGGAGGGTGACGCTACACCATTCACTGATGTCACAGTTGATTCAGTCTCGGAACTTCT
>28 HM068307.1-*Fusarium tricinctum*
CG-CT----CCCATCGATT---CCCACGATTCGCTCCC------TCACTCGAAACGCATCCA--TTACCCCGCTCGAGCCC-GAGAATTTTGCGGTG-
CGACCGTGATTTTTTTCT-GGTGGGGTATCTT-ACCCCGCCACTCGAGTGACGGATGCGCTTGCCCTGTTCCC-----ACAAAAC-CTTACCACCCT----
GTCGCGCACTA--CATGT---CTTG--TAGTCACTAACCACTG--
GACAATAGGAAGCCGCCGAGCTCGGAAAGGGTTCCTTCAAGTACGCCTGGGTTCTTGACAAGCTCAAAGCCGAGCGTGAGCGTGGTATCATGA
ACATACCTT-AATGTTGCCTCGGCGG--ATCAGCCC--GCGCCCCGTAA-AACGGGACGG---CCCGCCAGAGGAC--CCAAAC-TCTAATGTTTCT--
TATTGTAACTTCTGAGTAAAACAAACAAATAAATCAAAACTTTCAACAACGGATCTCTTGGTTCTGGCATCGATGAAGAACGCAGCAAAATGC
GATAAGTAATGTGAATTGCAGAATTCAGTGAATCATCGAATCTTTGAACGCACATTGCGCCCGCTGGTATTCCGGCGGGCATGCCTGTTCGAGC
GTCATTTC-AACCCTCAAGCCCCCGGGTT-TGGTGTTGGGGATCGGCT--CTGCCCTTCTGGGC---
GGTGCCGCCCCCGAAATACATTGGCGGTCTCGCTGCAGCCTCCATTGCGTAGTAGCTA-
ACACCTCGCAACTGGAACGCGGCGCGGCCATGCCGTAAAACC-
CCTGGTCAAAACGCCATCGTCGCAATTGCCTGTTACTCAGGATACAACCAGGAAGATTCCGTTATTATGAACCAGAGCAGTATTGATAGAGGTC
TATTCCGCAGTCTGTTCTTCCGATCGTACTCAGATCAGGAGAAGAAGGTCGGTCTCAACTACACAGAAATCTTTGAGAAGCCTTTCCAGCAGAC
AACACTTCGAATGAAGCACGGAACATACGACAAGCTTGACGAAGATGGTATCGTGGCCCCTGGTGTACGAGTTTCCGGTGAAGATATCATTAT
TGGCAAGACGGCGCCCATCGATCAGGAGAACCAGGATCTGGGCACCAGAACCCAATCGCACCAGCGTCGCGATATCTCGACACCGCTGCGAA
GTACAGAAAACGGTATCGTTGATCAAGTCATTTTGACAGTCAACGCCGACAACGTGAAGTACGTCAAGGTTCGTGTACGAACCACCAAGATTC
CCCAGATTGGTGATAAGTTTGCTTCTCGTCACGGTCAAAAGGGTACCATTGGTGTCACTTATCGACAGGAGGATATGCCTTTCAGCAGAGAAGG
CCTGACTCCCGATATCATTATCAACCCTCACGCTATTCCCTCTCGAATGACAATTGCCCATTTGATTGAGTGTCTTCTTAGTAAGGTCTCAACACT
TGAAGGTATGGAGGGTGATGCAACGCCATTCACTGATGTGACAGTCGACTCTGTCTCAGAGCTTCT
>29 ExFu1A-OR891725
TTGCTATCCCACATCGAATTCCCCGTCGAATTCCCTC-------CTCCGCGACACGCTCTGCGCCCGCTTCTCCCGAGTCCCAAAAATTTTGCGGTT-
CGACCGTAATTTTTTTT--GGTGGGGCATTT--ACCCCGCCACTCGGGCGACGTTGGACAAAGCCCTGATCCCTGCACACAAAAA-
CACCAAACCCTCT-TGGCGCGCATCA----CGTGGTTCACAACAGACACTGACTGGTTC-
AACAATAGGAAGCCGCTGAGCTCGGTAAGGGTTCCTTCAAGTACGCCTGGGTCCTTGACAAGCTCAAGGCCGAGCGTGAGCGTGGTATCATGA
ACATACCTAAA-CGTTGCTTCGGCGGGAA-CAGACG----GCCCCGTGA-AACGGGCCGCC--CCCGCCAGAGGACCCC-TAAC-TC---TGTTTCT-
ATAATGTTTCTTCTGAGTAAAACAAGCAAATAAATTAAAACTTTCAACAACGGATCTCTTGGCTCTGGCATCGATGAAGAACGCAGCGAAATG
CGATAAGTAATGTGAATTGCAGAATTCAGTGAATCATCGAATCTTTGAACGCACATTGCGCCCGCCAGTATTCTGGCGGGCATGCCTGTTCGAG
CGTCATTAC-AACCCTCAGGCCCCCGGGCC-TGGCGTTGGGGATCGGCG-GA-GCCCCC-
CGTGGGCACACGCCGTCCCCCAAATACAGTGGCGGTCCCGCCGCAGCTTCCATCGCGTAGTAGCTA-
ACACCTCGCGACTGGAGAGCGGCGCGGCCACGCCGTAAAACACCCCGGTCAGAACGCCATCGTCGCTATCGCTTGCTACTCTGGTTATAACCA
GGAAGATTCCGTCATTATGAACCAGAGTAGTATCGATCGAGGCCTGTTCCGCAGTCTGTTCTTCCGATCCTACTCTGACCAGGAGAAGAAGGTC
GGCCTCAACTACACGGAAGTGTTTGAGAAGCCCTTCCAGCAGTCGACGCTTCGTATGAAGCACGGTACCTACGACAAGCTGGACGAGGATGGT
ATCGTGGCCCCCGGTGTGCGAGTGTCAGGTGAAGATATCATCATCGGCAAGACCGCGCCGATTGATCAAGAGAACCAGGATCTGGGTACCAG
GACAACGGTGCACCAGCGTCGTGATATCTCCACGCCGCTGCGAAGTACCGAGAACGGTATCGTCGATTCGGTCATCGTGACTGTCAATGCCGA
CAACGTCAAGTACGTCAAGGTCCGTGTGAGGACGACCAAGATTCCTCAGATTGGTGACAAGTTCGCCTCTCGTCACGGACAGAAGGGTACCAT
TGGTGTTACCTACCGACAGGAGGACATGCCCTTTAGCAGGGAGGGCGTGACACCAGACATTATCATTAACCCCCACGCCATTCCGTCGCGAAT
GACAATTGCCCATTTGATTGAATGCCTCCTCAGTAAGGTGTCAACGCTCGAAGGCATGGAGGGTGATGCCACACCTTTCACCGACGTCACTGTC
GACTCCGTTTCGGAGCTGCT

>30 ExFu2B-OR891726
TTGATATTCCACATCGAATTCCCCGTCGAATTCCCTC-------CATCGCGATACGCTCTGCGCCCGCTTCTCCCGAGTCCCAAAATTTTTGCGGTC-
CGACCGTAATTTTTTT---GGTGGGGCATTT--ACCCCGCCACTCGGGCGACGTTGGACAAAGCCCTGATCCCTGCACACAAAAA-
CACCAAACCCTCT-TGGCGCGCATCAT-CACGTGGTTCACGACAGACGCTAACCGGTCC-
AACAATAGGAAGCCGCTGAGCTCGGTAAGGGTTCCTTCAAGTACGCCTGGGTCCTTGACAAGCTCAAGGCCGAGCGTGAGCGTGGTATCATGA
ACATACCTAAAACGTTGCTTCGGCGGGAA-CAGACG----GCCCTGTAACAACGGGCCGCC--CCCGCCAGAGGACCCC-TAAC-TC---TGTTTTT-
ATAATGTTT-TTCTGAGTAAA-
CAAGCAAATAAATTAAAACTTTCAACAACGGATCTCTTGGCTCTGGCATCGATGAAGAACGCAGCGAAATGCGATAAGTAATGTGAATTGCAG
AATTCAGTGAATCATCGAATCTTTGAACGCACATTGCGCCCGCCAGTATTCTGGCGGGCATGCCTGTTCGAGCGTCATTAC-
AACCCTCAGGCCCCCGGGCC-TGGCGTTGGGGATCGGCA-GAAGCCCCC-
TGTGGGCACACGCCGTCCCTCAAATACAGTGGCGGTCCCGCCGCAGCTTCCATTGCGTAGTAGCTA-
ACACCTCGCAACTGGAGAGCGGCGCGGCCATGCCGTAAAACACCCCGGTCAGAACGCCATCGTCGCTATCGCTTGCTACTCTGGTTACAACCA
GGAAGATTCCGTCATTATGAACCAGAGTAGTATCGATCGAGGCCTGTTCCGCAGTCTGTTCTTCAGATCCTACTCTGACCAGGAGAAGAAGGTC
GGTCTGAACTACACGGAAGTATTCGAGAAGCCCTTCCAGCAGTCGACGCTTCGTATGAAGCATGGTACCTACGACAAGCTGGACGAGGATGGT
ATCGTGGCCCCTGGTGTGCGAGTGTCAGGTGAAGATATCATCATCGGCAAGACTGCGCCGATTGATCAAGAGAACCAGGATCTGGGTACCAGG
ACAACGGTGCACCAGCGTCGTGATATCTCCACGCCGCTGCGAAGTACCGAGAACGGTATCGTCGATTCGGTCATTGTGACTGTCAATGCCGAC
AACGTCAAGTACGTCAAGGTCCGTGTGAGGACGACCAAGATTCCCCAGATTGGTGACAAGTTCGCCTCTCGTCACGGACAGAAGGGTACCATT
GGTGTTACCTACCGACAGGAGGATATGCCCTTCACCAGGGAGGGTGTGACACCAGACATTATCATTAACCCCCACGCCATTCCGTCGCGAATG
ACAATTGCACATTTGATTGAATGCCTCCTCAGTAAGGTGTCAACGCTCGAAGGCATGGAGGGTGATGCAACACCTTTCACCGATGTCACTGTCG
ACTCCGTTTCGGAGCTGCT
>31 ExFu6g-OR891727
TC-CTT-TGCCCATCGATTT----------CC--CCTA------CGACTCGAAGCGTGCCCG--CTACCCCGCTCGAGACC-AAGAATCTTGCAATA-
TGACCGTAATTTTTTT---GGTGGGGCAC-TT-ACCCCGCCACTTGAGCGACGGGAGCGTTTGCCCTCTTAAC---------CATTCTCACAACCTCA--
ATGAGTGCGTCGT-CACGTG-TCAAG--CAGTCACTAACCATTC--
AACAATAGGAAGCCGCTGAGCTCGGTAAGGGTTCCTTCAAGTACGCCTGGGTTCTTGACAAGCTCAAGGCCGAGCGTGAGCGTGGTATCATGA
ACATACCAC-T-TGTTGCCTCGGCGG--ATCAGCCC--GCTCCCGGTAA-AACGGGACGG---CCCGCCAGAGGACCCCTAAAC-TC---TGTTTCT--
ATATGTAACTTCTGAGTAAAACCA-
TAAATAAATCAAAACTTTCAACAACGGATCTCTTGGTTCTGGCATCGATGAAGAACGCAGCAAAATGCGATAAGTAATGTGAATTGCAGAATT
CAGTGAATCATCGAATCTTTGAACGCACATTGCGCCCGCCAGTATTCTGGCGGGCATGCCTGTTCGAGCGTCATTTC-AACCCTCAAGCAC---
AGCT-TGGTGTTGGGACTCG------CG--TTAATTCG---------CGTTCCCCAAATTGATTGGCGGTCACGTCG-AGCTTCCATAGCGTAGTAGTAAAAC-
CCTCGTTACTGGTAATCGTCGCGGCCACGCCGTTAAACC-
CCCGGTCAAAACGCCATTGTTGCAATTGCTTGCTACTCAGGTTACAACCAGGAAGATTCCGTCATTATGAACCAGAGTAGTATTGATCGAGGTC
TGTTCCGAAGTCTGTTCTTCCGATCGTACTCAGATCAGGAGAAGAAGGTTGGTCTCAACTACACTGAGATCTTTGAGAAACCTTTCCAGCAGAC
AACGCTTCGAATGAAGCATGGAACATACGACAAGCTTGATGAAGATGGTATCGTGGCTCCTGGTGTCCGTGTGTCAGGTGAAGATATCATTATC
GGCAAGACTGCACCCATCGACCAAGAAAACCAGGACCTTGGCACAAGAACTCAATCGCACCAACGTCGTGATATCTCGACACCACTGCGAAG
TACTGAGAACGGTATCGTTGATCAAGTCATTCTGACAGTCAACGCCGATAACGTCAAGTACGTCAAGGTCCGAGTACGAACAACCAAGATTCC
TCAAATTGGTGACAAGTTTGCTTCTCGTCACGGTCAAAAGGGTACAATCGGTGTTACATATCGACAGGAGGATATGCCTTTCAGCCGAGAAGGT
CTTACTCCCGATATCATTATCAACCCTCACGCCATTCCATCGCGAATGACAATTGCCCATTTGATTGAGTGTCTTCTTAGCAAGGTTTCAACGCT
GGAAGGTATGGAGGGTGACGCCACACCGTTCACTGATGTCACAGTCGATTCAGTCTCAGAACTTCT
>32 ExFu7h-OR891728
TTGCTATTCCACATCGAATTCCCCGTCGAATTCCCTC-------CCTCGCGATACGCTCTGCGCCCGCTTCTCCCGAGTCCCAAAATTTTTGCGGTC-
CGACCGTAATTTTTTT---GGTGGGGCATTT--ACCCCGCCACTCGGGCGACGTTGGACAAAGCCCTGATCCCTGCACACAAAAA-
CACCAAATCCTCT-TGGCGCGCATCAT-CACGTGGTTCACGACAGACGCTAACTGGTCC-
AACAATAGGAAGCCGCTGAGCTCGGTAAGGGTTCCTTCAAGTACGCCTGGGTCCTTGACAAGCTCAAGGCCGAGCGTGAGCGTGGTATCATGA
ACATACCTAAAACGTTGCTTCGGCGGGAA-CAGACG----GCCCTGTAACAACGGGCCGCC--CCCGCCAGAGGACCCC-TAAC-TC---TGTTTTT-
ATAATGTTT-TTCTGAGTAAA-
CAAGCAAATAAATTAAAACTTTCAACAACGGATCTCTTGGCTCTGGCATCGATGAAGAACGCAGCGAAATGCGATAAGTAATGTGAATTGCAG
AATTCAGTGAATCATCGAATCTTTGAACGCACATTGCGCCCGCCAGTATTCTGGCGGGCATGCCTGTTCGAGCGTCATTAC-
AACCCTCAGGCCCCCGGGCC-TGGCGTTGGGGATCGGCA-GAAGCCCCC-
TGTGGGCACACGCCGTCCCTCAAATACAGTGGCGGTCCCGCCGCAGCTTCCATTGCGTAGTAGCTA-
ACACCTCGCAACTGGAGAGCGGCGCGGCCATGCCGTAAAACACCCCGGTCAGAACGCCATCGTCGCTATCGCTTGCTACTCTGGTTACAACCA
GGAAGATTCCGTCATTATGAACCAGAGTAGTATCGATCGAGGCCTGTTCCGCAGTCTGTTCTTCAGATCCTACTCTGACCAGGAGAAGAAGGTC
GGTCTGAACTACACGGAAGTATTCGAGAAGCCCTTCCAGCAGTCGACGCTTCGTATGAAGCATGGTACCTACGACAAGCTGGACGAGGATGGT
ATCGTGGCCCCTGGTGTGCGAGTGTCAGGTGAAGATATCATCATCGGCAAGACTGCGCCGATTGATCAAGAGAACCAGGATCTGGGTACCAGG
ACAACGGTGCACCAGCGTCGTGATATCTCCACGCCGCTGCGAAGTACCGAGAACGGTATCGTCGATTCGGTCATTGTGACTGTCAATGCCGAC
AACGTCAAGTACGTCAAGGTCCGTGTGAGGACGACCAAGATTCCCCAGATTGGTGACAAGTTCGCCTCTCGTCACGGACAGAAGGGTACCATT
GGTGTTACCTACCGACAGGAGGATATGCCCTTCACCAGGGAGGGTGTGACACCAGACATTATCATTAACCCCCACGCCATTCCGTCGCGAATG
ACAATTGCACATTTGATTGAATGCCTCCTCAGTAAGGTGTCAACGCTCGAAGGCATGGAGGGTGATGCAACACCTTTCACCGATGTCACTGTCG
ACTCCGTTTCGGAGCTGCT
>33 ExFu8i-OR891729
TTGCTATTCCACATCGAATTCCC------------TC-------CCTCGCGATACGCTCTGCGCCCGCTTCTCCCGAGTCCCAAAATTTTTGCGGTC-
CAACCGTAATTTTTTTTT-GGTGGGGCATTT--
ACCCCGCCACTCGGGCGACGTTGGACAAAGCCCTGATCCCTGCACACAAAAAACACCAAACCCTCT-TGGCGCGCATCAT-
CACGTGGTTCACAACAGACGCTAACCGGTCC-
AACAATAGGAAGCCGCTGAGCTCGGTAAGGGTTCCTTCAAGTACGCCTGGGTCCTTGACAAGCTCAAGGCCGAGCGTGAGCGTGGTATCATGA
ACATACCTAAAACGTTGCTTCGGCGGGAA-CAGACG----GCCCCGTGA-AACGGGCCGCC--CCCGCCAGAGGACCCCCTAAC-TC---TGTTGCT-
ATA-TGTATCTTCTGAGTAAA-
CAAGCAAATAAATTAAAACTTTCAACAACGGATCTCTTGGCTCTGGCATCGATGAAGAACGCAGCGAAATGCGATAAGTAATGTGAATTGCAG
AATTCAGTGAATCATCGAATCTTTGAACGCACATTGCGCCCGCCAGTATTCTGGCGGGCATGCCTGTTCGAGCGTCATTAC-

AACCCTCAGGCCCCCGGGCC-
TGGCGTTGGGGATCGGCGAGGCGCCCCCCTGCGGGCACGCGCCGTCCCCCAAATACAGTGGCGGTCCCGCCGCAGCTTCCATTGCGTAGTAGC
TA-
ACACCTCGCAACTGGAGAGCGGCGCGGCCATGCCGTAAAACACCCCGGTCAGAACGCCATCGTCGCTATCGCTTGTTACTCTGGTTACAACCA
AGAAGATTCCGTCATTATGAACCAGAGTAGTATCGATCGAGGCCTGTTCCGCAGTCTGTTCTTCAGATCCTACTCTGACCAGGAGAAGAAGGTC
GGTCTGAACTACACGGAAGTATTCGAGAAGCCCTTCCAGCAGTCGACGCTTCGTATGAAGCACGGTACCTACGACAAGCTGGACGAGGATGGT
ATCGTGGCTCCCGGTGTGCGAGTGTCAGGTGAAGATATCATCATCGGCAAGACTGCGCCGATTGATCAAGAGAACCAGGATCTGGGTACCAGG
ACAACGGTGCACCAGCGTCGTGATATCTCCACGCCGCTGCGAAGTACCGAGAACGGTATCGTCGATTCGGTCATTGTGACTGTCAATGCCGAC
AACGTCAAGTACGTCAAGGTCCGTGTGAGGACGACCAAGATTCCTCAGATTGGTGACAAGTTTGCCTCTCGTCACGGACAGAAGGGTACCATT
GGTGTCACCTACCGACAGGAGGATATGCCCTTTACCAGGGAGGGCGTGACACCAGACATTATCATTAACCCCCACGCCATTCCCTCGCGAATG
ACAATTGCACATTTGATTGAATGCCTCCTCAGTAAGGTGTCAACGCTCGAAGGCATGGAGGGTGATGCAACACCTTTCACCGATGTCACTGTCG
ACTCCGTTTCGGAGCTGCT
>34 NoFu2B-OR891730
TC-CTT-TGCCCATCGATTT----------CC--CCTA------CGACTCGAAACGTGCCCG--CTACCCCGCTCGAGACC-AAAAATTTTGCAATA-
TGACTGTAATTTTTTTT--GGTGGGGCAC-TT-ACCCCGCCACTTGAGCGACGGGAGCGTTTGCCCTCTTAAC---------CATTCTCACAACCTCA--
ATGAGTGCGTCGT-CACGTG-TCAAG--CAGTCACTAACCATTC--
AACAATAGGAAGCCGCTGAGCTCGGTAAGGGTTCCTTCAAGTACGCCTGGGTTCTTGACAAGCTCAAGGCCGAGCGTGAGCGTGGTATCATGA
ACATACCAC-T-TGTTGCCTCGGCGG--ATCAGCCC--GCTCCCGGTAA-AACGGGACGG---CCCGCCAGAGGACCCCTAAAC-TC---TGTTTCT--
ATATGTAACTTCTGAGTAAAACCA-
TAAATAAATCAAAACTTTCAACAACGGATCTCTTGGTTCTGGCATCGATGAAGAACGCAGCAAAATGCGATAAGTAATGTGAATTGCAGAATT
CAGTGAATCATCGAATCTTTGAACGCACATTGCGCCCGCCAGTATTCTGGCGGGCATGCCTGTTCGAGCGTCATTTC-AACCCTCAAGCAC---
AGCT-TGGTGTTGGGACTCG------CG--TTAATTCG---------CGTTCCTCAAATTGATTGGCGGTCACGTCG-AGCTTCCATAGCGTAGTAGTAAAAC-
CCTCGTTACTGGTAATCGTCGCGGCCACGCCGTTAAACC-
CCCGGTCAAAACGCCATTGTTGCAATTGCTTGCTACTCAGGTTACAACCAGGAAGATTCCGTCATTATGAACCAGAGTAGTATTGATCGAGGTC
TGTTCCGAAGTCTGTTCTTCCGATCGTACTCAGATCAGGAGAAGAAGGTTGGTCTCAACTACACTGAGATCTTTGAGAAACCTTTCCAGCAGAC
AACGCTTCGAATGAAGCATGGAACATACGACAAGCTTGATGAAGATGGTATCGTGGCTCCTGGTGTCCGTGTGTCAGGTGAAGATATCATTATC
GGCAAGACTGCACCCATCGACCAAGAAAACCAGGACCTTGGCACAAGAACTCAATCGCACCAACGTCGTGATATCTCGACACCACTGCGAAG
TACTGAGAACGGTATCGTTGATCAAGTCATTCTGACAGTCAACGCCGATAACGTCAAGTACGTCAAGGTCCGAGTACGAACAACCAAGATTCC
TCAAATTGGTGACAAGTTTGCTTCTCGTCACGGTCAAAAGGGTACAATCGGTGTTACATATCGACAGGAGGATATGCCTTTCAGCCGGGAAGGT
CTTACTCCCGATATCATTATCAACCCTCACGCCATTCCATCGCGAATGACAATTGCCCATTTGATTGAGTGTCTTCTTAGCAAGGTTTCAACGCT
GGAAGGTATGGAGGGTGACGCCACACCGTTCACTGATGTCACAGTCGATTCAGTCTCAGAACTTCT
>35 NoFu3C-OR891731
TTGATATTCCACATCGAATTCCCCGTCGAATTCCCTC-------CATCGCGATACGCTCTGCGCCCGCTTCTCCCGAGTCCCAAAATTTTTGCGGTC-
CGACCGTAATTTTTTT---GGTGGGGCATTT--ACCCCGCCACTCGGGCGACGTTGGACAAAGCCCTGATCCCTGCACACAAAAA-
CACCAAACCCTCT-TGGCGCGCATCAT-CACGTGGTTCACGACAGACGCTAACCGGTCC-
AACAATAGGAAGCCGCTGAGCTCGGTAAGGGTTCCTTCAAGTACGCCTGGGTCCTTGACAAGCTCAAGGCCGAGCGTGAGCGTGGTATCATGA
ACATACCTAAAACGTTGCTTCGGCGGGAA-CAGACG----GCCCTGTAACAACGGGCCGCC--CCCGCCAGAGGACCCC-TAAC-TC---TGTTTTT-
ATAATGTTT-TTCTGAGTAAA-
CAAGCAAATAAATTAAAACTTTCAACAACGGATCTCTTGGCTCTGGCATCGATGAAGAACGCAGCGAAATGCGATAAGTAATGTGAATTGCAG
AATTCAGTGAATCATCGAATCTTTGAACGCACATTGCGCCCGCCAGTATTCTGGCGGGCATGCCTGTTCGAGCGTCATTAC-
AACCCTCAGGCCCCCGGGCC-TGGCGTTGGGGATCGGCG-GAAGCCCCC-
TGTGGGCACACGCCGTCCCTCAAATACAGTGGCGGTCCCGCCGCAGCTTCCATTGCGTAGTAGCTA-
ACACCTCGCAACTGGAGAGCGGCGCGGCCATGCCGTAAAACACCCCGGTCAGAACGCCATCGTCGCTATCGCTTGCTACTCTGGTTACAACCA
GGAAGATTCCGTCATTATGAACCAGAGTAGTATCGATCGAGGCCTGTTCCGCAGTCTGTTCTTCAGATCCTACTCTGACCAGGAGAAGAAGGTC
GGTCTGAACTACACGGAAGTATTCGAGAAGCCCTTCCAGCAGTCGACGCTTCGTATGAAGCATGGTACCTACGACAAGCTGGACGAGGATGGT
ATCGTGGCCCCTGGTGTGCGAGTGTCAGGTGAAGATATCATCATCGGCAAGACTGCGCCGATTGATCAAGAGAACCAGGATCTGGGTACCAGG
ACAACGGTGCACCAGCGTCGTGATATCTCCACGCCGCTGCGAAGTACCGAGAACGGTATCGTCGATTCGGTCATTGTGACTGTCAATGCCGAC
AACGTCAAGTACGTCAAGGTCCGTGTGAGGACGACCAAGATTCCCCAGATTGGTGACAAGTTCGCCTCTCGTCACGGACAGAAGGGTACCATT
GGTGTTACCTACCGACAGGAGGATATGCCCTTCACCAGGGAGGGTGTGACACCAGACATTATCATTAACCCCCACGCCATTCCGTCGCGAATG
ACAATTGCACATTTGATTGAATGCCTCCTCAGTAAGGTGTCAACGCTCGAAGGCATGGAGGGTGATGCAACACCTTTCACCGATGTCACTGTCG
ACTCCGTTTCGGAGCTGCT
>36 NoFu4D-OR891732
TC-CTT-TGCCCATCGATTT----------CC--CCTA------CGACTCGAAACGTGCCCG--CTACCCCGCTCGAGACC-AAAAATTTTGCAATA-
TGACTGTAATTTTTTTT--GGTGGGGCAC-TT-ACCCCGCCACTTGAGCGACGGGAGCGTTTGCCCTCTTAAC---------CATTCTCACAACCTCA--
ATGAGTGCGTCGT-CACGTG-TCAAG--CAGTCACTAACCATTC--
AACAATAGGAAGCCGCTGAGCTCGGTAAGGGTTCCTTCAAGTACGCCTGGGTTCTTGACAAGCTCAAGGCCGAGCGTGAGCGTGGTATCATGA
ACATACCAC-T-TGTTGCCTCGGCGG--ATCAGCCC--GCTCCCGGTAA-AACGGGACGG---CCCGCCAGAGGACCCCTAAAC-TC---TGTTTCT--
ATATGTAACTTCTGAGTAAAACCA-
TAAATAAATCAAAACTTTCAACAACGGATCTCTTGGTTCTGGCATCGATGAAGAACGCAGCAAAATGCGATAAGTAATGTGAATTGCAGAATT
CAGTGAATCATCGAATCTTTGAACGCACATTGCGCCCGCCAGTATTCTGGCGGGCATGCCTGTTCGAGCGTCATTTC-AACCCTCAAGCAC---
AGCT-TGGTGTTGGGACTCG------CG--TTAATTCG---------CGTTCCCCAAATTGATTGGCGGTCACGTCG-AGCTTCCATAGCGTAGTAGTAAAAC-
CCTCGTTACTGGTAATCGTCGCGGCCACGCCGTTAAACC-
CCCGGTCAAAACGCCATTGTTGCAATTGCTTGCTACTCAGGTTACAACCAGGAAGATTCCGTCATTATGAACCAGAGTAGTATTGATCGAGGTC
TGTTCCGAAGTCTGTTCTTCCGATCGTACTCAGATCAGGAGAAGAAGGTTGGTCTCAACTACACTGAGATCTTTGAGAAACCTTTCCAGCAGAC
AACGCTTCGAATGAAGCATGGAACATACGACAAGCTTGATGAAGATGGTATCGTGGCTCCTGGTGTCCGTGTGTCAGGTGAAGATATCATTATC
GGCAAGACTGCACCCATCGACCAAGAAAACCAGGACCTTGGCACAAGAACTCAATCGCACCAACGTCGTGATATCTCGACACCGCTGCGAAG
TACTGAGAACGGTATCGTTGATCAAGTCATTCTGACAGTCAACGCCGATAACGTCAAGTACGTCAAGGTCCGAGTACGAACAACCAAGATTCC
TCAAATTGGTGACAAGTTTGCTTCTCGTCACGGTCAAAAGGGTACAATCGGTGTTACATATCGACAGGAGGATATGCCTTTCAGCCGAGAAGGT

CTTACTCCCGATATCATTATCAACCCTCACGCCATTCCATCGCGAATGACAATTGCCCATTTGATTGAGTGTCTTCTTAGCAAGGTTTCAACGCT
GGAAGGTATGGAGGGTGACGCCACACCGTTCACTGATGTCACAGTCGATTCAGTTTCAGAACTTCT
>37 NoFu18Q-OR891733
TTGATATTCCACATCGAATTCCCCGTCGAATTCCCTC-------CATCGCGATACGCTCTGCGCCCGCTTCTCCCGAGTCCCAAAATTTTTGCGGTC-
CGACCGTAATTTTTTT---GGTGGGGCATTT--ACCCCGCCACTCGGGCGACGTTGGACAAAGCCCTGATCCCTGCACACAAAAA-
CACCAAACCCTCT-TGGCGCGCATCAT-CACGTGGTTCACGACAGACGCTAACCGGTCC-
AACAATAGGAAGCCGCTGAGCTCGGTAAGGGTTCCTTCAAGTACGCCTGGGTCCTTGACAAGCTCAAGGCCGAGCGTGAGCGTGGTATCATGA
ACATACCTAAAACGTTGCTTCGGCGGGAA-CAGACG----GCCCTGTAACAACGGGCCGCC--CCCGCCAGAGGACCCC-TAAC-TC---TGTTTTT-
ATAATGTTT-TTCTGAGTAAA-
CAAGCAAATAAATTAAAACTTTCAACAACGGATCTCTTGGCTCTGGCATCGATGAAGAACGCAGCGAAATGCGATAAGTAATGTGAATTGCAG
AATTCAGTGAATCATCGAATCTTTGAACGCACATTGCGCCCGCCAGTATTCTGGCGGGCATGCCTGTTCGAGCGTCATTAC-
AACCCTCAGGCCCCCGGGCC-TGGCGTTGGGGATCGGCG-GAAGCCCCC-
TGTGGGCACACGCCGTCCCTCAAATACAGTGGCGGTCCCGCCGCAGCTTCCATTGCGTAGTAGCTA-
ACACCTCGCAACTGGAGAGCGGCGCGGCCATGCCGTAAAACACCCCGGTCAGAACGCCATCGTTGCTATCGCTTGCTACTCTGGTTACAACCA
GGAAGATTCCGTCATTATGAACCAGAGTAGTATCGATCGAGGCCTGTTCCGCAGTCTGTTCTTCAGATCCTACTCTGACCAGGAGAAGAAGGTC
GGTCTGAACTACACGGAAGTATTCGAGAAGCCCTTCCAGCAGTCGACGCTTCGTATGAAGCATGGTACCTACGACAAGCTGGACGAGGATGGT
ATCGTGGCCCCTGGTGTGCGAGTGTCAGGTGAAGATATCATCATCGGCAAGACTGCGCCGATTGATCAAGAGAACCAGGATCTGGGTACCAGG
ACAACGGTGCACCAGCGTCGTGATATCTCCACGCCGCTGCGAAGTACCGAGAACGGTATCGTCGATTCGGTCATTGTGACTGTCAATGCCGAC
AACGTCAAGTACGTCAAGGTCCGTGTGAGGACGACCAAGATTCCCCAGATTGGTGACAAGTTCGCCTCTCGTCACGGACAGAAGGGTACCATT
GGTGTTACCTACCGACAGGAGGATATGCCCTTCACCAGGGAGGGTGTGACACCAGACATTATCATTAACCCCCACGCCATTCCGTCGCGAATG
ACAATTGCACATTTGATTGAATGCCTCCTCAGTAAGGTGTCAACGCTCGAAGGCATGGAGGGTGATGCAACACCTTTCACCGATGTCACTGTCG
ACTCCGTTTCGGAGCTGCT
>38 SLFUA-OR891734
TC-CTT-TGCCCATCGATTT----------CC--CCTA------CGACTCGAAACGTGCCCG--CTACCCCGCTCGAGACC-AAAAATTTTGCAATA-
TGACTGTAATTTTTTTT--GGTGGGGCAC-TT-ACCCCGCCACTTGAGCGACGGGAGCGTTTGCCCTCTTAAC---------CATTCTCACAACCTCA--
ATGAGTGCGTCGT-CACGTG-TCAAG--CAGTCACTAACCATTC--
AACAATAGGAAGCCGCTGAGCTCGGTAAGGGTTCCTTCAAGTACGCCTGGGTTCTTGACAAGCTCAAGGCCGAGCGTGAGCGTGGTATCATGA
ACATACCAC-T-TGTTGCCTCGGCGG--ATCAGCCC--GCTCCCGGTAA-AACGGGACGG---CCCGCCAGAGGACCCCTAAAC-TC---TGTTTCT--
ATATGTAACTTCTGAGTAAAACCA-
TAAATAAATCAAAACTTTCAACAACGGATCTCTTGGTTCTGGCATCGATGAAGAACGCAGCAAAATGCGATAAGTAATGTGAATTGCAGAATT
CAGTGAATCATCGAATCTTTGAACGCACATTGCGCCCGCCAGTATTCTGGCGGGCATGCCTGTTCGAGCGTCATTTC-AACCCTCAAGCAC---
AGCT-TGGTGTTGGGACTCG------CG--TTAATTCG---------CGTTCCCCAAATTGATTGGCGGTCACGTCG-AGCTTCCATAGCGTAGTAGTAAAAC-
CCTCGTTACTGGTAATCGTCGCGGCCACGCCGTTAAACC-
CCCGGTCAAAACGCCATTGTTGCAATTGCTTGCTACTCAGGTTACAACCAGGAAGATTCCGTCATTATGAACCAGAGTAGTATTGATCGAGGTC
TGTTCCGAAGTCTGTTCTTCCGATCGTACTCAGATCAGGAGAAGAAGGTTGGTCTCAACTACACTGAGATCTTTGAGAAACCTTTCCAGCAGAC
AACGCTTCGAATGAAGCATGGAACATACGACAAGCTTGATGAAGATGGTATCGTGGCTCCTGGTGTCCGTGTGTCAGGTGAAGATATCATTATC
GGCAAGACTGCACCCATCGACCAAGAAAACCAGGACCTTGGCACAAGAACTCAATCGCACCAACGTCGTGATATCTCGACACCACTGCGAAG
TACTGAGAACGGTATCGTTGATCAAGTCATTCTGACAGTCAACGCCGATAACGTCAAGTACGTCAAGGTCCGAGTACGAACAACCAAGATTCC
TCAAATTGGTGACAAGTTTGCTTCTCGTCACGGTCAAAAGGGTACAATCGGTGTTACATATCGACAGGAGGATATGCCTTTCAGCCGAGAAGGT
CTTACTCCCGATATCATTATCAACCCTCACGCCATTCCATCGCGAATGACAATTGCCCATTTGATTGAGTGTCTTCTTAGCAAGGTTTCAACGCT
GGAAGGTATGGAGGGTGACGCCACACCGTTCACTGATGTCACAGTCGATTCAGTCTCAGAACTTCT
>39 SLFUD-OR891735
TTGCTATTCCACATCGAATTCCCCGTCGAATTCCCTC-------CCTCGCGATACGCTCTGCGCCCGCTTCTCCCGAGTCCCAAAATTTTTGCGGTC-
CGACCGTAATTTTTTT---GGTGGGGCATTT--ACCCCGCCACTCGGGCGACGTTGGACAAAGCCCTGATCCCTGCACACAAAAA-
CACCAAATCCTCT-TGGCGCGCATCAT-CACGTGGTTCACGACAGACGCTAACTGGTCC-
AACAATAGGAAGCCGCTGAGCTCGGTAAGGGTTCCTTCAAGTACGCCTGGGTCCTTGACAAGCTCAAGGCCGAGCGTGAGCGTGGTATCATGA
ACATACCTAAAACGTTGCTTCGGCGGGAA-CAGACG----GCCCTGTAACAACGGGCCGCC--CCCGCCAGAGGACCCC-TAAC-TC---TGTTTTT-
ATAATGTTT-TTCTGAGTAAA-
CAAGCAAATAAATTAAAACTTTCAACAACGGATCTCTTGGCTCTGGCATCGATGAAGAACGCAGCGAAATGCGATAAGTAATGTGAATTGCAG
AATTCAGTGAATCATCGAATCTTTGAACGCACATTGCGCCCGCCAGTATTCTGGCGGGCATGCCTGTTCGAGCGTCATTAC-
AACCCTCAGGCCCCCGGGCC-TGGCGTTGGGGATCGGCA-GAAGCCCCC-
TGTGGGCACACGCCGTCCCTCAAATACAGTGGCGGTCCCGCCGCAGCTTCCATTGCGTAGTAGCTA-
ACACCTCGCAACTGGAGAGCGGCGCGGCCATGCCGTAAAACACCCCGGTCAGAACGCCATCGTCGCTATCGCTTGCTACTCTGGTTACAACCA
GGAAGATTCCGTCATTATGAACCAGAGTAGTATCGATCGAGGCCTGTTCCGCAGTCTGTTCTTCAGATCCTACTCTGACCAGGAGAAGAAGGTC
GGTCTGAACTACACGGAAGTATTCGAGAAGCCCTTCCAGCAGTCGACGCTTCGTATGAAGCATGGTACCTACGACAAGCTGGACGAGGATGGT
ATCGTGGCCCCTGGTGTGCGAGTGTCAGGTGAAGATATCATCATCGGCAAGACTGCGCCGATTGATCAAGAGAACCAGGATCTGGGTACCAGG
ACAACGGTGCACCAGCGTCGTGATATCTCCACGCCGCTGCGAAGTACCGAGAACGGTATCGTCGATTCGGTCATTGTGACTGTCAATGCCGAC
AACGTCAAGTACGTCAAGGTCCGTGTGAGGACGACCAAGATTCCCCAGATTGGTGACAAGTTCGCCTCTCGTCACGGACAGAAGGGTACCATT
GGTGTTACCTACCGACAGGAGGATATGCCCTTCACCAGGGAGGGTGTGACACCAGACATTATCATTAACCCCCACGCCATTCCGTCGCGAATG
ACAATTGCACATTTGATTGAATGCCTCCTCAGTAAGGTGTCAACGCTCGAAGGCATGGAGGGTGATGCAACACCTTTCACCGATGTCACTGTCG
ACTCCGTTTCGGAGCTGCT
>40 SLFUM-OR891736
TTGCTATCCCACATCGAATTCCCCGTCGAATTCCCTC-------CTCCGCGACACGCTCTGCGCCCGCTTCTCCCGAGTCCCAAAAATTTTGCGGTT-
CGACCGCAATTTTTTTTTTTGGTGGGGCATTT--ACCCCGCCACTCGGGCGACGTTGGACAAAGCCCTGATCCCTGCACACAAAAA-
CACCAAACCCTCT-TGGCGCGCATCA----CGTGGTTCACAACAGACACTGACTGGTTC-
AACAATAGGAAGCCGCTGAGCTCGGTAAGGGTTCCTTCAAGTACGCCTGGGTCCTTGACAAGCTCAAGGCCGAGCGTGAGCGTGGTATCATGA
ACATACCTAAA-CGTTGCTTCGGCGGGAA-CAGACG----GCCCCGTGA-AACGGGCCGCC--CCCGCCAGAGGACCCC-TAAC-TC---TGTTTCT-
ATAATGTTTCTTCTGAGTAAAACAAGCAAATAAATTAAAACTTTCAACAACGGATCTCTTGGCTCTGGCATCGATGAAGAACGCAGCGAAATG
CGATAAGTAATGTGAATTGCAGAATTCAGTGAATCATCGAATCTTTGAACGCACATTGCGCCCGCCAGTATTCTGGCGGGCATGCCTGTTCGAG

CGTCATTAC-AACCCTCAGGCCCCCGGGCC-TGGCGTTGGGGATCGGCG-GA-GCCCCC-
CGTGGGCACACGCCGTCCCCCAAATACAGTGGCGGTCCCGCCGCAGCTTCCATCGCGTAGTAGCTA-
ACACCTCGCGACTGGAGAGCGGCGCGGCCACGCCGTAAAACACCCCGGTCAGAACGCCATCGTCGCTATCGCTTGCTACTCTGGTTATAACCA
GGAAGATTCCGTCATTATGAACCAGAGTAGTATCGATCGAGGCCTGTTCCGCAGTCTGTTCTTCCGATCCTACTCTGACCAGGAGAAGAAGGTC
GGCCTCAACTACACGGAAGTGTTTGAGAAGCCCTTCCAGCAGTCGACGCTTCGTATGAAGCACGGTACCTACGACAAGCTGGACGAGGATGGT
ATCGTGGCCCCCGGTGTGCGAGTGTCAGGTGAAGATATCATCATCGGCAAGACCGCGCCGATTGATCAAGAGAACCAGGATCTGGGTACCAG
GACAACGGTGCACCAGCGTCGTGATATCTCCACGCCGCTGCGAAGTACCGAGAACGGTATCGTCGATTCGGTCATCGTGACTGTCAATGCCGA
CAACGTCAAGTACGTCAAGGTCCGTGTGAGGACGACCAAGATTCCTCAGATTGGTGACAAGTTCGCCTCTCGTCACGGACAGAAGGGTACCAT
TGGTGTTACCTACCGACAGGAGGACATGCCCTTTAGCAGGGAGGGCGTGACACCAGACATTATCATTAACCCCCACGCCATTCCGTCGCGAAT
GACAATTGCCCATTTGATTGAATGCCTCCTCAGTAAGGTGTCAACGCTCGAAGGCATGGAGGGTGATGCCACACCTTTCACCGACGTCACTGTC
GACTCCGTTTCGGAGCTGCT
>41 Fo-TEF
TTGATATTCCACATCGAATTCCCCGTCGAATTCCCTC-------CATCGCGATACGCTCTGCGCCCGCTTCTCCCGAGTCCCAAAATTTTTGCGGTC-
CGACCGTAATTTTTTT---GGTGGGGCATTT--ACCCCGCCACTCGGGCGACGTTGGACAAAGCCCTGATCCCTGCACACAAAAA-
CACCAAACCCTCT-TGGCGCGCATCAT-CACGTGGTTCACGACAGACGCTAACCGGTCC-
AACAATAGGAAGCCGCTGAGCTCGGTAAGGGTTCCTTCAAGTACGCCTGGGTCCTTGACAAGCTCAAGGCCGAGCGTGAGCGTGGTATCATGA
ACATACCTAAAACGTTGCTTCGGCGGGAA-CAGACG----GCCCTGTAACAACGGGCCGCC--CCCGCCAGAGGACCCC-TAAC-TC---TGTTTTT-
ATAATGTTT-TTCTGAGTAAA-
CAAGCAAATAAATTAAAACTTTCAACAACGGATCTCTTGGCTCTGGCATCGATGAAGAACGCAGCGAAATGCGATAAGTAATGTGAATTGCAG
AATTCAGTGAATCATCGAATCTTTGAACGCACATTGCGCCCGCCAGTATTCTGGCGGGCATGCCTGTTCGAGCGTCATTAC-
AACCCTCAGGCCCCCGGGCC-TGGCGTTGGGGATCGGCG-GAAGCCCCC-
TGTGGGCACACGCCGTCCCTCAAATACAGTGGCGGTCCCGCCGCAGCTTCCATTGCGTAGTAGCTA-
ACACCTCGCAACTGGAGAGCGGCGCGGCCATGCCGTAAAACACCCCGGTCAGAACGCCATCGTCGCTATCGCTTGCTACTCTGGTTACAACCA
GGAAGATTCCGTCATTATGAACCAGAGTAGTATCGATCGAGGCCTGTTCCGCAGTCTGTTCTTCAGATCCTACTCTGACCAGGAGAAGAAGGTC
GGTCTGAACTACACGGAAGTATTCGAGAAGCCCTTCCAGCAGTCGACGCTTCGTATGAAGCATGGTACCTACGACAAGCTGGACGAGGATGGT
ATCGTGGCCCCTGGTGTGCGAGTGTCAGGTGAAGATATCATCATCGGCAAGACTGCGCCGATTGATCAAGAGAACCAGGATCTGGGTACCAGG
ACAACGGTGCACCAGCGTCGTGATATCTCCACGCCGCTGCGAAGTACCGAGAACGGTATCGTCGATTCGGTCATTGTGACTGTCAATGCCGAC
AACGTCAAGTACGTCAAGGTCCGTGTGAGGACGACCAAGATTCCCCAGATTGGTGACAAGTTCGCCTCTCGTCACGGACAGAAGGGTACCATT
GGTGTTACCTACCGACAGGAGGATATGCCCTTCACCAGGGAGGGTGTGACACCAGACATTATCATTAACCCCCACGCCATTCCGTCGCGAATG
ACAATTGCACATTTGATTGAATGCCTCCTCAGTAAGGTGTCAACGCTCGAAGGCATGGAGGGTGATGCAACACCTTTCACCGATGTCACTGTCG
ACTCCGTTTCGGAGCTGCT
>42 Fs-TEF
TTGATATTCCACATCGAATTCCCCGTCGAATTCCCTC-------CATCGCGATACGCTCTGCGCCCGCTTCTCCCGAGTCCCAAAATTTTTGCGGTC-
CGACCGTAATTTTTTT---GGTGGGGCATTT--ACCCCGCCACTCGGGCGACGTTGGACAAAGCCCTGATCCCTGCACACAAAAA-
CACCAAACCCTCT-TGGCGCGCATCAT-CACGTGGTTCACGACAGACGCTAACCGGTCC-
AACAATAGGAAGCCGCTGAGCTCGGTAAGGGTTCCTTCAAGTACGCCTGGGTCCTTGACAAGCTCAAGGCCGAGCGTGAGCGTGGTATCATGA
ACATACCTAAAACGTTGCTTCGGCGGGAA-CAGACG----GCCCTGTAACAACGGGCCGCC--CCCGCCAGAGGACCCC-TAAC-TC---TGTTTTT-
ATAATGTTT-TTCTGAGTAAA-
CAAGCAAATAAATTAAAACTTTCAACAACGGATCTCTTGGCTCTGGCATCGATGAAGAACGCAGCGAAATGCGATAAGTAATGTGAATTGCAG
AATTCAGTGAATCATCGAATCTTTGAACGCACATTGCGCCCGCCAGTATTCTGGCGGGCATGCCTGTTCGAGCGTCATTAC-
AACCCTCAGGCCCCCGGGCC-TGGCGTTGGGGATCGGCG-GAAGCCCCC-
TGTGGGCACACGCCGTCCCTCAAATACAGTGGCGGTCCCGCCGCAGCTTCCATTGCGTAGTAGCTA-
ACACCTCGCAACTGGAGAGCGGCGCGGCCATGCCGTAAAACACCCCGGTCAGAACGCCATCGTCGCTATCGCTTGCTACTCTGGTTACAACCA
GGAAGATTCCGTCATTATGAACCAGAGTAGTATCGATCGAGGCCTGTTCCGCAGTCTGTTCTTCAGATCCTACTCTGACCAGGAGAAGAAGGTC
GGTCTGAACTACACGGAAGTATTCGAGAAGCCCTTCCAGCAGTCGACGCTTCGTATGAAGCATGGTACCTACGACAAGCTGGACGAGGATGGT
ATCGTGGCCCCTGGTGTGCGAGTGTCAGGTGAAGATATCATCATCGGCAAGACTGCGCCGATTGATCAAGAGAACCAGGATCTGGGTACCAGG
ACAACGGTGCACCAGCGTCGTGATATCTCCACGCCGCTGCGAAGTACCGAGAACGGTATCGTCGATTCGGTCATTGTGACTGTCAATGCCGAC
AACGTCAAGTACGTCAAGGTCCGTGTGAGGACGACCAAGATTCCCCAGATTGGTGACAAGTTCGCCTCTCGTCACGGACAGAAGGGTACCATT
GGTGTTACCTACCGACAGGAGGATATGCCCTTCACCAGGGAGGGTGTGACACCAGACATTATCATTAACCCCCACGCCATTCCGTCGCGAATG
ACAATTGCACATTTGATTGAATGCCTCCTCAGTAAGGTGTCAACGCTCGAAGGCATGGAGGGTGATGCAACACCTTTCACCGATGTCACTGTCG
ACTCCGTTTCGGAGCTGCT
>43 MW847905.1-*Fusicolla aquaeductuum*
ATCCAT---CGCACCGACTCCCC----AAGTCGTCGCTCGATCCCTCTTCGA-
ACGCTCCGACCGTTACCCTTCCACACAATGAGATTTTTCTCACTGCCCCATTGAATTTTCTT--GGTGGGGGCGGATTACCCCGCTGCCTGC-
TGAGGTTACAAAATTCTTTGCCCTGCCCCACAGGCCTGCACACACCAACCAACACCATGTCACATGCCCGCGATCCAAACACGATGCTAACTC
TC---
AACAATAGGAAGCTGCCGAGCTCGGCAAGGGTTCCTTCAAGTATGCCTGGGTTCTTGACAAGCTCAAGGCTGAGCGTGAGCGTGGTATCATGA
ACATACCTA--TCGTTGCTTCGGCGG--ATCCGCCCCGGCGCCCTCGGG-CCCGGATCAGGCGCCCGCCGGAGACC--CCAAAC-TCTTGTATTTCT--
TTA-GTATCTTCTGAGTAA-
ACAAGCAAATAAATTAAAACTTTCAACAACGGATCTCTTGGTTCTGGCATCGATGAAGAACGCAGCGAAATGCGATAAGTAATGTGAATTGCA
GAATTCCGTGAATCATCGAATCTTTGAACGCACATTGCGCCCGCCAGTACTCTGGCGGGCATGCCTGTTCGAGCGTCATTTC-
AACCCTCAAGCCCCCGGGCT-TGGTGTTGGGGCTCGGC----CCGTCCCTCGTGG---CGCGCCGTCCCCGAAATCCAGTGGCGGTCACGCTGTA--
CTCCTCTGCGTAGTAATCCAAC--CTCGCAAC-GGGACAAAGCCTGGCCACGCCGTTAAACC-
CCTGGGCAGAATGCCATCGTTGCTATTGCTTGCTACTCGGGTTATAATCAGGAAGATTCCGTTATTATGAACCAGAGCAGTATCGATCGTGGCCT
GTTCCGAAGTTTGTTCTTCCGATCATACTCCGATCAGGAGAAGAAGGTTGGTCTGAACTACACCGAAATCTTCGAGAAGCCCTTCCAGGCATCA
ACTCTTCGTATGAAGCATGGCACATACGACAAGCTTGATGAGGATGGTATCGTGGCACCAGGTGTTCGAGTATCTGGTGAGGATATCATCATTG
GAAAAACAGCGCCGATCGATCAGGAGAATCAGGATCTCGGAACAAGGACTACCCAGCACCAAAGACGAGATATCTCGACGCCCCTCAGAAG
TACCGAGAACGGAATTATTGATCAAGTTATTCTCACGGTCAATGCGGACAACGTCAAGTATGTCAAGGTGCGAGTGCGAACAACCAAGATTCC
CCAGATTGGTGACAAGTTTGCTTCCCGTCACGGTCAGAAGGGTACTATTGGTGTCACATACCGACAGGAAGACATGCCGTTTACCCGAGAAGG

TGTTACGCCTGATATTATCATCAACCCCCACGCCATTCCCTCTCGAATGACCATTGCCCATTTGATTGAGTGTCTTCTGAGCAAGGTCTCAACTCT
GGAGGGTATGGAGGGTGATGCTACCCCCTTTACCGATGTCACCGTCGACTCGGTCTCCGAGCTGCT
>CPC27192
TTGCTATTCCACATCGAATTCCCCGTCGAATTCCCTC-------CCTCGCGATACGCTCTGCGCCCGCTTCTCCCGAGTCCCAAAATTTTTGCGGTC-
CGACCGTAATTTTTTT---GGTGGGGCATTT--ACCCCGCCACTCGGGCGACGTTGGACAAAGCCCTGATCCCTGCACACAAAAA-
CACCAAATCCTCT-TGGCGCGCATCAT-CACGTGGTTCACGACAGACGCTAACTGGTCC-
AACAATAGGAAGCCGCTGAGCTCGGTAAGGGTTCCTTCAAGTACGCCTGGGTCCTTGACAAGCTCAAGGCCGAGCGTGAGCGTGGTATCATGA
ACATACCTAAAACGTTGCTTCGGCGGGAA-CAGACG----GCCCTGTAACAACGGGCCGCC--CCCGCCAGAGGACCCC-TAAC-TC---TGTTTTT-
ATAATGTTT-TTCTGAGTAAA-
CAAGCAAATAAATTAAAACTTTCAACAACGGATCTCTTGGCTCTGGCATCGATGAAGAACGCAGCGAAATGCGATAAGTAATGTGAATTGCAG
AATTCAGTGAATCATCGAATCTTTGAACGCACATTGCGCCCGCCAGTATTCTGGCGGGCATGCCTGTTCGAGCGTCATTAC-
AACCCTCAGGCCCCCGGGCC-TGGCGTTGGGGATCGGCG-GAAGCCCCC-
TGTGGGCACACGCCGTCCCTCAAATACAGTGGCGGTCCCGCCGCAGCTTCCATTGCGTAGTAGCTA-
ACACCTCGCAACTGGAGAGCGGCGCGGCCATGCCGTAAAACACCCCGGTCAGAACGCCATCGTCGCTATCGCTTGCTACTCTGGTTACAACCA
GGAAGATTCCGTCATTATGAACCAGAGTAGTATCGATCGAGGCCTGTTCCGCAGTCTGTTCTTCAGATCCTACTCTGACCAGGAGAAGAAGGTC
GGTCTGAACTACACGGAAGTATTCGAGAAGCCCTTCCAGCAGTCGACGCTTCGTATGAAGCACGGTACCTACGACAAGCTGGACGAGGATGGT
ATCGTGGCCCCTGGTGTGCGAGTGTCAGGTGAAGATATCATCATCGGCAAGACTGCGCCGATTGATCAAGAGAACCAGGATCTGGGTACCAGG
ACAACGGTGCACCAGCGTCGTGATATCTCCACGCCGCTGCGAAGTACCGAGAACGGTATCGTCGATTCGGTCATTGTGACTGTCAATGCCGAC
AACGTCAAGTACGTCAAGGTCCGTGTGAGGACGACCAAGATTCCCCAGATTGGTGACAAGTTCGCCTCTCGTCACGGACAGAAGGGTACCATT
GGTGTTACCTACCGACAGGAGGATATGCCCTTCACCAGGGAGGGTGTGACACCAGACATTATCATTAACCCCCACGCCATTCCGTCGCGAATG
ACAATTGCACATTTGATTGAATGCCTCCTCAGTAAGGTGTCAACGCTCGAAGGCATGGAGGGTGATGCAACACCTTTCACCGATGTCACTGTCG
ACTCCGTTTCGGAGCTGCT
>CPC27198
TTGCTATTCCACATCGAATTCCCCGTCGAATTCCCTC-------CCTCGCGATACGCTCTGCGCCCGCTTCTCCCGAGTCCCAAAATTTTTGCGGTC-
CGACCGTAATTTTTTTTT-GGTGGGGCATTT--ACCCCGCCACTCGGGCGACGTTGGACAAAGCCCTGATCCCTGCACACAAAAA-
CACCAAACCCTCT-TGGCGCGCATCAT-CACGTGGTTCACGACAGACGCTAACCGGTCC-
AACAATAGGAAGCCGCTGAGCTCGGTAAGGGTTCCTTCAAGTACGCCTGGGTCCTTGACAAGCTCAAGGCCGAGCGTGAGCGTGGTATCATGA
ACATACCTAAAACGTTGCTTCGGCGGGAA-CAGACG----GCCCTGTAACAACGGGCCGCC--CCCGCCAGAGGACCCC-TAAC-TC---TGTTTTT-
ATAATGTTT-TTCTGAGTAAA-
CAAGCAAATAAATTAAAACTTTCAACAACGGATCTCTTGGCTCTGGCATCGATGAAGAACGCAGCGAAATGCGATAAGTAATGTGAATTGCAG
AATTCAGTGAATCATCGAATCTTTGAACGCACATTGCGCCCGCCAGTATTCTGGCGGGCATGCCTGTTCGAGCGTCATTAC-
AACCCTCAGGCCCCCGGGCC-TGGCGTTGGGGATCGGCG-GAAGCCCCC-
TGTGGGCACACGCCGTCCCTCAAATACAGTGGCGGTCCCGCCGCAGCTTCCATTGCGTAGTAGCTA-
ACACCTCGCAACTGGAGAGCGGCGCGGCCATGCCGTAAAACACCCCGGTCAGAACGCCATCGTCGCTATCGCTTGCTACTCTGGTTACAACCA
GGAAGATTCCGTCATTATGAACCAGAGTAGTATCGATCGAGGCCTGTTCCGCAGTCTGTTCTTCAGATCCTACTCTGACCAGGAGAAGAAGGTC
GGTCTGAACTACACGGAAGTATTCGAGAAGCCCTTCCAGCAGTCGACGCTTCGTATGAAGCATGGTACCTACGACAAGCTGGACGAGGATGGT
ATCGTGGCCCCTGGTGTGCGAGTGTCAGGTGAAGATATCATCATCGGCAAGACTGCGCCGATTGATCAAGAGAACCAGGATCTGGGTACCAGG
ACAACGGTGCACCAGCGTCGTGATATCTCCACGCCGCTGCGAAGTACCGAGAACGGTATCGTCGATTCGGTCATTGTGACTGTCAATGCCGAC
AACGTCAAGTACGTCAAGGTCCGTGTGAGGACGACCAAGATTCCCCAGATTGGTGACAAGTTCGCCTCTCGTCACGGACAGAAGGGTACCATT
GGTGTTACCTACCGACAGGAGGATATGCCCTTCACCAGGGAGGGTGTGACACCAGACATTATCATTAACCCCCACGCCATTCCGTCGCGAATG
ACAATTGCACATTTGATTGAATGCCTCCTCAGTAAGGTGTCAACGCTCGAAGGCATGGAGGGTGATGCAACACCTTTCACCGATGTCACTGTCG
ACTCCGTTTCGGAGCTGCT
>CPC27193
TTGCTATTCCACATCGAATTCCCCGTCGAATTCCCTC-------CCTCGCGATACGCTCTGCGCCCGCTTCTCCCGAGTCCCAAAATTTTTGCGGTC-
CGACCGTAATTTTTTT---GGTGGGGCATTT--ACCCCGCCACTCGGGCGACGTTGGACAAAGCCCTGATCCCTGCACACAAAAA-
CACCAAATCCTCT-TGGCGCGCATCAT-CACGTGGTTCACGACAGACGCTAACTGGTCC-
AACAATAGGAAGCCGCTGAGCTCGGTAAGGGTTCCTTCAAGTACGCCTGGGTCCTTGACAAGCTCAAGGCCGAGCGTGAGCGTGGTATCATGA
ACATACCTAAAACGTTGCTTCGGCGGGAA-CAGACG----GCCCTGTAACAACGGGCCGCC--CCCGCCAGAGGACCCC-TAAC-TC---TGTTTTT-
ATAATGTTT-TTCTGAGTAAA-
CAAGCAAATAAATTAAAACTTTCAACAACGGATCTCTTGGCTCTGGCATCGATGAAGAACGCAGCGAAATGCGATAAGTAATGTGAATTGCAG
AATTCAGTGAATCATCGAATCTTTGAACGCACATTGCGCCCGCCAGTATTCTGGCGGGCATGCCTGTTCGAGCGTCATTAC-
AACCCTCAGGCCCCCGGGCC-TGGCGTTGGGGATCGGCG-GAAGCCCCC-
TGTGGGCACACGCCGTCCCTCAAATACAGTGGCGGTCCCGCCGCAGCTTCCATTGCGTAGTAGCTA-
ACACCTCGCAACTGGAGAGCGGCGCGGCCATGCCGTAAAACACCCCGGTCAGAACGCCATCGTCGCTATCGCTTGCTACTCTGGTTACAACCA
GGAAGATTCCGTCATTATGAACCAGAGTAGTATCGATCGAGGCCTGTTCCGCAGTCTGTTCTTCAGATCCTACTCTGACCAGGAGAAGAAGGTC
GGTCTGAACTACACGGAAGTATTCGAGAAGCCCTTCCAGCAGTCGACGCTTCGTATGAAGCACGGTACCTACGACAAGCTGGACGAGGATGGT
ATCGTGGCCCCTGGTGTGCGAGTGTCAGGTGAAGATATCATCATCGGCAAGACTGCGCCGATTGATCAAGAGAACCAGGATCTGGGTACCAGG
ACAACGGTGCACCAGCGTCGTGATATCTCCACGCCGCTGCGAAGTACCGAGAACGGTATCGTCGATTCGGTCATTGTGACTGTCAATGCCGAC
AACGTCAAGTACGTCAAGGTCCGTGTGAGGACGACCAAGATTCCCCAGATTGGTGACAAGTTCGCCTCTCGTCACGGACAGAAGGGTACCATT
GGTGTTACCTACCGACAGGAGGATATGCCCTTCACCAGGGAGGGTGTGACACCAGACATTATCATTAACCCCCACGCCATTCCGTCGCGAATG
ACAATTGCACATTTGATTGAATGCCTCCTCAGTAAGGTGTCAACGCTCGAAGGCATGGAGGGTGATGCAACACCTTTCACCGATGTCACTGTCG
ACTCCGTTTCGGAGCTGCT
>FSSC9
TTGCTATTCCACATCGAATTCCCCGTCGAATTCCCTC-------CCTCGCGATACGCTCTGCGCCCGCTTCTCCCGAGTCCCAAAATTTTTGCGGTC-
CGACCGTAATTTTTTTT--GGTGGGGCATTT--
ACCCCGCCACTCGGGCGACGTTGGACAAAGCCCTGATCCCTGCACACAAAAAACACCAAACCCTCT-TGGCGCGCATCAT-
CACGTGGTTCACAACAGACGCTAACTGGTCC-
AACAATAGGAAGCCGCTGAGCTCGGTAAGGGTTCCTTCAAGTACGCCTGGGTCCTTGACAAGCTCAAGGCCGAGCGTGAGCGTGGTATCATGA
ACATACCTAAA-CGTTGCTTCGGCGGGAA-CAGACG----GCCCCGTAA-CACGGGCCGCC--CCCGCCAGAGGACCCCCTAAC-TC---TGTTTTT-

ATAATGTTTTTTCTGAGTAAA-
CAAGCAAATAAATTAAAACTTTCAACAACGGATCTCTTGGCTCTGGCATCGATGAAGAACGCAGCGAAATGCGATAAGTAATGTGAATTGCAG
AATTCAGTGAATCATCGAATCTTTGAACGCACATTGCGCCCGCCAGTATTCTGGCGGGCATGCCTGTTCGAGCGTCATTAC-
AACCCTCAGGCCCCCGGGCC-TGGCGTTGGGGATCGGCGGAGCGCCCCC-
TGCGGGCACACGCCGTCCCCCAAATACAGTGGCGGTCCCGCCGCAGCTTCCATTGCGTAGTAGCTA-
ACACCTCGCAACTGGAGAGCGGCGCGGCCACGCCGTAAAACACCCCGGTCAGAACGCCATCGTCGCTATCGCTTGCTACTCTGGTTACAACCA
GGAAGATTCCGTCATTATGAACCAGAGTAGTATCGATCGAGGCCTGTTCCGTAGTCTGTTCTTCAGATCCTACTCTGACCAGGAGAAGAAGGTC
GGTCTGAACTACACGGAAGTATTCGAGAAGCCCTTCCAGCAGTCGACGCTTCGTATGAAGCACGGTACCTACGACAAGCTGGACGAGGATGGT
ATCGTGGCCCCTGGTGTGCGAGTGTCAGGTGAAGATATCATCATCGGCAAGACTGCGCCGATTGATCAAGAGAACCAGGATCTGGGTACCAGG
ACAACGGTGCACCAGCGTCGTGATATCTCCACGCCGCTGCGAAGTACCGAGAACGGTATCGTCGATTCGGTCATTGTGACTGTCAATGCCGAC
AACGTCAAGTACGTCAAGGTCCGTGTGAGGACGACCAAGATTCCTCAGATTGGTGACAAGTTTGCCTCTCGTCACGGACAGAAGGGTACCATT
GGTGTCACCTACCGACAGGAGGATATGCCCTTTTCCAGGGAGGGTGTGACACCAGACATTATCATTAACCCCCACGCCATTCCGTCGCGAATG
ACAATTGCACATTTGATTGAATGCCTCCTCAGTAAGGTGTCAACACTCGAAGKCATGGAGGGTGATGCAACACCTTTCACCGATGTCACTGTCG
ACTCCGTTTCGGAGCTGCT
>FSSC28
TTGCTATCACACATCGAATTCCCCGTCGAATTCCCTC-------CTCCGCGACACGCTCCGCGCCCGCTTCTCCCGAGTCCCAAAAATTTTGCGGTT-
CGACCGTAATTTTTTT---GGTGGGGCATCT--ACCCCGCCACTCGGGCGACGTTGGACAAAGCCCTGATCCCTGCACACAAAAA-
CACCAAACCCTCT-TGGCGCGCATCA----CGTGGTTCACAACAGACACTGACTGGTTC-
AACAATAGGAAGCCGCTGAGCTCGGCAAGGGTTCCTTCAAGTACGCCTGGGTTCTTGACAAGCTCAAGGCCGAGCGTGAGCGTGGTATCATGA
ACTTACCTATA-CGTTGCTTCGGCGGGAA-TAGACG----GCCCCGTAA-AACGGGCCGCC--CCCGCCAGAGGACCCT-TAAC-TC---
TGTTTCTTTTAGTGTATCTTCTGAGTAAAACAAGCAAATAAATTAAAACTTTCAACAACGGATCTCTTGGCTCTGGCATCGATGAAGAACGCAG
CGAAATGCGATAAGTAATGTGAATTGCAGAATTCAGTGAATCATCGAATCTTTGAACGCACATTGCGCCCGCCAGTATTCTGGCGGGCATGCCT
GTTCGAGCGTCATTAC-AACCCTCAGGCCCCCGGGCC-TGGCGTTGGGGATCGGCG--GAGC-
CCCCCGTGGGCACACGCCGTCCCCCAAATACAGTGGCGGTCCCGCCGCAGCTTCCATCGCGTAGTAGCTA-
ACACCTCGCGACTGGAGAGCGGCGCGGCCACGCCGTAAAACACCCCGGTCAGAACGCCATCGTCGCTATCGCTTGCTACTCTGGTTATAACCA
GGAAGATTCCGTCATTATGAACCAGAGTAGTATCGATCGAGGCCTGTTCCGCAGTCTGTTCTTCCGATCTTACTCTGACCAAGAGAAGAAGGTT
GGTCTGAACTACACGGAAGTGTTTGAGAAGCCCTTCCAGCAGTCGACGCTTCGTATGAAGCACGGTACCTACGACAAGCTGGATGAGGATGGT
ATCGTGGCTCCCGGTGTGCGAGTGTCGGGTGAAGATATCATCATCGGCAAGACTGCGCCGATTGATCAAGAGAACCAGGATCTGGGTACCAGG
ACAACGGTGCACCAGCGTCGTGATATCTCCACGCCGCTGCGAAGTACCGAGAACGGTATCGTCGATTCGGTCATTGTGACTGTCAATGCCGAC
AACGTCAAGTATGTCAAGGTCCGTGTGAGGACGACCAAGATTCCTCAGATTGGTGACAAGTTCGCCTCTCGTCACGGACAGAAGGGTACCATT
GGTGTTACCTACCGACAGGAGGATATGCCCTTTAGCAGGGAGGGTGTGACACCAGACATTATCATTAACCCCCACGCCATTCCGTCGCGAATG
ACAATTGCCCATTTGATTGAATGCCTCCTCAGTAAGGTGTCAACGCTCGAAGGCATGGAGGGTGATGCCACACCTTTCACCGACGTCACTGTCG
ACTCCGTTTCGGAGCTGCT
>CPC27700
TC-CTT-TGCCCATCGATTT----------CC--CCTA------CGACTCGAAACGTGCCCG--CTACCCCGCTCGAGACC-AAAAATTTTGCAATA-
TGACCGTAATTTTTTT---GGTGGGGCAC-TT-ACCCCGCCACTTGAGCGACGGGAGCGTTTGCCCTCTTA-C---------CATTCTCACAACCTCA--
ATGAGTGCGTCGT-CATGTG-TCAAG--CAGTCACTAACCATTC--
AACAATAGGAAGCCGCTGAGCTCGGTAAGGGTTCCTTCAAGTACGCCTGGGTTCTTGACAAGCTCAAGGCCGAGCGTGAGCGTGGTATCATGA
ACATACCAC-T-TGTTGCCTCGGCGG--ATCAGCCC--GCTCCCGGTAA-AACGGGACGG---CCCGCCAGAGGACCCCTAAAC-TC---TGTTTCT--
ATATGTAACTTCTGAGTAAAACCA-
TAAATAAATCAAAACTTTCAACAACGGATCTCTTGGTTCTGGCATCGATGAAGAACGCAGCAAAATGCGATAAGTAATGTGAATTGCAGAATT
CAGTGAATCATCGAATCTTTGAACGCACATTGCGCCCGCCAGTATTCTGGCGGGCATGCCTGTTCGAGCGTCATTTC-AACCCTCAAGCAC---
AGCT-TGGTGTTGGGACTCG------CG--TTAATTCG---------CGTTCCTCAAATTGATTGGCGGTCACGTCG-AGCTTCCATAGCGTAGTAGTAAAAC-
CCTCGTTACTGGTAATCGTCGCGGCCACGCCGTTAAACC-
CCCGGTCAAAACGCCATTGTTGCAATTGCTTGCTACTCAGGTTACAACCAGGAAGATTCCGTCATTATGAACCAGAGTAGTATTGATCGAGGTC
TGTTCCGAAGTCTGTTCTTCCGATCGTACTCAGATCAGGAGAAGAAGGTTGGTCTCAACTACACTGAGATCTTCGAGAAACCTTTCCAGCAGAC
AACGCTTCGAATGAAGCATGGAACATACGACAAGCTTGATGAAGATGGTATCGTGGCTCCTGGTGTCCGTGTGTCAGGTGAAGATATCATTATC
GGCAAGACTGCACCCATCGACCAAGAAAATCAGGACCTTGGCACAAGAACTCAATCGCACCAACGTCGTGATATCTCGACACCACTGCGAAG
TACTGAGAACGGTATCGTTGATCAAGTCATTCTGACAGTCAACGCCGATAACGTCAAGTACGTCAAGGTCCGAGTACGAACAACCAAGATTCC
TCAAATTGGTGACAAGTTTGCTTCTCGTCACGGTCAAAAGGGTACAATCGGTGTTACATATCGACAGGAGGATATGCCTTTCAGCCGAGAAGGT
CTTACTCCCGATATCATTATCAACCCTCACGCCATTCCATCGCGAATGACAATTGCCCATTTGATTGAGTGTCTTCTTAGCAAGGTTTCAACGCT
GGAAGGTATGGAGGGTGACGCCACACCGTTCACTGATGTCACAGTCGATTCAGTCTCAGAACTTCT
>CPC27701
TC-CTT-TGCCCATCGATTT----------CC--CCTA------CGACTCGAAACGTGCCCG--CTACCCCGCTCGAGACC-AAAAATTTTGCAATA-
TGACCGTAATTTTTTT---GGTGGGGCAC-TT-ACCCCGCCACTTGAGCGACGGGAGCGTTTGCCCTCTTA-C---------CATTCTCACAACCTCA--
ATGAGTGCGTCGT-CATGTG-TCAAG--CAGTCACTAACCATTC--
AACAATAGGAAGCCGCTGAGCTCGGTAAGGGTTCCTTCAAGTACGCCTGGGTTCTTGACAAGCTCAAGGCCGAGCGTGAGCGTGGTATCATGA
ACATACCAC-T-TGTTGCCTCGGCGG--ATCAGCCC--GCTCCCGGTAA-AACGGGACGG---CCCGCCAGAGGACCCCTAAAC-TC---TGTTTCT--
ATATGTAACTTCTGAGTAAAACCA-
TAAATAAATCAAAACTTTCAACAACGGATCTCTTGGTTCTGGCATCGATGAAGAACGCAGCAAAATGCGATAAGTAATGTGAATTGCAGAATT
CAGTGAATCATCGAATCTTTGAACGCACATTGCGCCCGCCAGTATTCTGGCGGGCATGCCTGTTCGAGCGTCATTTC-AACCCTCAAGCAC---
AGCT-TGGTGTTGGGACTCG------CG--TTAATTCG---------CGTTCCTCAAATTGATTGGCGGTCACGTCG-AGCTTCCATAGCGTAGTAGTAAAAC-
CCTCGTTACTGGTAATCGTCGCGGCCACGCCGTTAAACC-
CCCGGTCAAAACGCCATTGTTGCAATTGCTTGCTACTCAGGTTACAACCAGGAAGATTCCGTCATTATGAACCAGAGTAGTATTGATCGAGGTC
TGTTCCGAAGTCTGTTCTTCCGATCGTACTCAGATCAGGAGAAGAAGGTTGGTCTCAACTACACTGAGATCTTCGAGAAACCTTTCCAGCAGAC
AACGCTTCGAATGAAGCATGGAACATACGACAAGCTTGATGAAGATGGTATCGTGGCTCCTGGTGTCCGTGTGTCAGGTGAAGATATCATTATC
GGCAAGACTGCACCCATCGACCAAGAAAATCAGGACCTTGGCACAAGAACTCAATCGCACCAACGTCGTGATATCTCGACACCACTGCGAAG
TACTGAGAACGGTATCGTTGATCAAGTCATTCTGACAGTCAACGCCGATAACGTCAAGTACGTCAAGGTCCGAGTACGAACAACCAAGATTCC
TCAAATTGGTGACAAGTTTGCTTCTCGTCACGGTCAAAAGGGTACAATCGGTGTTACATATCGACAGGAGGATATGCCTTTCAGCCGAGAAGGT

CTTACTCCCGATATCATTATCAACCCTCACGCCATTCCATCGCGAATGACAATTGCCCATTTGATTGAGTGTCTTCTTAGCAAGGTTTCAACGCT
GGAAGGTATGGAGGGTGACGCCACACCGTTCACTGATGTCACAGTCGATTCAGTCTCAGAACTTCT
>CPC27702
TC-CTT-TGCCCATCGATTT----------CC--CCTA------CGACTCGAAGCGTGCCCG--CTACCCCGCTCGAGACC-AAGAATCTTGCAATA-
TGACCGTAATTTTTTT---GGTGGGGCAC-TT-ACCCCGCCACTTGAGCGACGGGAGCGTTTGCCCTCTTAAC---------CATTCTCACAACCTCA--
ATGAGTGCGTCGT-CACGTG-TCAAG--CAGTCACTAACCATTC--
AACAATAGGAAGCCGCTGAGCTCGGTAAGGGTTCCTTCAAGTACGCCTGGGTTCTTGACAAGCTCAAGGCCGAGCGTGAGCGTGGTATCATGA
ACATACCAC-T-TGTTGCCTCGGCGG--ATCAGCCC--GCTCCCGGTAA-AACGGGACGG---CCCGCCAGAGGACCCCTAAAC-TC---TGTTTCT--
ATATGTAACTTCTGAGTAAAACCA-
TAAATAAATCAAAACTTTCAACAACGGATCTCTTGGTTCTGGCATCGATGAAGAACGCAGCAAAATGCGATAAGTAATGTGAATTGCAGAATT
CAGTGAATCATCGAATCTTTGAACGCACATTGCGCCCGCCAGTATTCTGGCGGGCATGCCTGTTCGAGCGTCATTTC-AACCCTCAAGCAC---
AGCT-TGGTGTTGGGACTCG------CG--TTAATTCG---------CGTTCCTCAAATTGATTGGCGGTCACGTCG-AGCTTCCATAGCGTAGTAGTAAAAC-
CCTCGTTACTGGTAATCGTCGCGGCCACGCCGTTAAACC-
CCCGGTCAAAACGCCATTGTTGCAATTGCTTGCTACTCAGGTTACAACCAGGAAGATTCCGTCATTATGAACCAGAGTAGTATTGATCGAGGTC
TGTTCCGAAGTCTGTTCTTCCGATCGTACTCAGATCAGGAGAAGAAGGTTGGTCTCAACTACACTGAGATCTTTGAGAAACCTTTCCAGCAGAC
AACGCTTCGAATGAAGCATGGAACATACGACAAGCTTGATGAAGATGGTATCGTGGCTCCTGGTGTCCGTGTGTCAGGTGAAGATATCATTATC
GGCAAGACTGCACCCATCGACCAAGAAAACCAGGACCTTGGCACAAGAACTCAATCGCACCAGCGTCGTGATATCTCGACACCACTGCGAAG
TACTGAGAACGGTATCGTTGATCAAGTCATTCTGACAGTCAACGCCGATAACGTTAAGTACGTCAAGGTCCGAGTACGAACAACCAAGATTCC
TCAAATTGGTGACAAGTTTGCTTCTCGTCACGGTCAAAAGGGTACAATCGGTGTTACATATCGACAGGAGGATATGCCTTTCAGCCGAGAAGGT
CTTACTCCCGATATCATTATCAACCCTCACGCCATTCCATCGCGAATGACAATTGCCCATTTGATTGAGTGTCTTCTTAGCAAGGTTTCAACGCT
GGAAGGTATGGAGGGTGACGCCACACCGTTCACTGATGTCACAGTCGATTCAGTCTCAGAACTTCT
>CPC28190
TC-CTT-TGCCCATCGATTT----------CC--CCTA------CGACTCGAAACGTGCCCG--CTACCCCGCTCGAGACC-AAAATTTTTGCAATA-
TGACTGTAATTTTTTT---GGTGGGGCAC-TT-ACCCCGCCACTTGAGCGACGGGAGCGTTTGCCCTCTTAAC---------CATTCTCACAACCTCA--
ATGAGTGCGTCGT-CACGTG-TCAAG--CAGTCACTAACCATTC--
AACAATAGGAAGCCGCTGAGCTCGGTAAGGGTTCCTTCAAGTACGCCTGGGTTCTTGACAAGCTCAAGGCCGAGCGTGAGCGTGGTATCATGA
ACATACCAC-T-TGTTGCCTCGGCGG--ATCAGCCC--GCTCCCGGTAA-AACGGGACGG---CCCGCCAGAGGACCCCTAAAC-TC---TGTTTCT--
ATATGTAACTTCTGAGTAAAACCA-
TAAATAAATCAAAACTTTCAACAACGGATCTCTTGGTTCTGGCATCGATGAAGAACGCAGCAAAATGCGATAAGTAATGTGAATTGCAGAATT
CAGTGAATCATCGAATCTTTGAACGCACATTGCGCCCGCCAGTATTCTGGCGGGCATGCCTGTTCGAGCGTCATTTC-AACCCTCAAGCAC---
AGCT-TGGTGTTGGGACTCG------CG--TTAATTCG---------CGTTCCCCAAATTGATTGGCGGTCACGTCG-AGCTTCCATAGCGTAGTAGTAAAAC-
CCTCGTTACTGGTAATCGTCGCGGCCACGCCGTTAAACC-
CCCGGTCAAAACGCCATTGTTGCAATTGCTTGCTACTCAGGTTACAACCAGGAAGATTCCGTCATTATGAACCAGAGTAGTATTGATCGAGGTC
TGTTCCGAAGTCTGTTCTTCCGATCGTACTCAGATCAGGAGAAGAAGGTTGGTCTCAACTACACTGAGATCTTTGAGAAACCTTTCCAGCAGAC
AACGCTTCGAATGAAGCATGGAACATACGACAAGCTTGATGAAGATGGTATCGTGGCTCCTGGTGTCCGTGTGTCAGGTGAAGATATCATTATC
GGCAAGACTGCACCCATCGACCAAGAAAACCAGGACCTTGGCACAAGAACTCAATCGCACCAGCGTCGTGATATCTCGACACCACTGCGAAG
TACTGAGAACGGTATCGTTGATCAAGTCATTCTGACAGTCAACGCCGATAACGTTAAGTACGTCAAGGTCCGAGTACGAACAACCAAGATTCC
TCAAATTGGTGACAAGTTTGCTTCTCGTCACGGTCAAAAGGGTACAATCGGTGTTACATATCGACAGGAGGATATGCCTTTCAGCCGAGAAGGT
CTTACTCCCGATATCATTATCAACCCTCACGCCATTCCATCGCGAATGACAATTGCCCATTTGATTGAGTGTCTTCTTAGCAAGGTTTCAACGCT
GGAAGGTATGGAGGGTGACGCCACACCGTTCACTGATGTCACAGTCGATTCAGTCTCAGAACTTCT

Figure S2: Phylogenetic tree construction using the maximum parsimony method based on a concatenated dataset of TEF-1α sequence of 52 strains belonging to the *Fusarium* species complex using MEGA v11. Ex-epitype of *F.oxysporum* are indicated in red font. The branch lengths were proportional to the distance. *Fusicolla aquaeductuum* (NRRL 20686) was used as an outgroup.

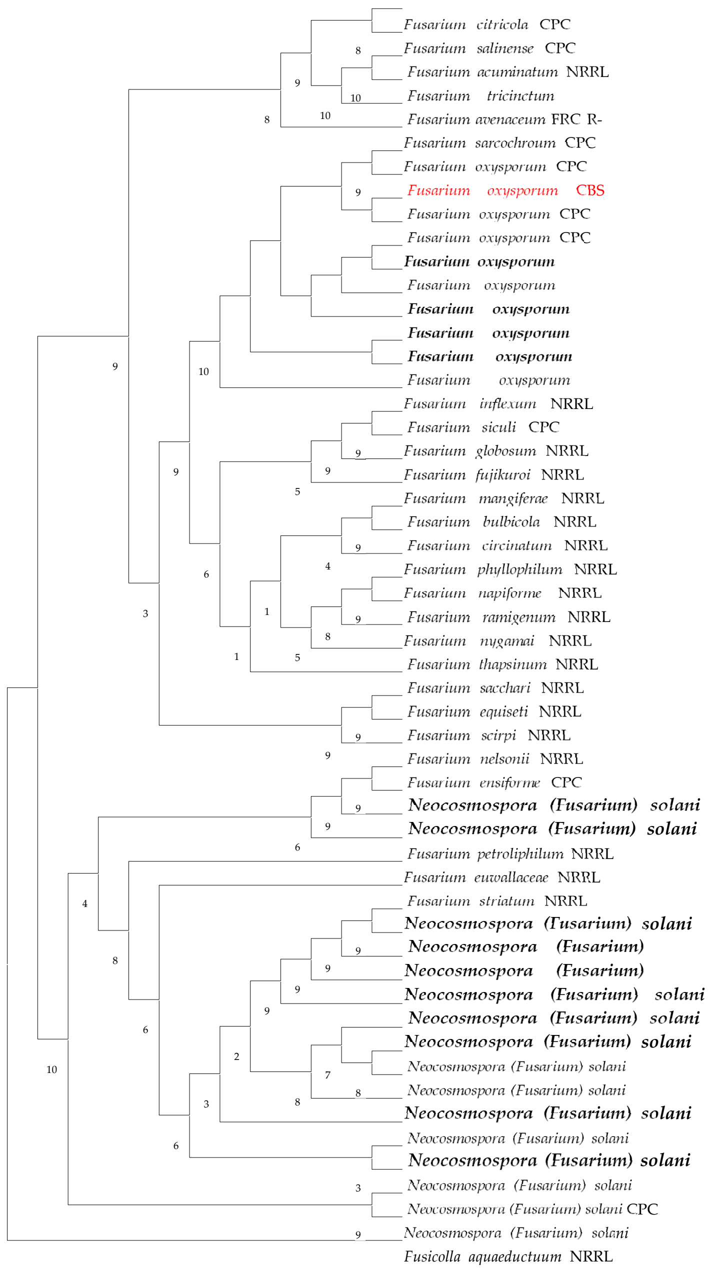

Supplement: Supplementary file 1 [file plants-14-00376-s001.zip › plants-3365897-supplementary.pdf]
